# FADD is a key regulator of lipid metabolism

**DOI:** 10.15252/emmm.201505924

**Published:** 2016-06-29

**Authors:** Hongqin Zhuang, Xueshi Wang, Daolong Zha, Ziyi Gan, Fangfang Cai, Pan Du, Yunwen Yang, Bingya Yang, Xiangyu Zhang, Chun Yao, Yuqiang Zhou, Chizhou Jiang, Shengwen Guan, Xuerui Zhang, Jing Zhang, Wenhui Jiang, Qingang Hu, Zi‐Chun Hua

**Affiliations:** ^1^ The State Key Laboratory of Pharmaceutical BiotechnologyCollege of Life Science and School of StomatologyAffiliated Stomatological HospitalNanjing UniversityNanjingChina; ^2^ Changzhou High‐Tech Research Institute of Nanjing University and Jiangsu TargetPharma Laboratories Inc.ChangzhouChina

**Keywords:** FADD, lipid metabolism, obesity, PPAR‐α, Metabolism

## Abstract

FADD, a classical apoptotic signaling adaptor, was recently reported to have non‐apoptotic functions. Here, we report the discovery that FADD regulates lipid metabolism. PPAR‐α is a dietary lipid sensor, whose activation results in hypolipidemic effects. We show that FADD interacts with RIP140, which is a corepressor for PPAR‐α, and FADD phosphorylation‐mimic mutation (FADD‐D) or FADD deficiency abolishes RIP140‐mediated transcriptional repression, leading to the activation of PPAR‐α. FADD‐D‐mutant mice exhibit significantly decreased adipose tissue mass and triglyceride accumulation. Also, they exhibit increased energy expenditure with enhanced fatty acid oxidation in adipocytes due to the activation of PPAR‐α. Similar metabolic phenotypes, such as reduced fat formation, insulin resistance, and resistance to HFD‐induced obesity, are shown in adipose‐specific FADD knockout mice. Additionally, FADD‐D mutation can reverse the severe genetic obesity phenotype of *ob*/*ob* mice, with elevated fatty acid oxidation and oxygen consumption in adipose tissue, improved insulin resistance, and decreased triglyceride storage. We conclude that FADD is a master regulator of glucose and fat metabolism with potential applications for treatment of insulin resistance and obesity.

## Introduction

Metabolic syndrome is an epidemic affecting a large percentage of the population of the world. Obesity in adults as well as children is a global health problem, resulting from an imbalance between caloric intake and energy expenditure. The increasing prevalence of the metabolic syndrome and obesity calls for new approaches for the prevention and management of these diseases. Precise control of energy expenditure and food intake is required to maintain energy homeostasis (Spiegelman & Flier, 2001). In mammals, triglycerides (TGs) in adipose tissues are a major form of energy storage. Unburned energy is stored at TGs in adipose tissues and subsequently in other organs such as the liver, when the energy consumption exceeds the calories combustion, leading to the development of obesity over time (Duncan *et al*, 2007). Metabolic syndrome, including type 2 diabetes mellitus, atherosclerosis, and cancer, may result from obesity (Evans *et al*, 2004; Gurevich‐Panigrahi *et al*, 2009). When TG synthesis exceeds lipolysis, it will lead to elevated TG storage, resulting in adipocyte hypertrophy and subsequently obesity. Hormones, which are secreted according to the nutritional status, tightly regulate lipolysis within adipocytes. Catecholamines stimulate lipolysis during fasting via increasing cyclic AMP (cAMP) levels. However, insulin inhibits lipolysis in the fed state (Duncan *et al*, 2007; Jaworski *et al*, 2007). To date, most of the enzymes that regulate TG metabolism have been identified; however, signaling pathway controlling this process is still not well understood. Because of the high prevalence of dyslipidemia in the general population (Ginsberg *et al*, 2006), it is important to identify signaling factors regulating metabolic and regulatory aspects of the TG metabolism.

Fas‐associated death domain‐containing protein (FADD) is the key adaptor protein transmitting apoptotic signals mediated by death receptors (Chinnaiyan *et al*, 1995). Apart from its critical role in death receptor signaling of apoptosis, FADD also participates in cell cycle progression (Matsuyoshi *et al*, 2006; Osborn *et al*, 2007), lymphocyte proliferation and activation (Zhang *et al*, 1998; Hua *et al*, 2003), embryonic development (Yeh *et al*, 1998; Zhang *et al*, 2011), innate immunity (Imtiyaz *et al*, 2006; Zhande *et al*, 2007), and chemosensitization (Shimada *et al*, 2005). Previous studies have revealed that these non‐apoptotic activities of FADD are partly due to its phosphorylation (serine 194 in human and serine 191 in mouse) (Zhang *et al*, 2004; Alappat *et al*, 2005), which is at the C‐terminal tail of FADD outside apoptotic domains and is regulated dependent on the cell cycle (Scaffidi *et al*, 2000). Mice bearing the mutation mimicking constitutive phosphorylation (S191D) of FADD are immunologically compromised, similar to FADD‐deficient mice (Hua *et al*, 2003). FADD is located on chromosome 11q13.3, a diabetes susceptibility locus; therefore, it has been suspected that alterations in FADD might result in diabetes (Kim *et al*, 1996).

Our previous studies found that FADD may participate in energy metabolism, especially fatty acid oxidation (Zhuang *et al*, 2013a,b), but the mechanisms are poorly understood. It is well established that PPAR‐α controls fatty acid oxidation by regulating genes involved in fatty acid transport, fatty acid oxidation, and mitochondrial respiration (Minnich *et al*, 2001). Here, we report that FADD is required for the PPAR‐α to recruit the transcriptional corepressor RIP140. In the absence of FADD, PPAR‐α no longer interacted with RIP140 and therefore was activated. Interestingly, the S191D mutation abolished the ability of FADD to promote PPAR‐α recruiting RIP140 and promoted the activation of PPAR‐α. Both FADD deficiency and S191D mutation enhanced PPAR‐α transcriptional activation and led to the subsequently enhancement of fatty acid oxidation. Complementary studies in mice bearing a mutation form of FADD mimicking constitutive phosphorylation at serine 191 (FADD‐D) or adipose‐specific FADD knockout mice revealed global alterations in energy and fuel metabolism, adiposity, and glucose–insulin homeostasis. FADD‐D mice showed decreased fat in adipose tissue, liver, and muscle. These mice consumed even more food than did the wild‐type, yet lost body weight. Moreover, FADD‐D mutation was associated with prevention of genetic obesity, such as obesity resulting from leptin deficiency, due to enhanced energy expenditure caused by PPAR‐α‐mediated upregulation of genes participating in fatty acid oxidation in adipose tissues. In addition, the S191D mutation of FADD in *ob*/*ob* mice led to the improvement of insulin sensitivity and glucose tolerance. Using the adipose‐specific aP2‐Cre transgene, adipose‐specific deletion (ad‐FADD) mice were created via the Cre/LoxP system to further study the specific role of FADD in insulin resistance and obesity. Ad‐FADD mice exhibited similar metabolic phenotypes with FADD‐D mice, including reduced fat formation, decreased adipose tissue inflammation, insulin resistance, and resistance to high‐fat diet (HFD)‐caused obesity. These results raise an unrevealed function of a canonical death protein FADD and the possibility that pharmacological manipulation of FADD may lead to loss of body fat in the context of normal caloric intake.

## Results

### FADD regulates PPAR‐α's activation

**Figure 1:**
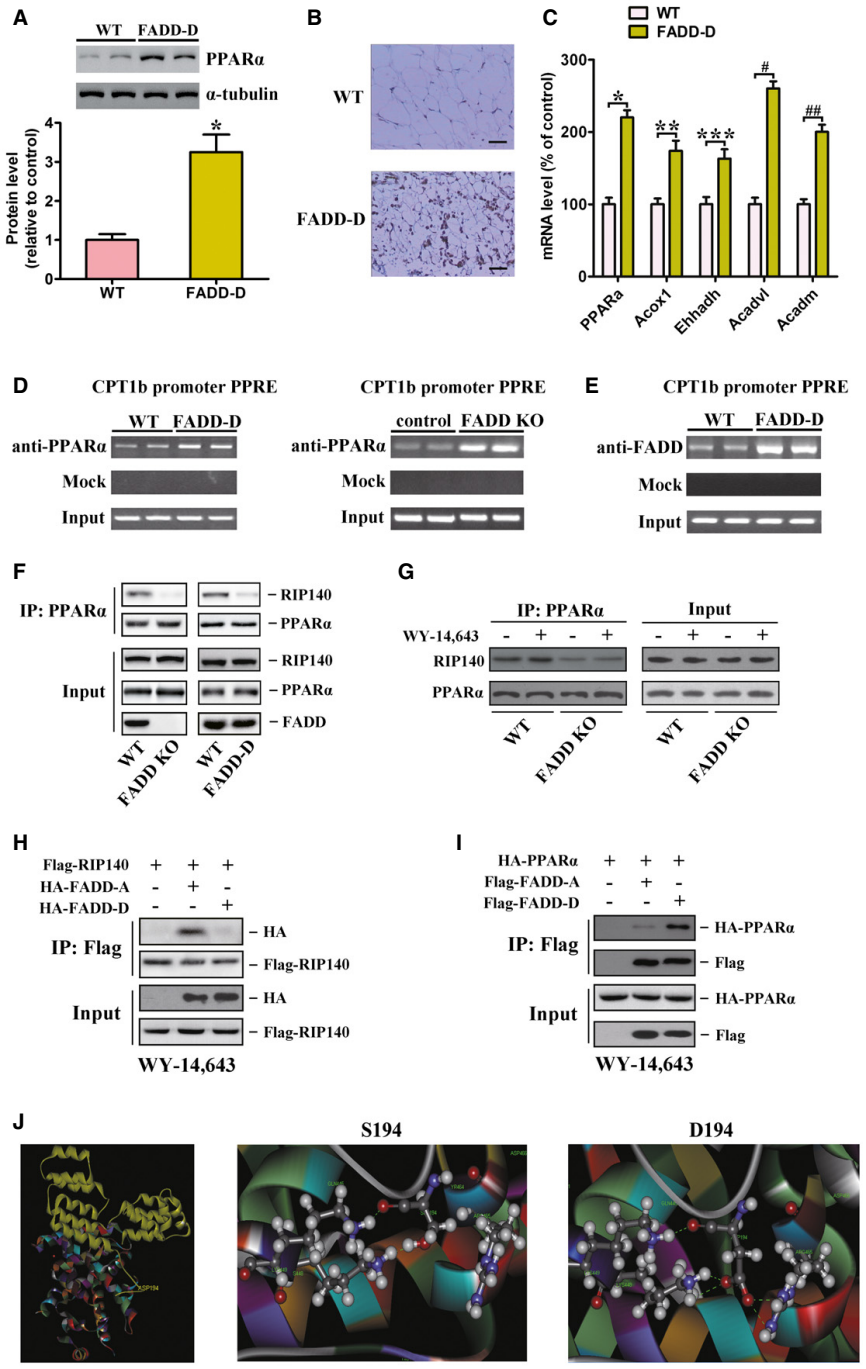
FADD regulates the interaction of PPAR‐α and RIP140 Immunoblot of PPAR‐α and tubulin (control) (top) in WAT from WT and FADD‐D mice with relative quantification (bottom). Data are expressed as mean ± SEM from three independent experiments. *P =* *0.0035 (Student's *t*‐test).Immunostaining of PPAR‐α expression in WAT from WT and FADD‐D mice (Scale bars = 50 μm). Shown are typical results from four different fields and three different experiments.Quantitative real‐time PCR for *PPAR‐*α and PPAR‐α target genes, using RNA from epididymal fat from 10‐week‐old male WT and FADD‐D mice fed a SD. Data are expressed as mean ± SEM from two independent experiments (*n* = 6 total for each genotype). *P =* *0.0022, **P =* *0.0083, ***P =* *0.0107, ^#^ *P *=* *0.0011, ^##^ *P *=* *0.0037 (Student's *t*‐test).ChIP assay of CPT1b promoter in adipocytes isolated from WT, FADD‐D, and ad‐FADD mice. Soluble chromatin from adipocytes was immunoprecipitated with control mouse IgG or antibodies against PPAR‐α. The experiments were performed in triplicate.ChIP assay of CPT1b promoter in adipocytes isolated from WT and FADD‐D mice. Soluble chromatin from adipocytes was immunoprecipitated with control mouse IgG or antibodies against FADD. The experiments were performed in triplicate.PPAR‐α and RIP140 coimmunoprecipitate in WT but not FADD knockout or FADD‐D MEFs. PPAR‐α was immunoprecipitated (IP) from the cell lysates of WT or FADD knockout MEFs. Data shown are representative of three independent experiments having similar results.PPAR‐α and RIP140 coimmunoprecipitate in WT but not FADD knockout MEFs upon WY‐14,643 stimulation. Ligand‐dependent interactions were examined in the presence of 100 μM WY‐14,643 where indicated. Data shown are representative of three independent experiments having similar results.Coimmunoprecipitation analysis of FADD and RIP140 in 293T cells. As indicated, lysates from the cells transfected with plasmids encoding Flag‐RIP140, HA‐FADD‐A, or HA‐FADD‐D were subjected to immunoprecipitation with an anti‐Flag antibody. FADD‐A and FADD‐D were detected using an anti‐HA antibody. Data shown are representative of three independent experiments having similar results.Coimmunoprecipitation analysis of FADD and PPAR‐α in 293T cells. As indicated, lysates from the cells transfected with plasmids encoding Flag‐FADD‐A, Flag‐FADD‐D, or HA‐PPAR‐α were subjected to immunoprecipitation with an anti‐Flag antibody. PPAR‐α was visualized by anti‐HA. Data shown are representative of three independent experiments having similar results.Left, the putative complex of FADD (yellow) and PPAR‐α (colorful) by molecular modeling is portrayed in ribbon representation. Middle, the residue S194 of FADD and PPAR‐α residue K448 are shown in sphere representation, which is immediately proximal and capable of hydrogen‐bonding interactions. Right, the key residue D194 of FADD‐D and PPAR‐α residue Q445, K448, K449, and R465 are shown in sphere representation, which is immediately proximal and capable of salt bridging interactions. Source data are available online for this figure.

PPAR‐α has a central role in fatty acid oxidation and lipid metabolism. Our previous studies found that MEFs bearing constitutive phosphorylated FADD mimic (FADD‐D) or FADD deficiency (FADD KO) exhibited enhanced fatty acid β‐oxidation (Zhuang *et al*, 2013a,b). It remains unclear, however, if FADD‐D mutation or FADD deficiency has an effect on the regulation of PPAR‐α. Here, we found that both gene expression and protein level of PPAR‐α were increased in FADD KO and FADD‐D MEFs (Appendix Fig S1A and B). Moreover, proteins involved in mitochondrial fatty acid oxidation systems were found to be strongly increased in both FADD‐D and FADD KO MEFs as compared to wild‐type (WT) MEFs, including very long‐chain acyl‐CoA dehydrogenases (VLCAD), long‐chain acyl‐CoA dehydrogenases (LCAD), and medium‐chain acyl‐CoA dehydrogenases (MCAD) (Appendix Fig S1C), indicating transcriptional activation of PPAR‐α‐regulated genes involved in fatty acid oxidation. Consistent with the data obtained from MEFs, both gene expression and protein level of PPAR‐α were increased in white adipose tissue (WAT) of FADD‐D mice (Fig 1A–C). Moreover, FADD‐D mutation increased the WAT expression of PPAR‐α target genes involved in fatty acid β‐oxidation, including enoyl‐CoA hydratase/3‐hydroxyacyl‐CoA dehydrogenase (*Ehhadh*, also known as *HD*), acyl‐CoA oxidase 1 (*Acox1*), medium‐chain acyl‐CoA dehydrogenase (*Acadm*), and very long‐chain acyl‐CoA dehydrogenase (*Acadvl*) (Fig 1C). To confirm whether PPAR‐α was recruited to the promoter region, which contains an identified PPAR‐response element (PPRE), of gene related to fatty acid oxidation in adipocytes, we performed ChIP assay using *CPT1b* as a fatty acid oxidation gene. As shown in Fig 1D, ChIP assay resulted in the binding of PPAR‐α to the promoter regions of *CPT1b* identified in WT adipocytes, and the binding was increased in FADD‐D and FADD KO adipocytes. Similar results were obtained in FADD KO 3T3L1 adipocytes (Appendix Fig S1D). In addition, FADD was also found to be recruited to PPRE in the *CPT1b* promoter in WT adipocytes, and this binding was further enhanced in FADD‐D adipocytes (Fig 1E). It suggests that FADD‐D mutation or FADD knockout enhances the direct binding of PPAR‐α to PPRE. RIP140 was identified as a transcriptional corepressor for nuclear receptors (Treuter *et al*, 1998). To further study the effect of FADD on PPAR‐α transcriptional activation, we examined the possibility that FADD regulates the association of RIP140 with PPAR‐α. Interestingly, RIP140 coprecipitated with PPAR‐α in WT MEFs, but much less RIP140 coprecipitated with PPAR‐α in FADD knockout or FADD‐D MEFs with or without WY‐14,643, a selective PPAR‐α agonist (Fig 1F and G). Similar results were obtained in FADD knockout 3T3L1 adipocytes (Appendix Fig S1E). These data indicate that FADD is required for PPAR‐α to recruit RIP140 and FADD‐D mutation impairs the interaction between PPAR‐α and RIP140. In addition, FADD‐D failed to interact with RIP140 while FADD or FADD‐A was found to be associated with RIP140 (Fig 1H and Appendix Fig S1F). Paradoxically, the interaction between FADD‐D and PPAR‐α was enhanced as compared with that of FADD or FADD‐A (Fig 1I and Appendix Fig S1G). Molecular modeling analysis of FADD and PPAR‐α association further predicted that FADD S194 was in close enough proximity to PPAR‐α. Moreover, mutation of S194 to D194 increased protein complex thermostability by formation of stable salt bridges between FADD D194 and PPAR‐α Q445, K448, K449 and R465 instead of hydrogen bonding between FADD S194 and PPAR‐α K448 (Fig 1J). Mutation of PPAR‐α K449 and R465 to alanine resulted in reduced interaction between FADD‐D and PPAR‐α (Appendix Fig S1H). To further clarify the specific role of FADD on PPAR‐α's activation, we examined the influence of FADD on the rates of oleic acid oxidation and the mRNA level of PPAR‐α in the presence or absence of RIP140. We found that in the presence of RIP140, FADD KO 3T3L1 adipocytes exhibited more than twofold the rate of oleic acid oxidation found in WT cells, whereas the rates of oleic acid oxidation were identical between FADD KO and WT 3T3L1 adipocytes following the downregulation of RIP140 (Appendix Fig S1I, left). Similarly, FADD deficiency resulted in upregulation of PPAR‐α mRNA level in the presence of RIP140, but had no effect when RIP140 was downregulated (Appendix Fig S1I, right). Efficiency of the RIP140 knockdown in 3T3L1 adipocytes was shown by immunoblot. These results indicate that the ability of FADD to regulate PPAR‐α is RIP140 dependent and suggest a critical role of phosphorylation in regulating the novel function of FADD (Appendix Fig S1J).

### FADD‐D mice have reduced adiposity and are resistant to diet‐induced obesity

**Figure 2:**
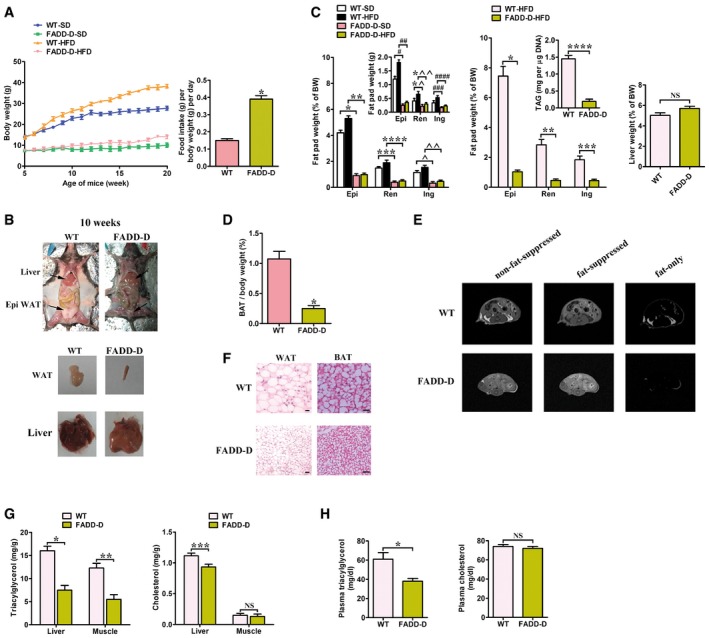
Body weights and metabolic phenotype of FADD‐D mice Left, body weights of male WT and FADD‐D mice on either a SD (*n* = 8 for each genotype) or a HFD (*n* = 6 for each genotype). Right, food intake per mouse fed a HFD measured over 20 days normalized by body weight (*n* = 10 for each genotype). Results are means ± SEM. *P =* *0.0007 (Student's *t*‐test).Representative photographs of fat pads and organs of 10‐week‐old male FADD‐D and WT mice.Left, fat pad weights as a percentage of body weight (BW) and in absolute amounts (inset) from male FADD‐D and WT mice. Mice were fed a SD or a HFD until 15 weeks of age (*n* = 6 for each genotype). Results are means ± SEM. *P =* *0.0012, **P =* *0.0002, ***P =* *0.0027, ****P =* *0.0014, ^*P *=* *0.0038, ^^*P *=* *0.0019, ^#^ *P *=* *0.0045, ^##^ *P *=* *0.0007, *^*P *=* *0.0252, *^^*P *=* *0.0082, ^###^ *P *=* *0.0275, ^####^ *P *=* *0.0071 (one‐way ANOVA). Middle, fat pad weights of male FADD‐D and WT mice on a HFD at 30 weeks of age (*n* = 6 for each genotype). Inset, triacylglycerol (TAG) content in epididymal WAT. Data are expressed as means ± SEM. *P =* *0.0003, **P =* *0.0005, ***P =* *0.0019, ****P =* *0.0011 (Student's *t*‐test). Right, weight of liver normalized by body weight for male FADD‐D and WT littermates at the age of 15 weeks (*n* = 6 for each genotype). Data are expressed as means ± SEM. NS, not statistically significant (Student's *t*‐test). Epi, epididymal fat; Ren, renal fat; Ing, inguinal fat.Weight of brown adipose tissue normalized by body weight (*n* = 5 for each genotype). Data are expressed as means ± SEM. *P =* *0.0008 (Student's *t*‐test).MRI of body fat content: standard non‐fat suppressed (left), fat suppressed (middle,) and fat only (right). Representative images of three independent experiments (*n* = 6 total for each genotype) showing similar results are shown.Paraffin‐embedded sections of WAT and BAT from WT and FADD‐D mice were stained with H&E. Scale bar = 50 μm. Shown are typical results from four different fields and three different experiments.Left, relative amounts of liver and muscle triacylglycerol contents in 10‐month‐old WT and FADD‐D mice (*n* = 6 for each genotype). Right, relative amounts of cholesterol in the liver and muscle of the same group of WT and FADD‐D mice (*n* = 6 for each genotype). Data are expressed as means ± SEM. *P =* *0.0015, **P =* *0.0027, ***P =* *0.0318 (Student's *t*‐test). NS, not statistically significant.Left, plasma triacylglycerol levels in 10‐month‐old WT and FADD‐D mice following fasting for 20 h (*n* = 5 for each genotype). Right, plasma cholesterol levels in the same group of WT and FADD‐D mice (*n* = 5 for each genotype). Data are expressed as means ± SEM. *P =* *0.0124 (Student's *t*‐test). NS, not statistically significant.

Because PPAR‐α plays a critical role in lipid metabolism, we hypothesized that FADD phosphorylation‐mediated regulation of PPAR‐α might contribute to the fat distribution of human or mice. Indeed, the level of phosphorylated FADD was obviously reduced in WAT of obese human and *ob*/*ob* obese mice (Appendix Fig S2A and B). As shown previously (Hua *et al*, 2003), FADD can appear as both slower migrating phosphorylated and faster nonphosphorylated bands in metabolic tissues, while only the slower FADD species was detected in FADD‐D mice (Appendix Fig S2C). FADD‐D mice exhibited apparent growth retardation, which was evident in body length and body weight (Cheng *et al*, 2014). A matched set of WT and FADD‐D mice were given a standard chow diet (SD) over 15 weeks from 5 weeks of age to evaluate the effect of age on growth. FADD‐D mutation significantly reduced the rate at which the mice increased their body weight on the SD relative to the WT mice. At 20 weeks of age, there was a 64% difference in average body weight (Fig 2A). FADD‐D mice that were fed a high‐fat diet (HFD) also had decreased weight gain (Fig 2A). These differences could not be due to a reduction in food consumption because it was increased 1.6‐fold in the FADD‐D mice when food consumption was adjusted for body weight (Fig 2A, right). Food consumption was also increased in FADD‐D mice before feeding a HFD (Appendix Fig S3). Lower body weight in the FADD‐D mice might be attributable to the reduction of WAT weight (Fig 2B and C) and also brown adipose tissue (BAT; Fig 2D). Moreover, fat was specifically decreased and the weights of other main organs, such as the liver, were not affected after correction for total body weight (Fig 2C, right). The FADD‐D mice exhibited reduced triacylglycerol accumulation according to body composition analysis (Appendix Table S1). There was almost complete absence of s.c. fat and a significant reduction in other fat depots in FADD‐D mice according to MRI analysis (Fig 2E). Histological analysis revealed greater numbers of larger adipocytes in WT than that in FADD‐D mice (Fig 2F). There was an approximate threefold reduction in cell volume of adipocytes from FADD‐D mice, which suggested that decreased cell size partly contributed to the decrease in fat mass. Adipocytes from BAT pads of WT and FADD‐D mice were filled with multilocular lipid droplets that, overall, appeared larger in WT mice (Fig 2F, right). In addition to epididymal fat pads, total triacylglycerol contents in the liver and muscle were also dramatically decreased (Fig 2G). The cholesterol contents were slightly reduced in liver of FADD‐D mice (Fig 2G) and similar in plasma in both groups (Fig 2H). The differences in plasma TG (38% reduction) may be secondary to their differences at tissue levels (Fig 2H).

**Figure 3:**
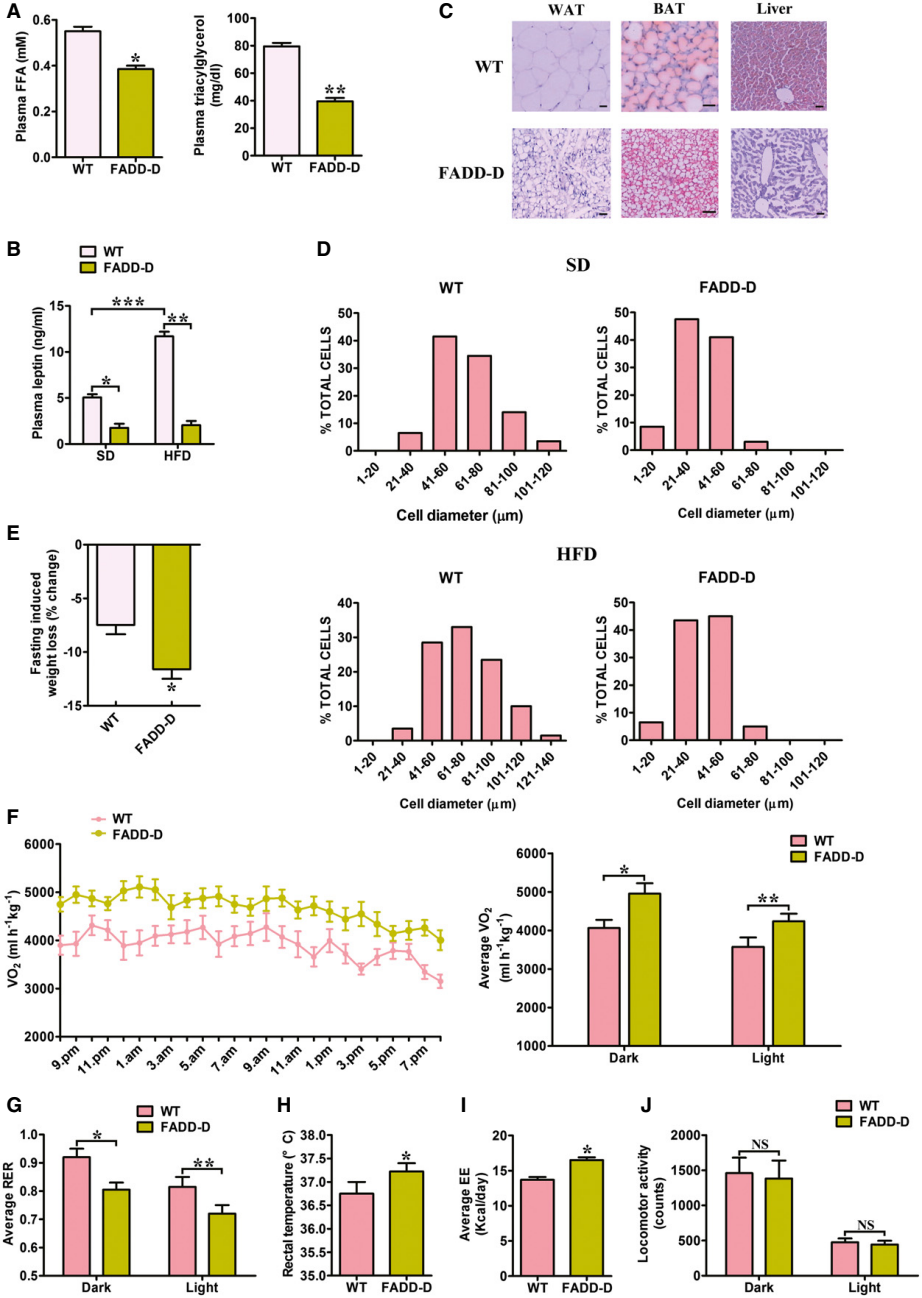
FADD‐D mice prevent from HFD‐induced obesity and show increased oxygen consumption Serum‐free fatty acid (FFA) levels and triacylglycerol levels in WT and FADD‐D mice fed a HFD for 20 days (*n* = 5 for each genotype). Data are expressed as means ± SEM. *P =* *0.0059, **P =* *0.0012 (Student's *t*‐test).Serum levels of leptin in WT and FADD‐D mice (*n* = 4 for each genotype) fed a SD or a HFD. Data are expressed as means ± SEM. *P =* *0.0032, **P =* *0.0003, ***P =* *0.0038 (one‐way ANOVA).Morphology of inguinal WAT, BAT, and liver from WT and FADD‐D mice fed a HFD for 20 days. WAT and BAT were stained with hematoxylin and eosin. Liver tissues were stained with Oil red O to demonstrate lipid accumulation and counterstained with hematoxylin. Scale bars = 50 μm. Shown are typical results from four different fields and three different experiments.Comparison of cell size in WAT of WT and FADD‐D mice maintained on SD and HFD (*n* = 5 for each genotype).FADD‐D mice lost more weight as compared with WT on 20‐h fasting (*n* = 4 for each genotype). Data are expressed as means ± SEM. *P =* *0.0085 (Student's *t*‐test).Whole‐body oxygen consumption rate (VO_2_) during 24 h in WT and FADD‐D mice fed a SD (*n* = 4 for each genotype). Data are expressed as means ± SEM. *P =* *0.0055, **P =* *0.0081 (Student's *t*‐test).Average respiratory exchange ratio (VCO_2_/VO_2_) (RER) for the dark and light period in WT and FADD‐D mice (*n* = 5 for each genotype). Data are expressed as means ± SEM. *P =* *0.0042, **P =* *0.0079 (Student's *t*‐test).Rectal temperature (*n* = 5 for each genotype). Data are expressed as means ± SEM. *P =* *0.0217 (Student's *t*‐test).Average values of energy expenditure (EE) for the 24‐h period in WT and FADD‐D mice (*n* = 5 for each genotype). Data are expressed as means ± SEM. *P =* *0.0054 (Student's *t*‐test).Physical activity for the dark and light period in WT and FADD‐D mice (*n* = 5 for each genotype). Data are expressed as means ± SEM. NS, not statistically significant (Student's *t*‐test).

Serum biochemistry analysis showed that there was no increase in free fatty acids (FFAs) and hypertriglyceridemia in FADD‐D mice after being fed a HFD for 20 days (Fig 3A). As expected, there was a marked decrease in circulating leptin concentrations due to the decreased fat content in adipose tissues (Fig 3B). Histology (Fig 3C) and quantitative analysis of cell size in WAT (Fig 3D) revealed an increase in cell diameter to 71.6 ± 3.2 μm in WT and 41.2 ± 1.7 μm in FADD‐D mice. The reduction of cell volume from the FADD‐D mice was still approximate threefold, the same as that determined previously on a SD. Furthermore, a significant increase in triacylglycerol content in the liver of WT mice on a HFD was not observed in FADD‐D mice according to Oil red O staining (Fig 3C, right), further supporting the non‐lipodystrophic nature of the adipose tissue alterations.

The fact that FADD‐D mice consumed more food than WT mice daily, but gained less weight (Fig 2A), suggests that there are important alterations in energy expenditure and disposition. Indeed, 20‐h‐fasting‐induced weight loss was higher in FADD‐D mice than in WT (Fig 3E), indicating that the decreased adiposity of FADD‐D mice was possibly due to increased energy expenditure. In addition, FADD‐D mice consistently exhibited a 34% increase in oxygen consumption relative to WT littermates (Fig 3F). The respiratory exchange ratio (RER) was decreased in FADD‐D mice during both light phases and dark phases (Fig 3G). The rectal temperatures that were measured at 3:00 pm (basal metabolic state) were markedly higher in the FADD‐D mice relative to the WT mice on a SD (Fig 3H). There were no significant differences, however, in rectal temperatures at other times. In 2‐month‐old FADD‐D mice, energy expenditure (EE) was also significantly increased in comparison with WT mice of the same age (Fig 3I) and changes in EE were not attributable to physical activity (Fig 3J). Thus, the failure to accumulate fat with diet or age in FADD‐D mice seems to stem from a marked increase in metabolic and lipolytic rates.

### FADD‐D mice have enhanced thermogenesis and lipolysis in adipose tissues

**Figure 4:**
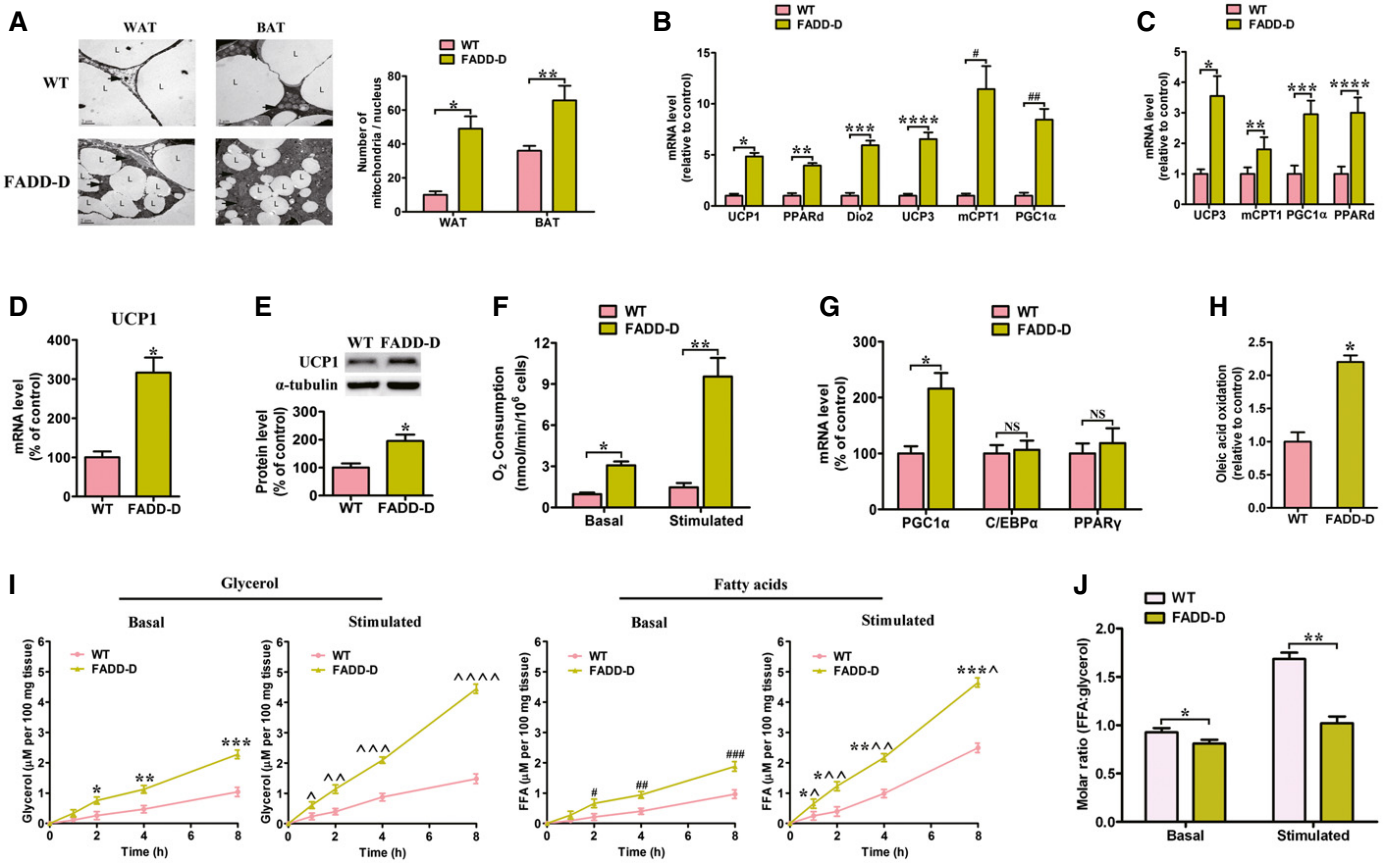
Increased mitochondria, energy expenditure, and lipolysis in adipose tissues of FADD‐D mice Left, transmission electron microscopic analysis of adipocytes from WAT and BAT of WT and FADD‐D mice. Arrowheads indicate mitochondria and lipid droplets (L) (×5,000 magnification). Right, quantification of mitochondria in WAT and BAT of WT and FADD‐D mice, respectively. Data are expressed as mean ± SEM from three independent experiments (*n* = 6 total for each genotype). *P =* *0.0007, **P =* *0.0083 (Student's *t*‐test).Quantitative real‐time PCR for *UCP1*,* Dio2*,* PPARd*,* UCP3*,* CPT1,* and *PGC1‐*α, using RNA from epididymal fat from 10‐week‐old male WT and FADD‐D mice on a SD. Data are expressed as mean ± SEM from three independent experiments (*n* = 6 total for each genotype). *P =* *0.0014, **P =* *0.0025, ***P =* *0.0006, ****P =* *0.0007, ^#^ *P *=* *0.0003, ^##^ *P *=* *0.0008 (Student's *t*‐test).Quantitative real‐time PCR for *UCP3*,* CPT1*,* PPARd* and *PGC1‐*α, using RNA from skeletal muscle from 10‐week‐old male WT and FADD‐D mice on a SD. Data are expressed as mean ± SEM from three independent experiments (*n* = 6 total for each genotype). *P =* *0.0027, **P =* *0.0329, ***P =* *0.0092, ****P =* *0.0085 (Student's *t*‐test).Quantitative real‐time PCR for *UCP1*, using RNA from BAT from 10‐week‐old male WT and FADD‐D mice on a SD. Data are expressed as mean ± SEM from three independent experiments (*n* = 6 total for each genotype). *P =* *0.0018 (Student's *t*‐test).Immunoblotting of UCP1 and tubulin (control) (top) with relative quantification (bottom), using total cell lysates of BAT from 10‐week‐old male WT and FADD‐D mice on a SD. Data are expressed as mean ± SEM from three independent experiments. *P =* *0.0217 (Student's *t*‐test).Oxygen consumption in isolated BAT from 10‐week‐old male WT and FADD‐D mice on a SD (*n* = 4 for each genotype). Data are expressed as mean ± SEM. *P =* *0.0025, **P =* *0.0006 (Student's *t*‐test).Quantitative real‐time PCR for *PGC1*α, *C/EBP*α*,* and *PPAR*γ, using RNA from BAT from 10‐week‐old male WT and FADD‐D mice on a SD. Data are expressed as mean ± SEM from two independent experiments (*n* = 4 total for each genotype). *P =* *0.0113 (Student's *t*‐test). NS, not statistically significant.β‐oxidation analysis of white adipocytes isolated from WT (*n* = 5) and FADD‐D mice (*n* = 4). 1‐^14^C labeled oleic acid was added to the medium containing isolated adipocytes (5 × 10^5^), and ^14^CO_2_ trapped by the filter paper soaked with hyamine hydroxide was measured after a 3‐h incubation by a scintillation counter. Data are expressed as mean ± SEM. *P =* *0.0022 (Student's *t*‐test).Basal and stimulated (+ 100 nM isoproterenol) lipolysis, as measured by glycerol (left) and fatty acids (right) released from explants of epididymal WAT from overnight fasted 10‐week‐old male WT and FADD‐D mice fed a HFD (*n* = 4 for each genotype). Data are expressed as mean ± SEM. *P =* *0.0078, **P =* *0.0065, ***P =* *0.0008, ^*P *=* *0.0133, ^^*P *=* *0.0053, ^^^*P *=* *0.0006, ^^^^*P *=* *0.0001, ^#^ *P *=* *0.0082, ^##^ *P *=* *0.0051, ^###^ *P *=* *0.0009, *^*P *=* *0.0151, *^^*P *=* *0.0019, **^^*P *=* *0.0005, ***^*P *=* *0.0002 (Student's *t*‐test).Molar ratio of FFA to glycerol release from WAT explants (*n* = 4 for each genotype). Data are expressed as mean ± SEM. *P =* *0.0412, **P =* *0.0033 (Student's *t*‐test). Source data are available online for this figure.

Mitochondria play critical roles in lipid metabolism (Zhang *et al*, 2007). Multilocular adipocytes were shown in FADD‐D adipocytes with mitochondria of increased number and size‐phenotypes that were absent in WT adipocytes according to electron micrographs (Fig 4A). The mutant brown adipocytes also had high levels of mitochondria. The cytoplasm of single FADD‐D‐mutant brown adipocyte was densely packed with mitochondria to such an extreme extent that, except for the lipid droplets, no mitochondria‐free area, or other cellular structures could be identified easily (Fig 4A). Consistent with this, the mRNA levels of genes participating in oxidative phosphorylation and energy expenditure were observed to be significantly elevated in FADD‐D adipocytes relative to WT adipocytes, such as peroxisome proliferator‐activated receptor (*PPAR‐*δ), uncoupling protein 1 (*UCP1*), uncoupling protein 3 (*UCP3*), carnitine palmitoyltransferase 1 (*CPT1*), deiodinase‐2 (*Dio2*), and PPAR‐γ co‐activator 1‐α (*PGC1‐*α) (Fig 4B). Expression of *PPAR‐*δ, *PGC1‐*α, *CPT1,* and *UCP3* were also elevated in FADD‐D skeletal muscle (Fig 4C). As FADD‐D mice have enhanced oxidative phosphorylation and reduced WAT, this may partly contribute to the fact that FADD‐D mice are resistant to diet‐induced obesity, which is related to the oxidative phosphorylation pathway (Dressel *et al*, 2003; Luquet *et al*, 2003). Thermogenesis, which is mediated by upregulation of UCP1, is the main result of BAT. UCP1 mRNA and protein levels were markedly elevated in FADD‐D BAT (Fig 4D and E), consistent with increased thermogenesis in these mice. To determine whether FADD phosphorylation affected UCP1 expression and BAT function, WT mice were acutely exposed to the cold. We found that short‐term cold challenge induced a modest elevation in FADD phosphorylation and UCP1 protein levels in BAT (Appendix Fig S4). In addition, oxygen consumption was markedly higher in FADD‐D BAT both at the basal condition and after stimulation with oleate relative to WT mice (Fig 4F). The expression of transcription factors regulating UCP1 gene expression was further examined. We found that expression of PGC1‐α mRNA in BAT was significantly elevated in FADD‐D mice. By contrast, levels of CCAAT/enhancer‐binding protein (c/EBP)‐α and PPAR‐γ mRNA were not changed in BAT of two genotype mice (Fig 4G).

Mitochondria are the main site of β‐oxidation. The primary adipocytes from the mutant mice exhibited more than twofold the rate of oleic acid oxidation found in WT cells (Fig 4H). MK886, a PPAR‐α antagonist (Kehrer *et al*, 2001), decreased fatty acid oxidation to WT levels in FADD‐D WAT explants (Appendix Fig S5A). Basal and stimulated fatty acid and glycerol release from WAT explants of FADD‐D and WT mice were compared to analyze whether the decreased adiposity found in FADD‐D mice was due to increased lipolysis. The rates of fatty acid and glycerol release were markedly elevated in adipose tissue from FADD‐D mice under basal conditions (Fig 4I). Moreover, lipolysis was also enhanced in FADD‐D adipose tissue with isoproterenol treatment, which stimulates lipolysis via upregulating cAMP concentrations (Fig 4I). Of note, the molar ratio of FFA to glycerol release from FADD‐D WAT explants was decreased (Fig 4J). Therefore, the FADD‐D mutation is associated with enhanced lipolysis in adipose tissue. MK886 also decreased lipolysis levels moderately in FADD‐D adipocytes, which were, however, still higher than that in WT adipocytes (Appendix Fig S5B).

### FADD‐D mice have increased adipose cAMP levels

**Figure 5:**
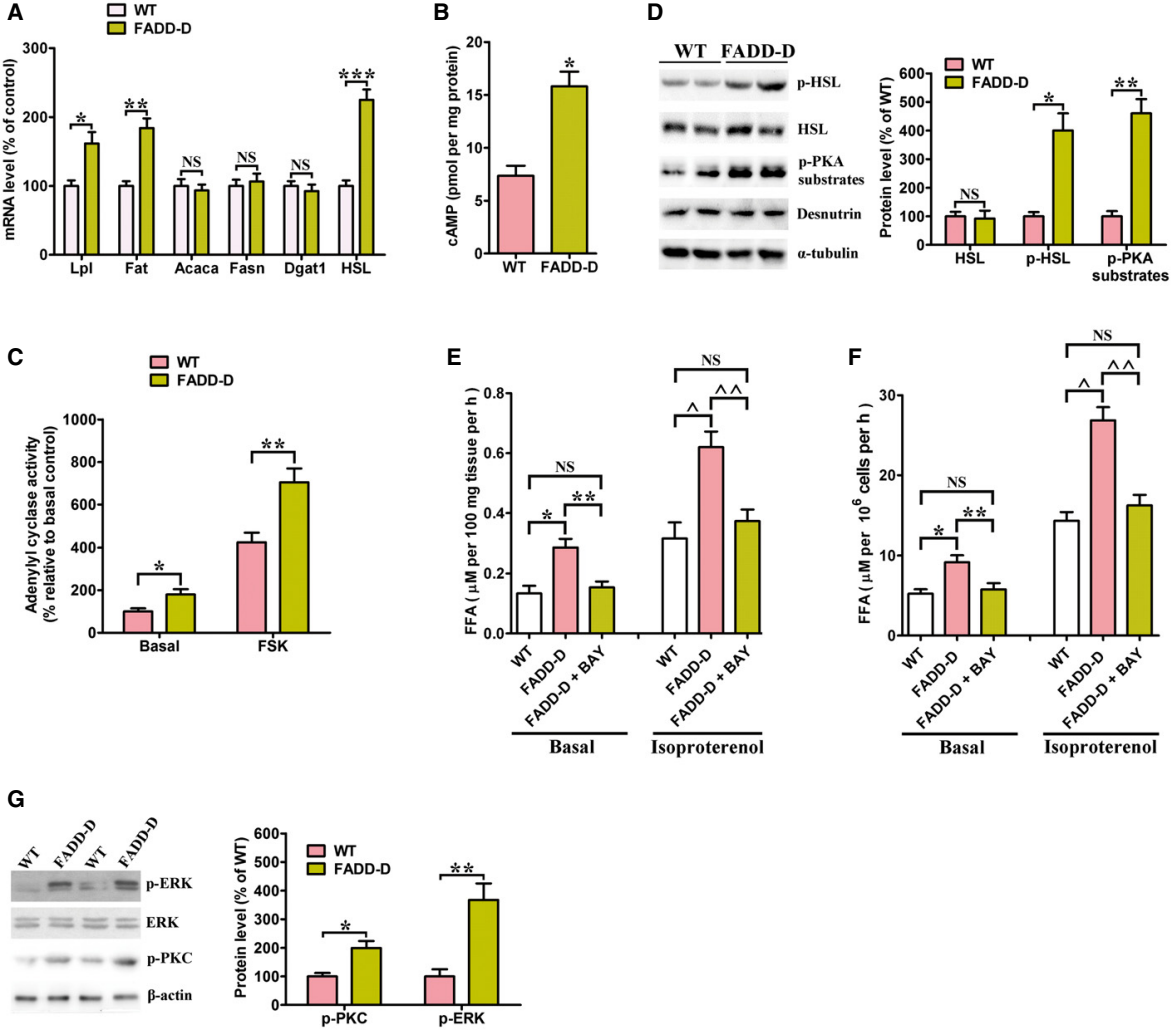
FADD‐D mutation increases lipolysis by increasing cAMP levels and activating HSL Quantitative real‐time PCR for enzymes related to lipid metabolism, using RNA from epididymal fat from 10‐week‐old male WT and FADD‐D mice on a SD. Data are expressed as mean ± SEM from two independent experiments (*n* = 5 total for each genotype). *P =* *0.0162, **P =* *0.0105, ***P =* *0.0063 (Student's *t*‐test). NS, not statistically significant.cAMP abundance in epididymal WAT from 10‐week‐old male WT and FADD‐D mice on a HFD (*n* = 4 for each genotype). Data are expressed as mean ± SEM. *P =* *0.0033 (Student's *t*‐test).Basal and forskolin‐stimulated adenylyl cyclase activity in epididymal WAT from 10‐week‐old male WT and FADD‐D mice on a HFD (*n* = 4 for each genotype). Data are expressed as mean ± SEM. *P =* *0.0177, **P =* *0.0091 (Student's *t*‐test).Immunoblotting of phosphorylated HSL (p‐HSL), HSL, p‐PKA substrate, desnutrin, and tubulin (control) (left) with relative quantification (right). Data are expressed as mean ± SEM from three independent experiments. *P =* *0.0032, **P =* *0.0011 (Student's *t*‐test). NS, not statistically significant.Basal and stimulated (+200 nM isoproterenol) lipolysis in epididymal WAT of 10‐week‐old male WT and FADD‐D mice (*n* = 4 for each genotype) treated with or without 10 μM BAY. Data are expressed as mean ± SEM. *P =* *0.0019, **P =* *00038, ^*P *=* *0.0035, ^^*P *=* *0.0053 (one‐way ANOVA). NS, not statistically significant.Basal and stimulated (+200 nM isoproterenol) lipolysis in isolated adipocytes from 10‐week‐old WT and FADD‐D mice (*n* = 4 for each genotype) treated with or without 10 μM BAY. Data are expressed as mean ± SEM. *P =* *0.0033, **P =* *0.0051, ^*P *=* *0.004, ^^*P *=* *0.0057 (one‐way ANOVA). NS, not statistically significant.Immunoblotting of phosphorylated PKC (p‐PKC), ERK, phosphorylated ERK (p‐ERK), and β‐actin (control) (left) with relative quantification (right). Data are expressed as mean ± SEM from three independent experiments. *P =* *0.0119, **P =* *0.0034 (Student's *t*‐test). Source data are available online for this figure.

The expression of enzymes involved in lipid metabolism in adipose tissue was analyzed to delineate the mechanisms responsible for the increase in lipolysis. FADD‐D mutation promoted an increase in the WAT mRNA level of fatty acid transporter (*Fat*, also known as *CD36*) and lipoprotein lipase (*Lpl*) (Fig 5A), two enzymes responsible for the uptake of circulating fatty acids and TG, respectively. It did not modify the expression of enzymes involved in lipogenesis and TG storage; for example, acetyl‐coenzyme A carboxylase alpha (*Acaca*), fatty acid synthase (*Fasn*), and diacylglycerol O‐acyltransferase homolog 1 (*Dgat1*). However, FADD‐D mutation increased the mRNA level of an enzyme involved in lipolysis, namely hormone‐sensitive lipase (*HSL*). It is known that the classical pathway of lipolysis activation in adipocytes is cAMP dependent (Belfrage *et al*, 1981). As such, we observed an approximate twofold increase in cAMP levels compared with WT mice (Fig 5B) owing to higher adenylyl cyclase activity (Fig 5C). The phosphorylation of HSL was markedly higher while the levels of HSL or desnutrin protein were not changed (Fig 5D). Consistently, expression of phosphorylated substrate for protein kinase A (PKA), which is the kinase that phosphorylates HSL (Watt *et al*, 2006), was elevated in FADD‐D WAT (Fig 5D). These results indicate that the FADD‐D mutation increases lipolysis by upregulating cAMP concentrations, which subsequently activates HSL. In this regard, addition of BAY, a selective HSL inhibitor, decreased lipolysis to WT levels in WAT explants (Fig 5E) and isolated adipocytes from FADD‐D mice (Fig 5F), further supporting that HSL mediates the prolipolytic effect of FADD‐D mutation. In addition, protein kinase C (PKC) has been reported to phosphorylate and activate HSL through the extracellular signal‐regulated protein kinase (ERK) pathway (Jocken & Blaak, 2008). We detected a significant elevation in PKC and ERK phosphorylation levels in WAT of FADD‐D mice (Fig 5G), suggesting that FADD‐D mutation might also promote lipolysis by activating HSL via the activation of PKC and ERK.

### Adipose‐specific FADD knockout mice are resistant to body weight gain induced by a HFD

**Figure 6:**
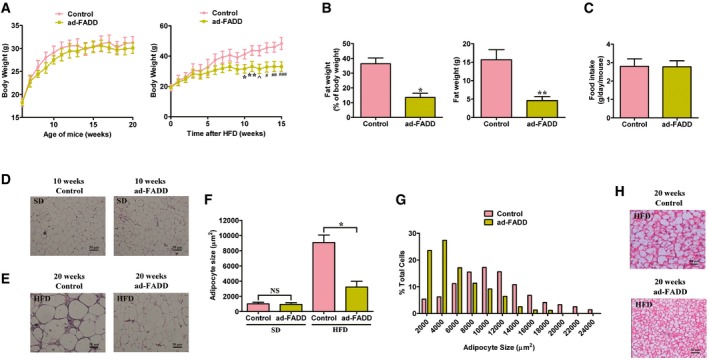
Adipose‐specific FADD knockout mice are resistant to HFD‐induced obesity ATypical growth curves of control and ad‐FADD mice maintained on a SD (left panel) or a HFD (right panel) (*n* = 8 for each genotype). Data are expressed as mean ± SEM. *P =* *0.0435, **P =* *0.0371, ^*P *=* *0.0217, ^#^ *P *=* *0.0236, ^##^ *P *=* *0.0158, ^###^ *P *=* *0.0113 (Student's *t*‐test).BBody composition by NMR to show the fat mass and fat mass ratio in control and ad‐FADD mice after 10 weeks of HFD (*n* = 6 for each genotype). Data are expressed as mean ± SEM. *P =* *0.0027, **P =* *0.0012 (Student's *t*‐test).CFood intake in 15‐week‐old male control and ad‐FADD mice fed a HFD (*n* = 8 for each genotype).D, ERepresentative pictures of hematoxylin‐ and eosin‐stained sections of epididymal WAT from control and ad‐FADD mice at 10 weeks (D) and after 10 weeks of an HFD (E) are shown. Scale bar = 50 μm. Shown are typical results from four different fields and three different experiments.FThe average cross‐sectional area of adipocytes in epididymal WAT is shown in the bar graph (*n* = 6 for each genotype). Data are expressed as mean ± SEM. *P =* *0.002 (Student's *t*‐test). NS, not statistically significant.GThe distribution of adipocyte cross‐sectional area in epididymal WAT of HFD‐fed control or ad‐FADD mice is shown in the bar graph (*n* = 6 for each genotype).HRepresentative pictures of hematoxylin‐ and eosin‐stained sections of brown adipose tissue from HFD‐fed control and ad‐FADD mice are shown. Scale bar = 50 μm. Shown are typical results from four different fields and three different experiments.

Since FADD deficiency exhibits similar effect on PPAR‐α activation as FADD‐D mutation (Fig 1 and Appendix Fig S1), to test whether adipose‐specific FADD deletion produces the same metabolic phenotypes as seen in FADD‐D mice, we generated an adipose‐specific FADD knockout mouse model (ad‐FADD) by crossing loxP‐FADD mice with the aP2‐cre mice, in which CRE expression is under the control of an adipose tissue‐specific aP2 (fatty acid‐binding protein 4, FABP4) promoter. FADD protein level was reduced in WAT and BAT but not in other organs such as lung and liver in ad‐FADD mice (Appendix Fig S6A). FADD mRNA was significantly decreased in WAT from ad‐FADD mice (Appendix Fig S6B). Since aP2 can also be expressed in macrophages, we analyzed aP2 Cre‐mediated deletion of FADD to determine whether it could be detected in this cell type. We found that FADD expression in intraperitoneal‐macrophages from the ad‐FADD mice was not decreased (Appendix Fig S6C). This was consistent with previous studies using the aP2‐cre mouse line, which shows adipocyte restricted expression (Sabio *et al*, 2008; Qi *et al*, 2009; Sugii *et al*, 2009; Li *et al*, 2011). We did not find any apparent abnormalities in the appearance in ad‐FADD mice. Male mice were fed either a SD or HFD to evaluate the specific role of FADD in fat metabolism. Ad‐FADD mice grew at a similar rate to control mice when fed a SD. However, weight gain was observed obviously in control mice after 10 weeks of HFD, while ad‐FADD mice prevented weight gain induced by HFD (Fig 6A). The body fat mass and the ratio of fat to body mass in ad‐FADD mice fed a HFD were decreased according to NMR measurements compared with control mice (Fig 6B) despite similar food intakes (Fig 6C). The epididymal and perirenal WAT of ad‐FADD mice were significantly lighter in weight than those of controls (Appendix Table S2). The weight of other organs such as heart, spleen, and kidney was not significantly different between the two groups. Epididymal WAT histological analysis showed that the adipocytes cell size in ad‐FADD mice was almost the same as that in control mice before HFD (Fig 6D and F). However, the extent of HFD‐induced adipocyte hypertrophy was significantly reduced in ad‐FADD mice compared with control mice (Fig 6E and F). A detailed analysis of the size distribution of the adipocytes revealed that WAT from controls contained a greater number of larger adipocytes than that from ad‐FADD mice (Fig 6G). Lipid particles of adipocytes in BAT from HFD‐fed ad‐FADD mice were apparently smaller compared with those from controls (Fig 6H). To verify whether the decreased body weight gain by FADD deficiency in adipocytes was due to PPAR‐α's activation *in vivo*, we then treated the mice with PPAR‐α antagonist, GW6471, on a HFD for 10 weeks. GW6471 reversed the effect of FADD deletion, and there were no significant differences in body weight and fat weight between two groups of mice (Appendix Fig S7). Furthermore, adipocytes from ad‐FADD mice displayed enhanced fatty acid oxidation and lipolysis as compared to that from control mice. MK886 decreased fatty acid oxidation to WT levels in FADD‐deficient WAT explants (Appendix Fig S8A). Lipolysis was also slightly decreased by MK886 treatment, though the level in ad‐FADD WAT was still higher than that in control mice (Appendix Fig S8B).

### Ad‐FADD mice show increased oxygen consumption with UCP1 upregulation

**Figure 7:**
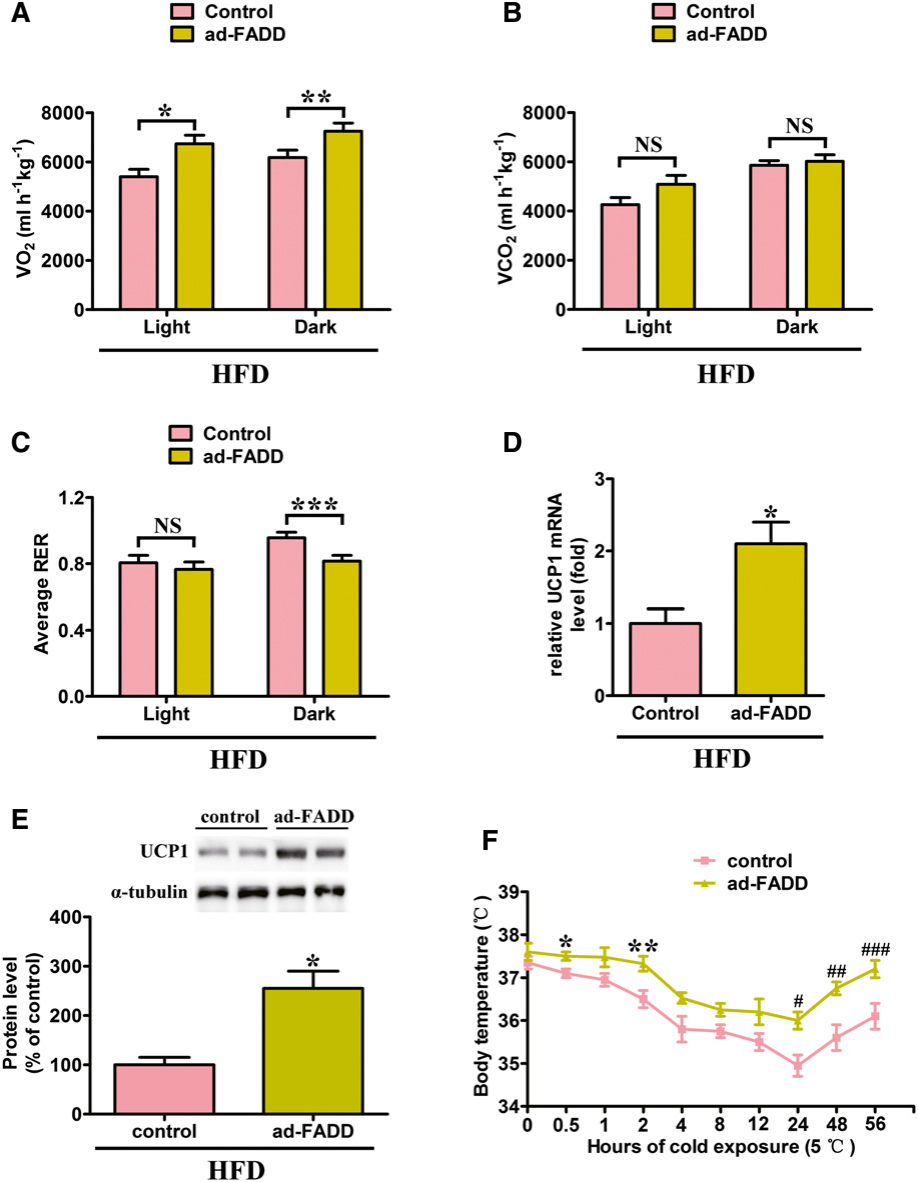
Oxygen consumption and cold‐induced thermogenesis are increased in ad‐FADD mice A–CHFD‐fed control and ad‐FADD mice were housed in a computer‐controlled open‐circuit indirect calorimeter to determine (A) oxygen consumption, (B) carbon dioxide production, and (C) respiratory exchange ratio (RER) during the light (8 am–8 pm) and dark (8 pm–8 am) periods (*n* = 5 for each genotype). Data are expressed as mean ± SEM. *P =* *0.0379, **P =* *0.0453, ***P =* *0.0445 (Student's *t*‐test). NS, not statistically significant.DThe result of real‐time quantitative PCR analysis for *UCP1* in brown adipose tissue from HFD‐fed control and ad‐FADD mice is shown in the bar graph. Data are expressed as mean ± SEM from two independent experiments (*n* = 4 total for each genotype). *P =* *0.0172 (Student's *t*‐test).EImmunoblot of UCP1 and tubulin (control) (top) in BAT from control and ad‐FADD mice with relative quantification (bottom). Data are expressed as mean ± SEM from three independent experiments. *P =* *0.0053 (Student's *t*‐test).FMice were initially maintained at room temperature and then transferred to cold incubator (5°C). Rectal body temperature was recorded at the indicated time points using thermoprobe (*n* = 6 for each genotype). Results are means ± SEM. *P =* *0.0466, **P =* *0.0357, ^#^ *P *=* *0.0229, ^##^ *P *=* *0.0138, ^###^ *P *=* *0.0206 (Student's *t*‐test). Source data are available online for this figure.

Oxygen consumption was significantly increased in HFD‐fed ad‐FADD mice in both the light and dark periods (Fig 7A). Carbon dioxide production was slightly increased in ad‐FADD mice, but the difference was not statistically significant (Fig 7B). The respiratory exchange ratio was significantly lower in ad‐FADD mice during the dark period when mice were active, but there was no difference during the light period (Fig 7C). The expression of UCP1 was significantly upregulated in HFD‐fed FADD‐deficient BAT compared with controls (Fig 7D and E), suggesting that FADD deletion in adipocytes increased energy expenditure using lipid at least partly mediated by upregulation of UCP1 in BAT. To determine whether FADD ablation affected BAT function and thermogenesis, ad‐FADD and control mice were acutely exposed to the cold. In contrast to control littermates, ad‐FADD mice displayed better cold resistance (Fig 7F), indicating higher thermogenesis.

### Adipocyte‐specific FADD deletion improves HFD‐induced insulin resistance and glucose intolerance

**Figure 8:**
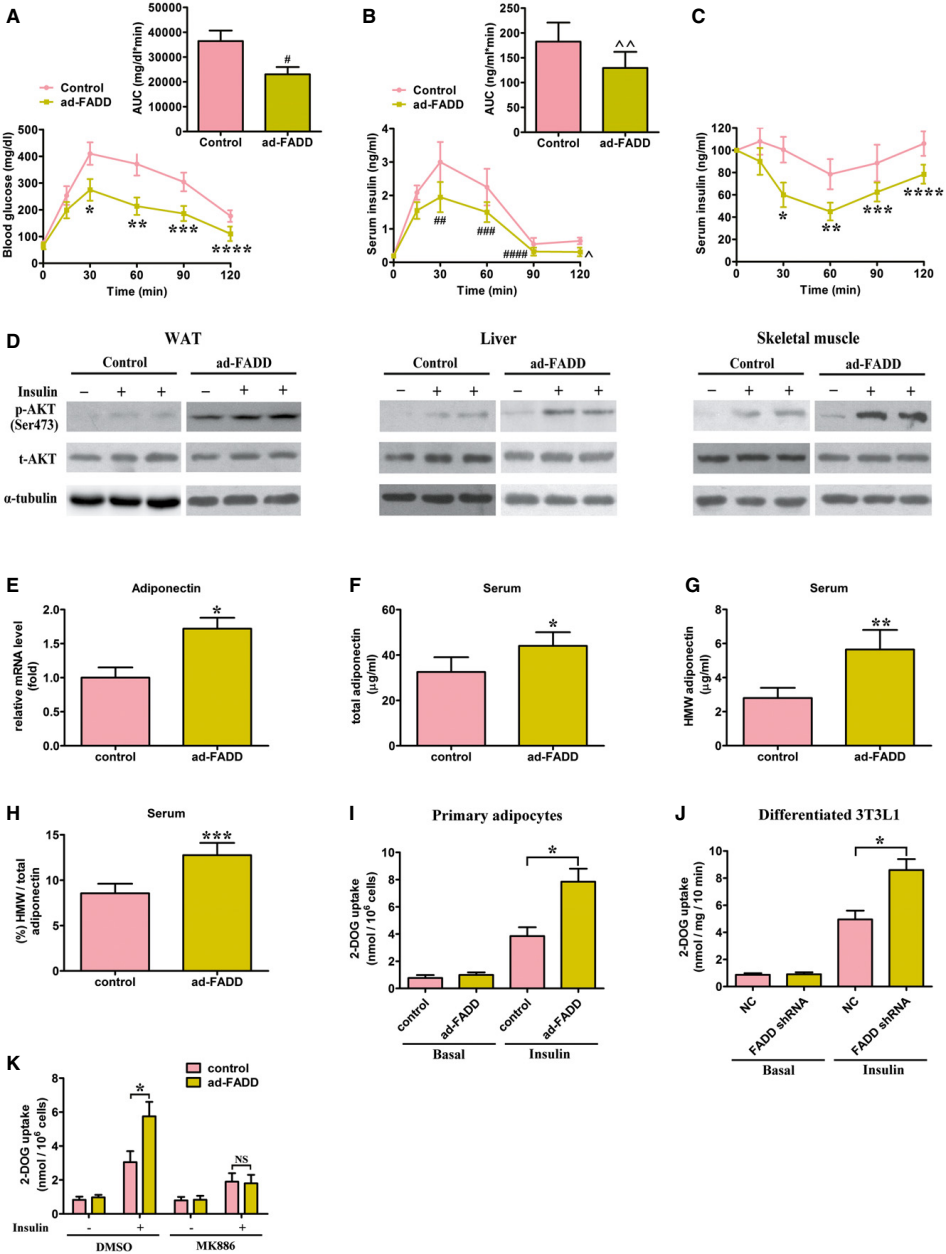
FADD disruption in adipocytes improves HFD‐induced glucose intolerance and insulin resistance A, BBlood glucose levels and serum insulin levels in 2‐h GTT 12 weeks after HFD. Inset graphs in (A) and (B) depict the respective analysis of the area under the curve (AUC). Data shown are from one experiment (*n* = 6 for each genotype), representative of a total of two independent experiments. Results are means ± SEM. *P =* *0.0287, **P =* *0.0173, ***P =* *0.0332, ****P =* *0.0453, ^#^ *P *=* *0.0077, ^##^ *P *=* *0.0344, ^###^ *P *=* *0.0412, ^####^ *P *=* *0.0495, ^*P *=* *0.0435, ^^*P *=* *0.0304 (Student's *t*‐test).CITT 12 weeks after HFD. Data shown are from one experiment (*n* = 6 for each genotype), representative of a total of two independent experiments. Results are means ± SEM. *P =* *0.0127, **P =* *0.0232, ***P =* *0.0373, ****P =* *0.0385 (Student's *t*‐test).DInsulin‐stimulated Akt phosphorylation (Ser473) in WAT, liver, and skeletal muscle of control and ad‐FADD mice. The experiments were performed in triplicate.EAdiponectin mRNA was measured in isolated epididymal adipocytes by RT–PCR. Data are expressed as mean ± SEM from three independent experiments. *P =* *0.0044 (Student's *t*‐test).F–HTotal adiponectin levels (F), HMW adiponectin (G), and the ratio of HMW to total adiponectin (H). *n* = 5 for each genotype. Data are expressed as mean ± SEM. *P =* *0.0217, **P =* *0.0116, ***P =* *0.0175 (Student's *t*‐test).IBasal and insulin‐stimulated glucose uptake in control and FADD‐deficient primary isolated adipocytes. Data shown are from one experiment (*n* = 4 for each genotype), representative of a total of two independent experiments. Results are means ± SEM. *P =* *0.0073 (Student's *t*‐test).JBasal and insulin‐stimulated glucose uptake in 3T3L1 cells differentiated to adipocytes and infected with lentivirus containing FADD shRNA or scrambled shRNA control. Data are expressed as mean ± SEM from three independent experiments. *P =* *0.0092 (Student's *t*‐test).KBasal and insulin‐stimulated glucose uptake in control and FADD‐deficient primary isolated adipocytes treated with DMSO or 5 μM MK886. Data are expressed as mean ± SEM from four independent experiments. *P =* *0.0135 (one‐way ANOVA). NS, not statistically significant.

GTTs and ITTs were performed to investigate the role of adipocyte‐specific FADD deletion in insulin resistance induced by obesity. There were no marked differences in ITT and GTT between ad‐FADD and control mice when they were fed a SD. GTT, however, showed that after 12 weeks of HFD feeding, ad‐FADD mice exhibited markedly decreased blood glucose relative to control mice after glucose loading (Fig 8A). In addition, ad‐FADD mice had markedly decreased serum insulin during GTT (Fig 8B). There was a significant improvement in insulin sensitivity during ITT by adipose‐specific FADD deletion (Fig 8C). In addition, fasted serum insulin, fasted glucose, and fed glucose levels were markedly reduced in ad‐FADD mice compared with control mice (Appendix Table S3). Also, ad‐FADD mice exhibited decreased fasted serum FFA and triglyceride levels, which was consistent with improved insulin sensitivity and glucose tolerance in these mice (Appendix Table S3). Furthermore, adipocyte‐specific FADD deletion improved insulin signaling pathways in skeletal muscle, liver, and WAT, as shown by elevated phosphorylation of Akt (Fig 8D). Taken together, our results suggest that adipocyte‐specific FADD deletion improves HFD‐induced insulin resistance and glucose tolerance.

Adiponectin is a critical insulin sensitizer that is specifically secreted from adipose tissues and is downregulated by high‐fat diets (Kadowaki *et al*, 2006). Indeed, fat‐specific FADD deletion ameliorated the marked reduction in adiponectin mRNA level as shown in isolated epididymal adipocytes from WT mice on a HFD (Fig 8E). After fed a 12‐week HFD, ad‐FADD mice exhibited increased serum total adiponectin (Fig 8F), the high molecular weight (HMW) form of adiponectin (Fig 8G), and the ratio of HMW to total adiponectin (Fig 8H). We next examined the effects of FADD on glucose uptake stimulated by insulin in adipocytes. Primary adipocytes isolated from ad‐FADD mice showed a twofold increase in insulin‐stimulated glucose uptake compared with that from control mice (Fig 8I). Likewise, shRNA knockdown of FADD increased insulin‐stimulated glucose uptake in 3T3L1 cells (Fig 8J). However, MK886 rescued the increase in glucose uptake in FADD‐deficient adipocytes (Fig 8K). These results indicate that the ability of FADD to regulate glucose uptake is PPAR‐α dependent.

### Adipose tissue of ad‐FADD mice on a HFD has reduced macrophage infiltration

**Figure 9:**
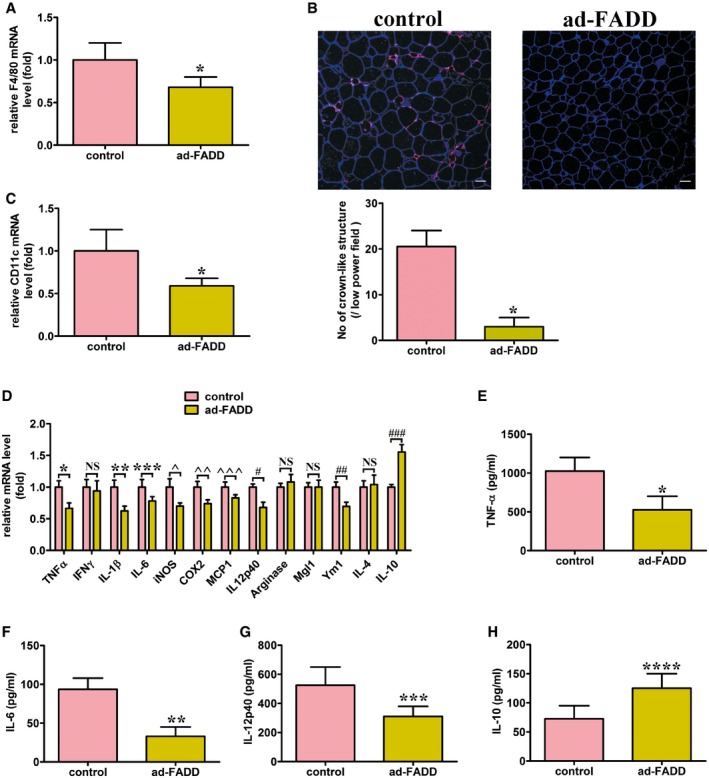
Decreased adipose tissue inflammation in ad‐FADD mice ARelative mRNA levels of the macrophage marker F4/80 in SVCs isolated from epididymal adipose tissue. Data are expressed as mean ± SEM from four independent experiments. *P =* *0.0279 (Student's *t*‐test).BF4/80 immunostaining in epi‐WAT. Scale bars = 50 μm. Shown are typical results from four different fields and three different experiments. Data are expressed as mean ± SEM. *P =* *0.0003 (Student's *t*‐test).CRelative mRNA levels of the proinflammatory M1‐like macrophage marker CD11c in SVCs isolated from epididymal adipose tissue. Data are expressed as mean ± SEM from five independent experiments. *P =* *0.0221 (Student's *t*‐test).DRelative mRNA levels of inflammatory and anti‐inflammatory cytokines in Epi‐WAT. Data are expressed as mean ± SEM from four independent experiments. *P =* *0.0201, **P =* *0.0159, ***P =* *0.0263, ^*P *=* *0.0227, ^^*P *=* *0.0215, ^^^*P *=* *0.0312, ^#^ *P *=* *0.0231, ^##^ *P *=* *0.0255, ^###^ *P *=* *0.0094 (Student's *t*‐test). NS, not statistically significant.E–HSerum levels of TNF‐α (E), IL‐6 (F), IL‐12p40 (G), and IL‐10 (H). *n* = 5 for each genotype. Data are expressed as mean ± SEM. *P =* *0.0077, **P =* *0.0052, ***P =* *0.0114, ****P =* *0.0127 (Student's *t*‐test).

Chronic inflammation is reported as a common feature in the adipose tissue of obese subjects (Nishimura *et al*, 2007). We assessed macrophage infiltration into the epididymal fat pad, which is related to visceral adiposity, to evaluate the inflammatory consequences of FADD deficiency in adipocytes when mice were fed a HFD. Assessment of F4/80, markers for activated macrophages, in isolated macrophage‐containing stromal vascular cells (SVCs) of the epididymal adipose fat pad indicated that FADD deficiency in adipocytes could prevent the increase in F4/80 level shown in WT mice fed a HFD for 12 weeks (Fig 9A). The macrophage aggregation surrounding adipocytes, often referred to as a crown‐like structure (Weisberg *et al*, 2003), was significantly decreased in WAT from HFD‐fed ad‐FADD mice compared with controls (Fig 9B). The majority of the increase in macrophages with obesity is due to M1‐like macrophages (triply positive, CD11b^+^, CD11c^+^, and F4/80^+^ cells), which overexpress proinflammatory cytokines relative to the M2‐like macrophages (CD11c^−^) (Hevener *et al*, 2007). As shown in Fig 9C, there was a significant reduction in CD11c^+^ macrophages in ad‐FADD mice relative to the controls. Most proinflammatory genes, including *TNF*α, *IL‐1*β, *IL‐6*,* iNOS*,* COX2*,* MCP1*, and *IL12p40*, were decreased in the ad‐FADD mice, while expression of the anti‐inflammatory gene *IL‐10* was increased (Fig 9D). Since adipose tissue is proposed to be a major source of circulating levels of cytokines, we measured their levels in the circulation. Whereas circulating TNFα, IL‐6, and IL12p40 levels were significantly reduced after 12 weeks of HFD (Fig 9E–G), IL‐10 levels were approximately 60% higher in the ad‐FADD mice (Fig 9H). It therefore suggests that FADD deficiency in adipocytes can decrease local inflammatory consequences in fat that are usually rendered with a HFD, leading to reduced macrophage infiltration.

### FADD‐D mutation ameliorates obesity in *ob*/*ob* mice

**Figure 10:**
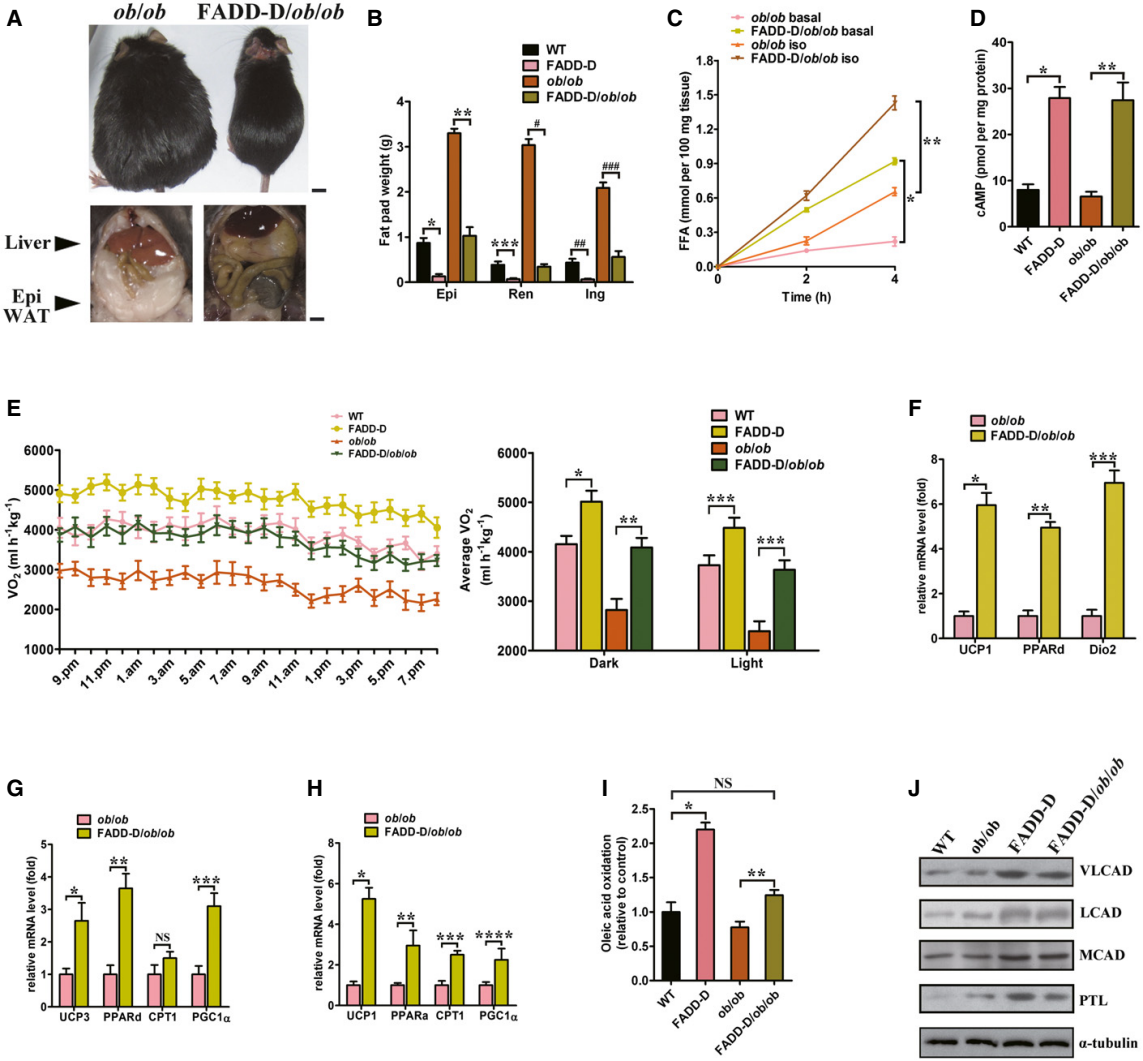
FADD‐D mutation prevents obesity in *ob*/*ob* leptin‐deficient mice Top, representative photographs of 20‐week‐old male *ob*/*ob* and FADD‐D/*ob*/*ob* mice fed a SD. Scale bar = 6 mm. Bottom, representative photographs of their livers and epididymal WAT. Scale bar = 4 mm.Comparison of weights of WAT depots from WT, FADD‐D, *ob*/*ob* and FADD‐D/*ob*/*ob* mice. *n* = 5 for each genotype. Data are expressed as mean ± SEM. *P =* *0.0007, **P =* *0.0022, ***P =* *0.0011, ^#^ *P *=* *0.0009, ^##^ *P *=* *0.0003, ^###^ *P *=* *0.0027 (one‐way ANOVA).Basal and stimulated lipolysis measured by fatty acid release from explants of epididymal WAT in 10‐week‐old male *ob*/*ob* and FADD‐D/*ob*/*ob* mice fed a HFD. *n* = 4 for each genotype. Data are expressed as mean ± SEM. *P =* *0.0003, **P =* *0.001 (one‐way ANOVA).cAMP level in WAT of 10‐week‐old male WT, FADD‐D, *ob*/*ob* and FADD‐D/*ob*/*ob* mice fed a HFD. *n* = 4 for each genotype. Data are expressed as mean ± SEM. *P =* *0.0017, **P =* *0.001 (one‐way ANOVA).Whole‐body oxygen consumption rate (VO_2_) during 24 hr in WT, FADD‐D, *ob*/*ob*, and FADD‐D/*ob*/*ob* mice fed a SD. *n* = 4 for each genotype. Data are expressed as mean ± SEM. *P =* *0.0053, **P =* *0.0025, ***P =* *0.0062, ****P =* *0.0011 (one‐way ANOVA).Quantitative real‐time PCR for *Dio2*,* PPARd,* and *UCP1*, using RNA from epididymal fat from 20‐week‐old male *ob*/*ob* and FADD‐D/*ob*/*ob* mice fed a SD. Data are expressed as mean ± SEM from four independent experiments. *P =* *0.0009, **P =* *0.0015, ***P =* *0.0003 (Student's *t*‐test).Quantitative real‐time PCR for *UCP3, PGC‐1*α, *PPARd,* and *CPT1*, using RNA from skeletal muscle from 20‐week‐old male *ob*/*ob* and FADD‐D/*ob*/*ob* mice fed a SD. Data are expressed as mean ± SEM from four independent experiments. *P =* *0.0105, **P =* *0.0033, ***P =* *0.0058 (Student's *t*‐test). NS, not statistically significant.Quantitative real‐time PCR for *UCP1*,* PPARa*,* CPT1,* and *PGC‐1*α, using RNA from brown adipose fat from 20‐week‐old male *ob*/*ob* and FADD‐D/*ob*/*ob* mice fed a SD. Data are expressed as mean ± SEM from four independent experiments. *P =* *0.0012, **P =* *0.0129, ***P =* *0.0103, ****P =* *0.0207 (Student's *t*‐test).Oxidation of [1‐^14^C] oleic acid to ^14^CO_2_ by adipocytes isolated from WT, FADD‐D, *ob*/*ob*, and FADD‐D/*ob*/*ob* mice. Data shown are from one experiment (*n* = 3 for each genotype), representative of a total of two independent experiments. Results are means ± SEM. *P =* *0.0031, **P =* *0.0075 (one‐way ANOVA). NS, not statistically significant.Immunoblot of VLCAD, LCAD, MCAD, PTL, and tubulin (control), using protein extracts from epididymal fat from 15‐week‐old male WT, FADD‐D, *ob*/*ob* and FADD‐D/*ob*/*ob* mice fed a SD. Data shown are representative of three independent experiments having similar results. Source data are available online for this figure.

FADD‐D was introduced into *ob*/*ob* mice to create FADD‐D/*ob*/*ob* double‐mutant mice to determine whether the FADD‐D mutation could prevent genetic obesity as caused by leptin deficiency (Appendix Fig S9A). When fed a SD, FADD‐D/*ob*/*ob* mice gained much less weight than the *ob*/*ob* mice (Fig 10A and Appendix Fig S9B). Differences in body weight were observed obviously as early as 6 weeks of age and these differences became more significant with age (Appendix Fig S9B). Surprisingly, the differences in body weight could not be due to a reduction in food consumption because food intake was increased in the FADD‐D/*ob*/*ob* mice relative to the *ob*/*ob* mice (Appendix Fig S9B, right). FADD‐D/*ob*/*ob* mice had decreased adiposity with a significant reduction in WAT depots weight relative to the *ob*/*ob* mice (Fig 10B). The weights of other organs were similar between the two genotypes of mice. The body and carcass weights of FADD‐D or FADD‐D/*ob*/*ob* mice at 25 weeks of age were decreased as compared to those of WT or *ob*/*ob* mice according to carcass analysis. However, the percentage of lean tissue mass and water were increased (Appendix Fig S9C), reflecting the substantially leaner phenotype endowed by FADD‐D mutation. Under both basal and stimulated conditions, there was markedly higher lipolysis in FADD‐D/*ob*/*ob* mice relative to *ob*/*ob* mice (Fig 10C) and a fourfold increase in cAMP levels (Fig 10D). Overall, these data suggest that, even in leptin deficiency‐induced genetic obesity, FADD‐D mutation can modulate lipolysis and adiposity through the marked influence on cAMP levels.

Despite being significantly leaner, the FADD‐D/*ob*/*ob* double‐mutant mice consumed more food than the *ob*/*ob* mice (Appendix Fig S9B). Compared to the WT and *ob*/*ob* mice, total oxygen consumption was increased in FADD‐D or FADD‐D/*ob*/*ob* mice, respectively (Fig 10E). These differences, however, were not due to alterations in ambulatory activity. Consistent with this, in WAT from FADD‐D/*ob*/*ob* mice, we detected a sixfold increase in mRNA levels of *UCP1* and upregulation of other genes that participate in oxidative metabolism, such as fivefold and sevenfold increases in mRNA levels of *PPAR‐*δ and *Dio2* as compared to *ob*/*ob* littermates, respectively (Fig 10F). Brown adipose tissue and muscle play a key role in energy expenditure in mice (Muoio & Newgard, 2006). Increases in *UCP3, PGC1‐a, CPT1,* and *PPAR‐*δ gene expression were observed in FADD‐D/*ob*/*ob* mice skeletal muscle relative to *ob*/*ob* mice (Fig 10G). Most of the genes that participate in energy metabolism, including *UCP1*,* PPAR‐*α, *CPT1*α, and *PGC1‐*α, were upregulated in the BAT of FADD‐D/*ob*/*ob* mice (Fig 10H). Protein level of UCP1 was also elevated in BAT of FADD‐D/*ob*/*ob* mice (Appendix Fig S9D). Fatty acid oxidation was markedly lower in *ob*/*ob* adipocytes than in WT adipocytes, which was restored, however, to WT levels in FADD‐D/*ob*/*ob* adipocytes (Fig 10I). Proteins participating in mitochondrial and peroxisomal fatty acid oxidation systems were also found to be strongly elevated in both FADD‐D and FADD‐D/*ob*/*ob* adipocytes relative to WT and *ob*/*ob*, including peroxisomal 3‐ketoacyl‐CoA thiolase A (PTL), VLCAD, LCAD, and MCAD (Fig 10J), indicating transcriptional activation of PPAR‐α‐regulated genes involved in fatty acid oxidation. In our study, there was a reduction in the molar ratio of FFA to glycerol release from FADD‐D WAT explants in relation to WT mice (Fig 4J). Increased use of FFA in adipocytes is therefore indicated by this alteration in the molar ratio of FFA to glycerol in FADD‐D mice, which is consistent with enhanced fatty acid oxidation determined previously in adipose tissue. This offers a potential explanation for not only increased energy expenditure but also decreased FFA in the circulation even though there was higher lipolysis in FADD‐D mice.

### FADD‐D mutation reverses hyperglycemia and reduces hepatic steatosis of *ob*/*ob* mice

Alterations in insulin and glucose homeostasis are often the result of changes in adiposity. Leptin‐deficient *ob*/*ob* mice have diabetes and are obese, and they also exhibit hyperinsulinemia and hyperglycemia levels (Coleman, 1978; Friedman & Halaas, 1998). Higher blood glucose levels were found in both fed and fasted *ob*/*ob* mice compared to WT, FADD‐D, and FADD‐D/*ob*/*ob* mice at 15 weeks of age (Appendix Fig S10A). Blood glucose content was reduced in fasted FADD‐D/*ob*/*ob* mice, which indicates that the double‐mutant mice are prevented from diabetes. Insulin sensitivity and glucose tolerance were increased in FADD‐D/*ob*/*ob* mice versus *ob*/*ob* mice (Appendix Fig S10B). Obesity in the *ob*/*ob* mice is usually related to severe hepatic steatosis (Coleman, 1978). Gross inspection revealed that the hepatomegaly and steatosis of *ob*/*ob* mice were normalized in FADD‐D/*ob*/*ob* double mutants. Histological sections of liver from *ob*/*ob* mice showed large lipid‐filled vacuoles, whereas FADD‐D/*ob*/*ob* mice showed little or no vacuolation and were indistinguishable from those of WT mice (Appendix Fig S10C). In contrast to the substantially increased liver triglyceride in *ob*/*ob* mice, triglycerides in FADD‐D/*ob*/*ob* mice were reduced to levels comparable to WT controls (Appendix Fig S10D).

Serum FFA levels were lower despite increased lipolysis in FADD‐D and FADD‐D/*ob*/*ob* mice on a SD (Appendix Fig S11A). FADD‐D and double‐mutant mice also had lower serum triglycerides (Appendix Fig S11B). In our study, there was increased triacylglycerol clearance in FADD‐D mice receiving oral feeding of lipids (Appendix Fig S11C). However, there was no apparent difference in serum triacylglycerol levels when the pharmacologic agent WR1339, a detergent inhibiting lipoprotein removal from the circulation, was firstly injected (Appendix Fig S11D). Lipoprotein lipase (LPL) mRNA level in livers from FADD‐D mice was more than twofold higher than that from WT mice (Appendix Fig S11E). Overall, our results indicate that there may be higher fatty acid uptake by the liver in FADD‐D mice.

## Discussion

FADD serves as a critical adaptor in death receptor signaling. Further studies have revealed its role of “proliferation–apoptosis coupler” regulated by its phosphorylation (Hua *et al*, 2003). FADD‐D mice showed apparent growth retardation (Cheng *et al*, 2014) and were obviously leaner than WT littermates. Our previous proteomics analysis of FADD‐deficient and FADD‐D‐mutant cells identified an enriched cluster of changed proteins involved in cellular metabolic process, including lipid metabolism, fatty acid, and metabolism (Zhuang *et al*, 2013a,b). Therefore, we speculate that FADD probably has a critical role in lipid metabolism. However, the molecular mechanisms remain largely unknown. Here, we present a molecular model for how Ser‐191 phosphorylation regulates the novel function of FADD. FADD is a negative regulator for the activation of PPAR‐α, an important transcriptional factor involved in lipid metabolism. The mutant mimicking constitutive phosphorylation (FADD‐D) has a decreased interaction with RIP140, a corepressor for PPAR‐α, which then results in an impaired interaction between PPAR‐α and RIP140 and finally leads to RIP140 derepression and activation of PPAR‐α. These data suggest that Ser‐191 phosphorylation abolishes the novel “adaptor” function of FADD. A schematic framework of PPAR‐α regulation by FADD is presented in Appendix Fig S1J and Appendix Fig S12. We propose that FADD acts to negatively regulate PPAR‐α transcriptional activation and signaling, ultimately controlling fatty acids oxidation and fat accumulation. Upon phosphorylation at Ser‐191, FADD loses contact with RIP140. PPAR‐α then dissociates from RIP140. The escape of PPAR‐α from repression by RIP140 accelerates PPAR‐α‐mediated transcription of genes involved in lipid metabolism and enhances fat utilization. Several reports have demonstrated that PPAR‐α‐null mice were associated with obesity (Costet *et al*, 1998) and exhibited increased adiposity and adipocyte hypertrophy (Knauf *et al*, 2006). The corepressor protein RIP140‐deficient mice exhibited resistance to HFD‐induced obesity and were lean (Leonardsson *et al*, 2004). Thus, here PPAR‐α activating in mice by FADD‐D mutation or FADD deficiency might lead to lean phenotype and reduced adiposity reasonably. Previous studies have found that both total body Fas‐deficient mice and adipocyte‐specific Fas‐knockout mice were significantly protected from HFD‐induced changes in adipose tissue and glucose homeostasis (Wueest *et al*, 2010). Here, in the current study, we show that, as a classical adaptor in the Fas‐mediated pathway, FADD has an important role in modulating fatty acids oxidation by regulating PPAR‐α's activation in adipose tissue. As a result, FADD‐D mutation or adipose‐specific FADD disruption in mice prevents obesity induced by leptin deficiency or by a HFD feeding. It is possible that FAS and FADD work together to play a critical role in obesity and obesity‐associated pathologies.

There was a significant decrease in adipose mass in mice with the FADD‐D mutation. Our study revealed that this decrease resulted from a reduction in cell volume but not cell numbers. FADD‐D adipocytes suffered severe morphological abnormalities, such as decreased cell size, markedly increased cytoplasmic volume with more mitochondria, and a significantly high percentage of cells containing multilocular lipid droplets. We hypothesized that the reduction in the volume of adipocytes was caused by increased mobilization of intracellular fat because the intracellular lipids content determines adipocyte volume. As predicted, triacylglycerol lipolysis in WAT from FADD‐D mice was significantly stimulated. Adipocyte lipolysis is a complex process that is precisely controlled through integration of multiple and diverse hormonal and biochemical signals. Breakdown of this regulation may contribute to development of obesity and associated pathologies. In adipocytes isolated from FADD‐D mice and in explants of FADD‐D adipose tissue, both the basal and the isoproterenol‐stimulated lipolysis were elevated. cAMP is an important second messenger in the signaling pathways that mobilize fat stores (Holm *et al*, 2000). We found that cAMP levels were markedly increased in FADD‐D and FADD‐D/*ob*/*ob* double‐mutant mice. This increase in cAMP levels activates PKA, which subsequently phosphorylates and activates HSL. FADD‐D mice were shown to exhibit elevated cAMP levels and increased expression of phosphorylated‐PKA substrates involving HSL, which suggests that FADD‐D mutation‐induced fat utilizing is likely mediated through the cAMP‐PKA‐HSL signaling pathway. However, the exact mechanisms for how FADD regulates the levels of cAMP are not yet clear and warrant further investigation. In addition, a novel signaling pathway by which HSL activity may be stimulated by protein kinase C (PKC) via extracellular signal‐regulated kinase (ERK) has been demonstrated (Donsmark *et al*, 2003, 2004). Previous study found that FADD could modulate PKC dephosphorylation, stability, and signaling termination (Cheng *et al*, 2012). Consistently, in the current study, we found that FADD‐D mice exhibited increased levels of phosphorylated PKC and ERK. Thus, activation of the PKC‐ERK pathway by FADD‐D mutation appears to be able to regulate adipocyte lipolysis by phosphorylating HSL and increasing the activity of HSL. On the other hand, it has also been reported that PPAR‐α activators stimulated lipolysis in WAT (Guzman *et al*, 2004). Consistently, in our current study, inhibition of PPAR‐α function partially inhibited lipolysis. These data strongly suggest that FADD‐D mutation may enhance lipolysis by different signaling mechanisms.

Since FADD‐D mice showed apparent growth retardation (Cheng *et al*, 2014), the body weight difference may just not be a direct result of metabolic change; it may also possibly be due to other changes during growth retardation. In this regard, further animal models and experiments need to be investigated to illustrate this issue. Therefore, to completely understand the role of FADD in metabolism, it is necessary to understand the tissue‐specific FADD contribution to the phenotype of the whole organism. Consistently, we demonstrated that FADD deletion in adipocyte alleviated diet‐induced obesity and glucose intolerance in mice. FADD deletion reduced fat mass and increased the expression of UCP1 in BAT and oxygen consumption, all of which are supposed to be responsible for body weight reduction and better glucose tolerance in HFD‐fed ad‐FADD mice. The size of adipocytes in FADD‐deficient WAT was reduced. It is generally accepted that better glucose tolerance is associated with smaller adipocyte size and conversely that hypertrophied adipocytes are strongly linked to insulin resistance (Hotamisligil, 2006). Adipocyte‐specific FADD deletion activated insulin signaling pathways in skeletal muscle, liver, and WAT, thereby contributing to the improvement of HFD‐induced whole‐body glucose intolerance. Furthermore, ad‐FADD mice demonstrated decreased adipose tissue macrophages content and this was accompanied by decreased expression of proinflammatory genes in the adipose tissue. Chronic tissue inflammation has been reported to be a main cause of insulin resistance. The underlying mechanism might be due to macrophage‐mediated proinflammatory effects (Hotamisligil & Erbay, 2008; Donath & Shoelson, 2011). Therefore, decreased tissue inflammation in ad‐FADD mice might be responsible for the improved insulin signaling in WAT. Serum total adiponectin levels were higher in ad‐FADD mice. Adiponectin has been reported to improve insulin signaling in the liver and skeletal muscle (Yamauchi *et al*, 2002). Thus, the findings in the current study that ad‐FADD mice exhibited improved insulin sensitivity in muscle and liver can be reasonably explained. In fact, compared to total adiponectin levels, HMW adiponectin levels and the ratio of HMW to total adiponectin are more important in the prediction of insulin resistance and the development of metabolic syndrome (Matsushita *et al*, 2006). In addition, serum FFAs and triglycerides were decreased in ad‐FADD mice. This is consistent with previous studies revealing that triglycerides and FFAs could induce insulin resistance by impairing insulin signaling in muscle and liver (Hulver & Dohm, 2004; Leroyer *et al*, 2006). FADD deficiency improves metabolic functions. Therefore, compounds inhibiting FADD activity in adipose tissue might have therapeutic potential for preventing insulin resistance and obesity. In addition, FADD deletion in adipocytes may enhance lipid combustion. Ad‐FADD mice consumed more oxygen with a lower respiratory exchange ratio and showed reduced lipid content in BAT, which may be explained, at least in part, by the upregulation of the expression of UCP1, a known PPAR‐α target gene (Villarroya *et al*, 2007). It has been reported that activation of PPAR‐α could upregulate UCP1 expression (Barbera *et al*, 2001). UCP1 expression was also reported to be elevated in adipocytes in the absence of RIP140 (Debevec *et al*, 2007). Overall, FADD deletion‐associated reprogramming of glucose and lipid metabolism might contribute to obesity resistance. It is necessary to mention that, while the aP2‐Cre has been widely used to target adipose tissue, the specificity of this Cre for adipocytes has recently been questioned (Urs *et al*, 2006; Lee *et al*, 2013; Mullican *et al*, 2013). In addition to adipocytes, aP2 is also expressed in macrophages and dendritic cells; however, it is expressed in adipocytes approximately 10,000‐fold greater (Furuhashi & Hotamisligil, 2008). Furthermore, the use of aP2‐Cre mice reveals no recombination of loxP targets in macrophages (He *et al*, 2003). Fortunately, the aP2‐Cre transgene in our mouse model was shown to be specific to adipocytes. As such, FADD expression was not reduced in intraperitoneal‐macrophages from the ad‐FADD mice, consistent with previous studies using this aP2‐cre mouse line (Sabio *et al*, 2008; Qi *et al*, 2009; Li *et al*, 2011), showing adipocyte relatively restricted expression. However, in our future study, other adipocyte‐specific Cre, for example, PdgfRα‐Cre or adiponectin‐Cre (Jeffery *et al*, 2014), will be used for further investigation. But no matter which Cre is used, the tissue‐specific distribution needs to be investigated firstly before any study on specific mouse models with different genetic backgrounds.

We further generated and characterized mice mutant in both leptin and FADD by introducing the FADD‐D allele into the leptin‐deficient (*ob*/*ob)* mice to investigate the genetic influence of FADD in obesity. FADD‐D mutation on the background of *ob*/*ob* mice led to the generation of FADD‐D/*ob*/*ob* double‐mutant mice. Unlike *ob*/*ob* mice, these double mutants were resistant to leptin deficiency‐induced genetic obesity because of increased energy expenditure. Marked PPAR‐α‐mediated upregulation of genes participating in fatty acid oxidation in the adipose tissues may account for the excess energy expenditure in the FADD‐D/*ob*/*ob* double‐mutant mice. Leptin deficiency leads to obesity due to excess energy consumption (Friedman & Halaas, 1998); however, the FADD‐D mutation enhances energy expenditure to prevent the development of obesity caused by leptin deficiency, resulting in significant abdominal fat reduction. FADD‐D mice also showed this reduction in fat content, suggesting that FADD phosphorylation imparts an important role of increased energy expenditure through upregulating PPAR‐α function.

Despite similar even more food intakes, in WAT of FADD‐D and FADD‐D/*ob*/*ob* mice, the increased cAMP levels led to enhanced lipolysis, which consequently resulted in decreased adipocyte sizes with a reduction in triacylglycerol content. However, this effect might not fully account for the reduction of triacylglycerol in adipose tissue even though enhanced lipolysis in FADD‐D mice led to ectopic triacylglycerol content in the skeletal muscle and liver. In fact, FADD‐D and FADD‐D/*ob*/*ob* double‐mutant mice also exhibited increased oxygen consumption. Specially, *UCP1*,* UCP3*,* PPAR‐*δ, *Dio2*,* CPT1,* and *PGC1‐*α mRNA levels in FADD‐D WAT were significantly increased, reflecting higher thermogenesis and oxidation in WAT. This notion was further supported by better cold resistance of ad‐FADD mice compared with control littermates. UCP1 mRNA and protein expression in BAT of FADD‐D and ad‐FADD mice were also increased. UCP1 mRNA level in response to cold exposure or overfeeding has been reported to be upregulated due to the enhancement of sympathetic nerve activities (Thomas & Palmiter, 1997; Klingenspor, 2003), which is regulated via the transcription factor PGC‐1α (de Meis *et al*, 2005). In this study, we found that the expression of PGC‐1α was also elevated in FADD‐D mice, reflecting that elevated UCP1 level is likely regulated by PGC‐1α. However, it remains unclear if FADD‐D mutation or FADD deficiency has an indirect or direct effect on the regulation of UCP1 in BAT. FADD‐D mutation or FADD deficient might upregulate UCP1 expression, which can further enhance energy combustion by increasing thermogenesis. More fatty acids that were released from WAT would be consumed as a result of the increased energy expenditure, which would stimulate fat loss indirectly. Certainly, FADD‐D mutation might also affect both BAT and WAT. In addition, it has been reported that remarkable UCP1 expression in WAT may result in increased fatty acid oxidation in adipocytes and lead to resistance to diet‐induced obesity (Kopecky *et al*, 1996). Interestingly, serum FFA levels were decreased in FADD‐D mice, despite enhanced lipolysis in these mice. In FADD‐D mice, the molar ratio of FFA to glycerol released from adipose tissues was significantly decreased, reflecting that the utilization of fatty acids might be enhanced within adipose tissue. Consistently, fatty acid oxidation was observed to be enhanced in adipocytes from FADD‐D and FADD‐D/*ob*/*ob* double‐mutant mice relative to WT and *ob*/*ob* mice, respectively, suggesting that, enhanced utilization of fatty acid in adipocytes, at least partly, might account for increased energy expenditure. These results consistently demonstrate the function of FADD in fatty acid metabolism and energy expenditure.

Taken together, it appears that one of the consequences of FADD‐D mutation or FADD deficiency is to partition fat toward increased lipolysis and oxidation and the elevation in energy expenditure capacity is powerful enough to overcome increased food intake. Additionally, FADD‐D mutation prevents obesity in *ob*/*ob* mice and completely corrects the hypometabolic phenotype of leptin deficiency. Furthermore, we have demonstrated a possible mechanism regulating PPAR‐α signaling and function. These findings suggest that FADD might be a potential intervention target benefiting for the treatment of obesity, hepatic steatosis, and other metabolic disorders, and further research in this area will help to understand the pathology of human obesity‐related diseases.

## Materials and Methods

### RNA analysis

Total RNA was extracted from tissues using a TRIzol reagent (Invitrogen, Carlsbad, CA, USA) following the manufacturer's instructions and was used to prepare cDNA by PrimeScript RT reagent Kit (Takara, Otsu, Shiga, Japan). Quantitative real‐time PCR was performed with SsoFast EvaGreen Supermix on a CFX96 Real‐Time System (Bio‐Rad Laboratories, Hercules, CA, USA). The primers used in the quantitative real‐time PCR were listed in Appendix Table S4.

### Western blotting analysis

Proteins were separated by 12% SDS–PAGE and transferred to nitrocellulose membranes and probed the membrane with primary antibodies against UCP1 (1:1,000), p‐PKA substrate (1:500), desnutrin (1:1,000), total Akt (1:1,000), phospho‐Akt (Ser473) (1:1,000), ERK1/2 (1:1,000), phosphorylated ERK1/2 (1:1,000), phosphorylated PKC α/β II (1:500) or HSL (1:1,000) (Cell Signaling), PPAR‐α (1:1,000), FADD (1:500), phosphorylated FADD (1:500), RIP140 (1:1,000) (Abcam), phosphorylated HSL (1:1,000) (Calbiochem), VLCAD (1:500), LCAD (1:1,000), MCAD (1:1,000), GAPDH (1:1,000), β‐actin (1:1,000), or α‐tubulin (1:1,000) (Santa Cruz Biotechnology).

### Animals

All animals received human care according to Chinese legal requirements. The experiments were approved by Nanjing University Animal Care and Use Committee (20130118), and we strictly followed these rules during our experiments. FADD‐D mice were generated and maintained on C57BL/6J background in the laboratory of Dr. Astar Winoto (University of California, Berkeley) (Hua *et al*, 2003). The heterozygous leptin‐deficient *OB*/*ob* mice (The Jackson Laboratory, Bar Harbor, ME, USA) were crossed with FADD^+/−^/FADD‐D mice to generate FADD‐D/*ob*/*ob* double‐mutant mice and their littermates. Mice C57BL/6J and transgenic mice expressing Cre recombinase under control of an aP2 promoter (aP2‐Cre mice) were purchased from the Jackson Laboratory. C57/BL/6J mice containing the floxed FADD allele were bred with aP2‐Cre to generate aP2‐Cre^+^/FADD^loxP/loxP^ mice, thereafter referred to as ad‐FADD mice throughout the paper. The genotype was determined by PCR using primers listed in Appendix Table S5. Age‐matched male and female mice were used in this study. Each experimental group consisted of 3–12 mice. The animals were randomly allocated into experimental groups. The blinding process was employed in animal experiments. Mice were maintained in a temperature‐controlled (21°C) environment, using a 12‐h light–dark cycle, and were fed a SD or a HFD (45% of kcal from fat, 35% of kcal from carbohydrate and 20% of kcal from protein, Research Diets) *ad libitum*. EE was measured by indirect calorimetry. Oxygen consumption (VO_2_) was measured using the Oxymax system (Columbus Instruments), which was also used for continuous recording of locomotor activity through extensiometric weight transducers placed below the home cage. In order to study the role of PPAR‐α inhibition, either GW6471 (10 mg/kg) or its vehicle (DMSO 50% in saline) was injected intraperitoneally twice a week for a total of 10 weeks to control or ad‐FADD mice fed a HFD.

### Human protein expression analysis

We used subcutaneous adipose tissue samples from the Jiangsu Cancer Hospital. Immunoblot analysis was performed using antibody against FADD (1:500) (Cell Signaling). The human study protocol was approved by the Medical Ethics Committee of Jiangsu Cancer Hospital of China. All participants from all of the studies signed an informed consent form and the experiments conformed to the principles set out in the WMA Declaration of Helsinki and the Department of Health and Human Services.

### Electron microscopy

Epididymal fat pads and brown adipose tissue from WT and FADD‐D mice were fixed in 2% glutaraldehyde in 0.1 M cacodylate buffer, pH 7.4. The samples were postfixed with OsO_4_, dehydrated with alcohol, and embedded in epoxy embedding medium. Thin sections were cut at 70 nm thickness with a Leica EM UC6 ultramicrotome. Samples were stained with 2% uranyl acetate followed by Reynold's lead citrate. Electron microscope imaging of all the prepared materials was done with a Tencai G2 Spirit (FEI, Hillsboro, OR) operated at 80 kV.

### Oxygen consumption measurement

Brown adipocytes were isolated and oxygen consumption was measured using a Clark‐type oxygen electrode (Hansatech Instruments, Norfolk, UK), as previously described (Matthias *et al*, 2000), with minor modifications. Each sample was analyzed by incubating 1 × 10^6^ cells in a magnetically stirred chamber at 37°C. After the basal respiration was recorded, 5 mM oleate was added to determine the maximal oxygen consumption.

### Adipocyte isolation and SVCs isolation

Isolation of white epididymal adipocytes was based on a protocol previously described (Yang *et al*, 2004). Epididymal fat pads were removed from euthanized mice and minced into fine pieces in Krebs–Ringer–Hepes–BSA (KRHB) buffer (20 nM adenosine, 3 mM glucose, and 10 mg/ml bovine serum) with 1 mg/ml collagenase. Collagenase digestion was performed at 37°C for 1 h in a shaking water bath. Once digestion of adipose tissue was complete, the cell suspension was filtered through a 100‐μm filter (BD Biosciences) and washed several times in KRHB buffer, and the adipocytes were allowed to float and processed for protein or RNA extraction. SVCs were then pelleted from the infranatant as previously described (Li *et al*, 2011).

### Lipolysis assay

Lipolysis studies were performed in explants from freshly removed epididymal fat pads (about 20 mg) and in adipocytes isolated as previously described (Viswanadha & Londos, 2006) from gonadal WAT. Samples were incubated in Krebs–Ringer buffer (12 mM HEPES, 0.33 mM CaCl_2_, 1.2 mM MgSO_4_, 4.9 mM KCl, and 121 mM NaCl) with 0.1% glucose and 3.5% fatty acid‐free BSA, with or without 200 nM isoproterenol (Sigma) or 10 μM BAY (Sigma) and measured fatty acid (Sigma) and glycerol (Sigma) content. cAMP levels were determined by competitive immunoassay (R&D).

### Fatty acid oxidation

Fatty acid oxidation in adipocytes was determined as previously described (Bansode *et al*, 2008).

### Carcass and tissue analysis

Frozen, eviscerated carcasses from 25‐week‐old mice on a HFD were homogenized in water. The homogenates were dried to a constant weight and the lipid content was estimated by a previously described method (Bligh & Dyer, 1959). Tissue TAG was measured with a TAG 320A kit (Sigma) as described. Body composition was measured in non‐anesthetized mice by an Echo3‐in‐1 nuclear magnetic resonance (NMR) analyzer (Echo Medical Systems, Houston, TX).

### Blood chemistries

Plasma TAG concentration was determined by colorimetric kit assays (Roche Diagnostics). Plasma leptin levels were determined by the Linco Research (St. Charles, MO) Mouse Leptin RIA kit. Adiponectin serum levels were measured with a mouse adiponectin ELISA kit (RayBiotech). TNF‐α, IL‐6, IL‐12p40, and IL‐10 serum concentrations were measured with a mouse 23‐plex kit by Bio‐Plex 200 (Bio‐Rad).

### Glucose and insulin tolerance tests

For the GTT assay, mice were injected with d‐glucose (0.625 mg per g body weight) intraperitoneally after an overnight fast and the tail blood glucose levels were measured. For the ITT assay, mice were injected with insulin (humulin, Eli Lilly; 1.75 mU/g body weight) after a 5‐h fasting.

### *In vivo* insulin stimulation and analysis of insulin signaling

Mice were fasted overnight and then injected with five units of humulin via the vena cava. White adipose tissue, quadriceps, and liver were collected after 5 min and stored at −80°C until use. Total Akt (1:1,000) and phospho‐Akt (Ser473) (1:1,000) antibodies were purchased from Cell Signaling Technologies (Danvers, MA).

### Morphological, immunohistochemical, and immunofluorescence analysis

Tissues were fixed in neutral buffered formalin, embedded in paraffin, and sectioned at 5 μm onto poly‐l‐lysine‐coated slides. For histology, sections were stained with H&E. Adipocyte cell size was determined with ImageJ software (US National Institutes of Health), and at least 400 cells were measured from each sample. For immunohistochemistry, deparaffinized sections were immunostained with polyclonal rabbit anti‐mouse primary antibody to PPAR‐α (1:400) (Cell Signaling) according to standard immunohistochemistry procedures. For immunofluorescence analysis, samples were blocked in 5% BSA in PBS and then permeabilized in 0.3% Triton X‐100. Fat samples were incubated with primary antibodies in blocking buffer at 4°C for overnight. After washing, samples were incubated with fluorochrome conjugated secondary antibodies for 1 h at room temperature. Fat pads were imaged on a microscopy (Carl Zeiss Axioplan 2). Anti‐caveolin1 (1:200) and F4/80 (1:200) were purchased from BD Biosciences and Abcam, respectively.

### 2‐Deoxyglucose (2‐DOG) uptake

The assay for glucose uptake was performed as previously described (Nguyen *et al*, 2007).

### Magnetic resonance imaging analysis

Mice were scanned by using a 4.7T Varian system (Bruker Medical). Whole‐body images (between 40 and 45 slices, each 2 mm thick) were obtained for each mouse by using a spin‐echo sequence [repetition time (TR)/echo time (TE) = 4,500:20].

### Chromatin immunoprecipitation (ChIP) assay

ChIP assay in MEFs or isolated adipocytes was performed as previously described (Goto *et al*, 2011). The antibody to PPAR‐α (1:50) or FADD (1:50) (Abcam) or normal mouse IgG (Santa Cruz Biotechnology) was used for immunoprecipitation, and oligonucleotide primers composed of the following sequences were used for PCR: *CPT1b*, 5′ ‐CCTGTGCTGGTCCCCAACTCACAGC‐ 3′ and 5′ ‐CTCCTGGTGACCTTTTCCCTACATT‐ 3′ (279 bp).

### Statistical analyses

The results were assessed by Student's *t*‐test to compare two groups or analysis of variance (ANOVA) followed by Bonferroni's *post hoc* test for multiple comparisons and were expressed as means ± SEM. For animal experiments, the mice were randomly allocated into experimental groups and the blinding process was employed. For all statistical analyses, GraphPad Prism 5 software for Windows was used (GraphPad Software, San Diego, CA, USA). A *P*‐value < 0.05 was considered to be significant.

## Author contributions

ZCH conceived and initiated the project. ZCH and HZ planned the experiments. HZ performed most experiments, including MRI, ChIP assay, GTT, ITT, animal model experiments. XW, DZ, FC, and ZG participated in cell culture and Western blotting analysis. PD, YY, BY, XZi, CY, YZ, CJ, and SG participated in animal breeding and genotyping. XZu and JZ participated in acquisition of data. WJ and QH participated in morphological and immunohistochemical analysis. ZCH and HZ wrote the manuscript.

## Conflict of interest

The authors declared that they have no conflict of interest.

**The paper explained**

**Problem**

Obesity in adults as well as children is a global health problem, resulting from an imbalance between caloric intake and energy expenditure. The increasing prevalence of the metabolic syndrome and obesity calls for new approaches for the prevention and management of these diseases. However, signaling pathways controlling the advancing of this disease are still not well understood.

**Results**

We present here that FADD, a classic apoptotic adaptor protein in death receptor signaling, is a master regulator of fat and glucose metabolism with potential applications for treatment of obesity and insulin resistance. We show that FADD interacts with RIP140, which is a corepressor for PPAR‐α, and FADD phosphorylation‐mimic mutation (FADD‐D) or FADD deficiency abolishes RIP140‐mediated transcriptional repression, leading to the activation of PPAR‐α. As a result, FADD‐D mutation or adipose‐specific FADD disruption in mice prevents obesity induced by leptin deficiency or by feeding on a HFD.

**Impact**

Besides its classic apoptotic role in death receptor signaling, FADD is firstly found to be involved in lipid metabolism. We have also demonstrated a possible mechanism regulating PPAR‐α signaling and function. These findings suggest that FADD might be a potential intervention target benefiting for the treatment of obesity, hepatic steatosis, and other metabolic disorders that deserve further investigation.

## Supporting information



Appendix

Review Process File

Source Data for Appendix

Source Data for Figure 1

Source Data for Figure 4

Source Data for Figure 5

Source Data for Figure 7

Source Data for Figure 10
