# Supplementary material for: FADD is a key regulator of lipid metabolism
Source: EMBO Mol Med. 2016 Jun 29;8(8):895–918. doi: 10.15252/emmm.201505924 (PMC4967943; doi:10.15252/emmm.201505924)
Supplement: Supplementary file 1 — Appendix [file EMMM-8-895-s001.pdf]

APPENDIX TABLE OF CONTENTS

**APPENDIX TABLE S1**	**Page 2**
**APPENDIX TABLE S2**	**Page 3**
**APPENDIX TABLE S3**	**Page 4**
**APPENDIX TABLE S4**	**Page 5**
**APPENDIX TABLE S5**	**Page 7**
**APPENDIX FIGURE S1**	**Page 8**
**APPENDIX FIGURE S2**	**Page 11**
**APPENDIX FIGURE S3**	**Page 12**
**APPENDIX FIGURE S4**	**Page 13**
**APPENDIX FIGURE S5**	**Page 14**
**APPENDIX FIGURE S6**	**Page 15**
**APPENDIX FIGURE S7**	**Page 16**
**APPENDIX FIGURE S8**	**Page 17**
**APPENDIX FIGURE S9**	**Page 18**
**APPENDIX FIGURE S10**	**Page 20**
**APPENDIX FIGURE S11**	**Page 22**
**APPENDIX FIGURE S12**	**Page 24**

Appendix Table S1. Body composition data for male WT and FADD-D mice.

	**WT (n = 5)**	**FADD-D (n = 5)**	***P* values**
Body weight (g)	41.51 ± 3.57	13.43 ± 1.36	$P = 0.0007$
Carcass weight (g)	32.18 ± 2.52	9.92 ± 0.89	$P = 0.0006$
Water (%)	42.67 ± 1.93	66.86 ± 1.78	$P = 0.0047$
Lipid (%)	41.39 ± 2.45	7.32 ± 0.64	$P = 0.0003$
Lean Tissue (%)	15.94 ± 1.32	25.82 ± 0.93	$P = 0.0044$

Body composition data for male WT and FADD-D mice age 18 weeks fed a HFD.

Appendix Table S2. Organ weight of control and ad-FADD mice.

**Organs**	**Control (n = 6)**	**ad-FADD (n = 6)**	***P* values**
Epididymal fat (mg)	1068 ± 68	716 ± 32	$P = 0.0059$
Perirenal fat (mg)	528 ± 38	339 ± 28	$P = 0.0044$
Heart (mg)	182 ± 7	175 ± 6	$P = 0.4911$
Liver (mg)	1308 ± 84	1268 ± 65	$P = 0.1539$
Spleen (mg)	118 ± 11	113 ± 5	$P = 0.3547$
Kidney (mg)	189 ± 8	179 ± 5	$P = 0.0922$

Appendix Table S3. Metabolic parameters of control and ad-FADD mice after 12 weeks of HFD.

**Genotype**	**Control (n = 6)**	**ad-FADD (n = 6)**	***P* values**
Fasting glucose (mg/dl)	152 ± 34.6	112 ± 20.9	$P = 0.0295$
Fed glucose (mg/dl)	188 ± 24.4	127 ± 27.2	$P = 0.0073$
Fasting serum insulin (ng/ml)	2.1 ± 0.42	0.8 ± 0.16	$P = 0.0008$
Fasted serum triglyceride (mg/dl)	145 ± 37.3	88.6 ± 17.2	$P = 0.0069$
Fasted serum FFA (mM)	1.62 ± 0.41	0.93 ± 0.35	$P = 0.0084$
Fasted serum cholesterol (mg/dl)	168 ± 35.7	145 ± 22.8	$P = 0.0897$

Data are expressed as mean ± SEM.

Appendix Table S4. The primers of selected genes for real time PCR.

Gene	Forward primer (5'-3')	Reverse primer (5'-3')
Acox1	CGCCGCCACCTTCAATCCAGAG	TCCAGGCCGGCATGAAGAAAC
Acadvl	CCCATGGGCTCCCTGAAAAGAAGA	GGCCGCCTCCGAGCAAAAGAT
Acadm	TCGCCCCGGAATATGACAAAA	AGAACGTGCCAACAAGAAATACCA
Acaca	GTTTGCTGGCCAGTGCTATGCT	GGGGATCCGGCCAGAGACA
Adiponectin	GTTGCAAGCTCTCCTGTTCC	ATCCAACCTGCACAAGTTCC
Arginase	ATGGAAGAGACCTTCAGCTAC	GCTGTCTTCCCAAGAGTTGGG
CD11c	ACACAGTGTGCTCCAGTATGA	GCCCAGGGATATGTTCACAGC
COX2	CAGTCAGGACTCTGCTCACGAA	AGCAGCACAGCTCGGAAGA
CPT1	CGGCGGCCCATGCTCTACAG	GGTCCAGTTTGCGGCGATACAT
Dgat1	GGCCTGCCCCATGCGTGATTA	GGGGGACCGAGGAAAGTTGAGTT
Dio2	GAAGGGCTGCGCTGTGTCTGG	TCCTCTTGGTTCCGGTGCTTCTTA
Ehhadh	TCCCCCACTACCATCGCCACAG	ACCAAATCGCCCAGCTTCACAGAG
FADD	ACGACCTGGAGCGCGGGCACAC	TTCGGGGGTACTTCTCCTCAATCC
Fat	GACCCCGAGGACCACACTG	GGAAAGGAGGCTGCGTCTG
Fasn	ACTGGCGTCTGGGTGGGTGTGAG	CCGCCGAGCCAGGGACTTCTTAG
F4/80	CTTTGGCTATGGGCTTCCAGTC	GCAAGGAGGACAGAGTTTATCGTG
GAPDH	AACGACCCCTTCATTGAC	TCCACGACATACTCAGCAC
Hsl	TGCGCCCCACGGAGTCTATGC	GCTTGAGCGCCGGGGGTGAAA
IFNγ	TCAAGTGGCATAGATGTGGAAGAA	TGGCTCTGCAGGATTTTCATG
IL-1β	AAATACCTGTGGCCTTGGGC	CTTGGGATCCACACTCTCCAG
IL-6	CCAGAGATACAAAGAAATGATGG	ACTCCAGAAGACCAGAGGAAAT
IL12p40	CCAGAGACATGGAGTCATAG	AGATGTGAGTGGCTCAGAGT
IL-4	ATGGAGCTGCAGAGACTCTT	AAAGCATGGTGGCTCAGTAC
IL-10	TGAATTCCCTGGGTGAGAAG	TCACTCTTCACCTGCTCCACT
iNOS	GAGGCCCAGGAGGAGAGAGATCCG	TCCATGCAGACAACCTTGGTGTTG
Lpl	GTATCGGGCCCAGCAACATTATCC	GCCTTGCTGGGGTTTTCTTCATTC

MCP-1	AGGTCCCTGTCATGCTTCTG	GCTGCTGGTGATCCTCTTGT
Mgl1	ATGATGTCTGCCAGAGAACC	ATCACAGATTTCAGCAACCTTA
PPARδ	GAGGCCCGGGAAGAGGAGAAAGA	GTCGGCCAGCTGGGGGTTGT
PGC-1	CACGCAGCCCTATTCATTGTTCG	GACTCCCGCTTCTCGTGCTCTTT
PPARα	TCGCGGGAAAGACCAGCAACAA	GCCAGGCCGATCTCCACAGC
TNFα	CCAGACCCTCACACTCAGATC	CACTTGGTGGTTTGCTACGAC
Ucp1	CGCTACACGGGGACCTACAATG	ACCCGAGTCGCAGAAAAGAAGC
Ucp3	AGAACCATCGCCAGGGAGGAAGGA	CACCGGGGAGGCCACCACTGT
Ym1	GGGCATACCTTTATCCTGAG	CCACTGAAGTCATCCATGTC

Appendix Table S5. The primers for genotyping.

**FADD-neo**	
mFADD-F-6390to6414	5’- ACTGTAGTGCCCAGCAGAGACCAGC -3’
TKp-R	5’- CGCTCGGTGTTCGAGGCCACACGC -3’
**Tg-FADD-D**	
FADD-D-Tg-F	5’- AGCGGGTAAGGGAGAGTCTGAAAG -3’
FADD-D-Tg-R	5’- AATCCCTTAGTACGGGGT -3’
ob	
db2-F	5’- ACCTGGAGAATCTCT -3’
db2G/T-R	5’- CTCTTATCTCTACTTGCT -3’
***OB* (Leptin)**	
DB2-F	5’- AGAGTATGACTAGGAGGG -3’
DB2-R	5’- GATGGAGGAGGTCTCG -3’
**aP2-Cre**	
cCre-F	5’ -GCGGTCTGGCAGTAAAAACTATC- 3'
cCre-R	5’ -GTGAAACAGCATTGCTGTCACTT- 3’
**tgLoxp-FADD**	
tgFADD-Loxp-R	5’ -GTATGCTATACGAAGTTATAAGCTTAGG- 3’
tgFADD-Loxp-F	5’ -GAGGATCTTAGCCACAGGTTC- 3’

Appendix Fig S1

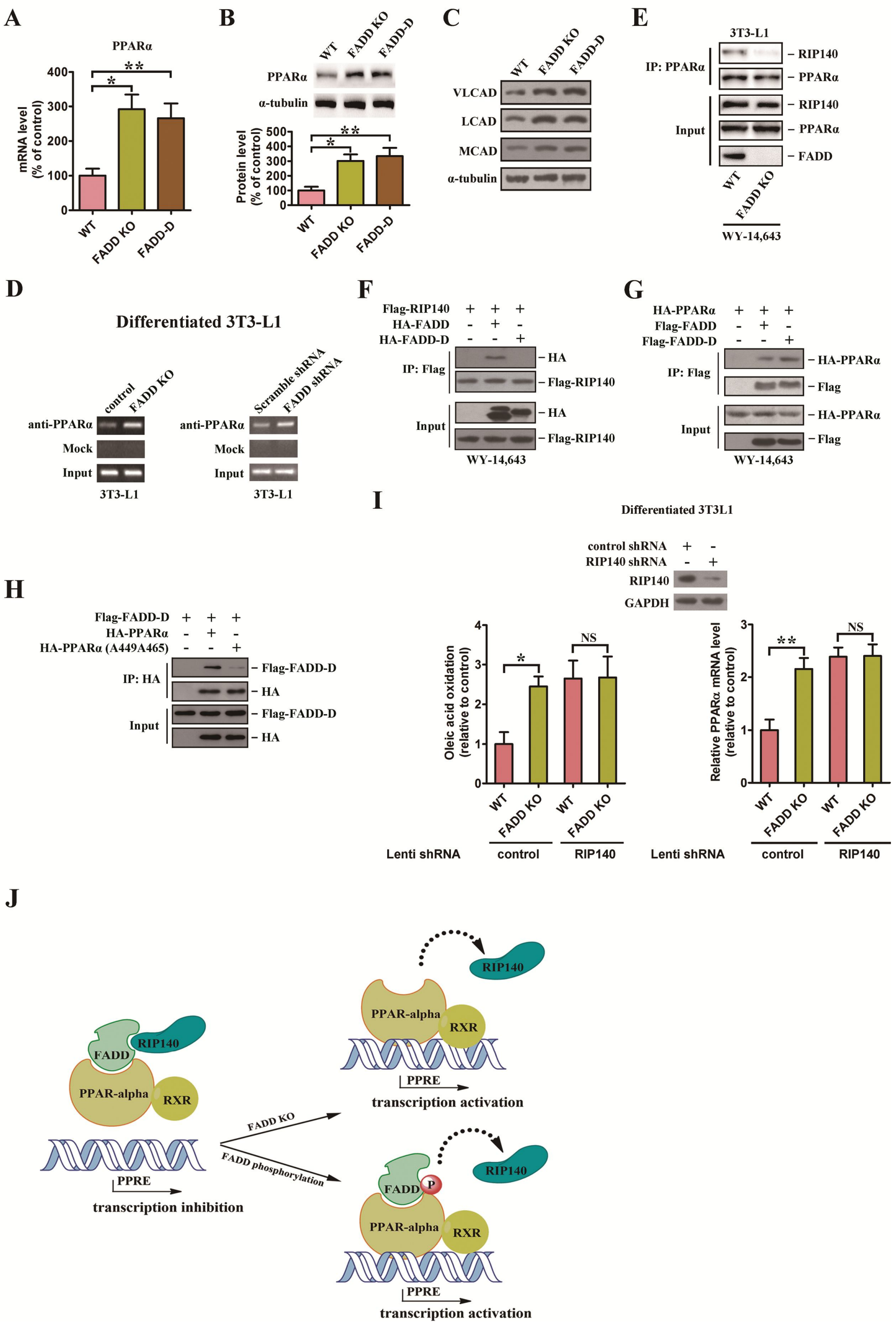

Appendix Fig S1. FADD-D mutation or FADD deficiency leads to enhanced PPAR-α activity.

A Quantitative real time PCR for *PPAR-α*, using RNA from wild-type, FADD knockout and

FADD-D mutant MEFs. Data are expressed as mean ± SEM from four independent experiments. *P = 0.0037, **P = 0.0052 (one-way ANOVA).

B Immunoblotting of PPAR-α and Tubulin (control) (top) with relative quantification (bottom). Data are expressed as mean ± SEM from three independent experiments. *P = 0.0039, **P = 0.0023 (one-way ANOVA).

C Immunoblot of VLCAD, LCAD, MCAD and Tubulin (control), using protein extracts from wild-type, FADD knockout and FADD-D mutant MEFs. The same results were obtained in other independent experiments.

D Left, ChIP assay of *CPT1b* promoter in wild-type and FADD knockout 3T3-L1 adipocytes. Soluble chromatin from differentiated 3T3L1 cells was immunoprecipitated with control mouse IgG or antibodies against PPAR-α. Immunoprecipitates were analyzed by PCR using specific primers for the mouse *CPT1b* promoter region containing potential PPRE. Right, ChIP assay of *CPT1b* promoter in 3T3L1 adipocytes infected with lentivirus containing FADD shRNA or scrambled shRNA control. The experiments were performed in triplicate.

E PPAR-α and RIP140 coimmunoprecipitate in wild-type but not FADD knockout 3T3L1 adipocytes. PPAR-α was immunoprecipitated (IP) from the cell lysates of wild-type or FADD knockout 3T3L1 adipocytes. The same results were obtained in other independent experiments.

F Coimmunoprecipitation analysis of FADD and RIP140 in 293T cells. As indicated, lysates from the cells transfected with plasmids encoding Flag-RIP140, HA-FADD or HA-FADD-D were subjected to immunoprecipitation with an anti-Flag antibody. FADD and

FADD-D were detected using an anti-HA antibody. The experiments were performed in triplicate.

G Coimmunoprecipitation analysis of FADD and PPAR-α in 293T cells. As indicated, lysates from the cells transfected with plasmids encoding Flag-FADD, Flag-FADD-D or HA-PPAR-α were subjected to immunoprecipitation with an anti-Flag antibody. PPAR-α was visualized by anti-HA. The experiments were performed in triplicate.

H Mutation of PPAR-α K449 and R465 to alanine led to reduced interaction between FADD-D and PPAR-α. As indicated, lysates from the cells transfected with plasmids encoding Flag-FADD-D, HA-PPAR-α or HA-PPAR-α (A449A465) were subjected to immunoprecipitation with an anti-HA antibody. FADD-D was visualized by anti-Flag. The same results were obtained in other independent experiments.

I Left, β-oxidation analysis in WT or FADD KO 3T3L1 adipocytes infected with RIP140 shRNA or scrambled shRNA lentivirus. Right, mRNA level of *PPAR-α* in WT or FADD KO 3T3L1 adipocytes infected with RIP140 shRNA or scrambled shRNA lentivirus. RIP140 expression was also shown (*upper*). Data are expressed as mean ± SEM from three independent experiments. *P = 0.0092, **P = 0.0127 (one-way ANOVA). NS, not statistically significant.

J A putative molecular model for how FADD regulates the transcriptional activity of PPAR-α.

Appendix Fig S2

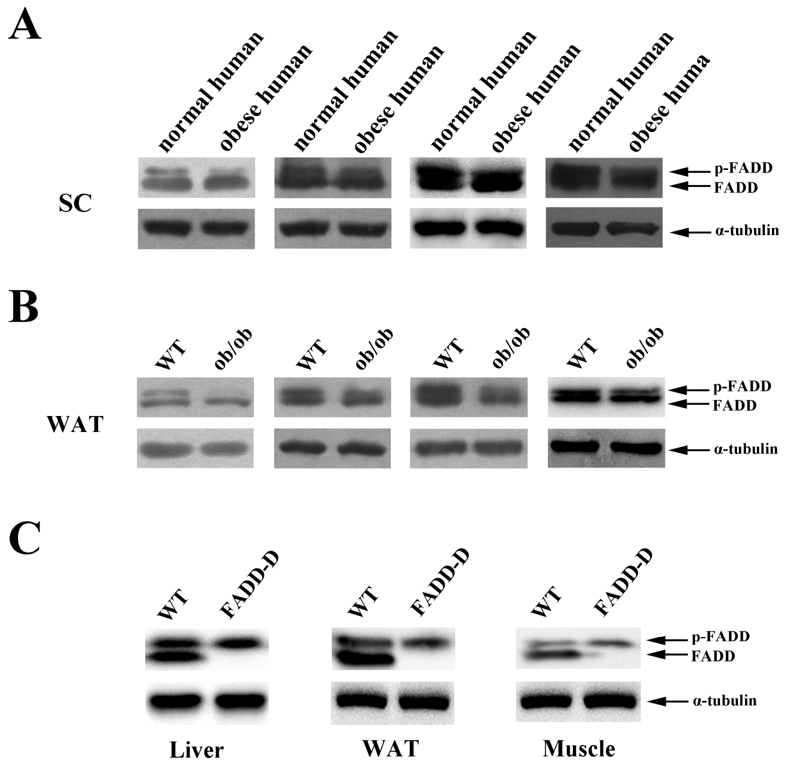

Appendix Fig S2. Expression of FADD in metabolic tissues of human and mice.

A Immunoblot of FADD protein in subcutaneous (SC) adipose tissues of normal and obese human. Data shown are representative of three independent experiments having similar results. (n = 15 total per group).

B Immunoblot of FADD protein in WAT of WT and *ob/ob* obese mice. Data shown are representative of three independent experiments having similar results. (n = 12 total for each genotype).

C Immunoblot of FADD protein in three metabolic tissues of WT and FADD-D mice. The same results were obtained in other independent experiments. Data shown are representative of three independent experiments having similar results. (n = 4 total for each genotype).

Appendix Fig S3

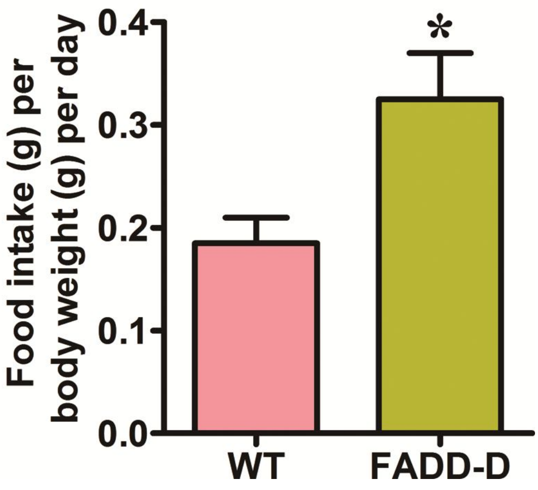

Appendix Fig S3. Food intake per mouse fed a SD measured over 20 days normalized by body weight (n = 12 for each genotype). Results are means ± SEM. *P = 0.0102 (Student's *t*-test).

Appendix Fig S4

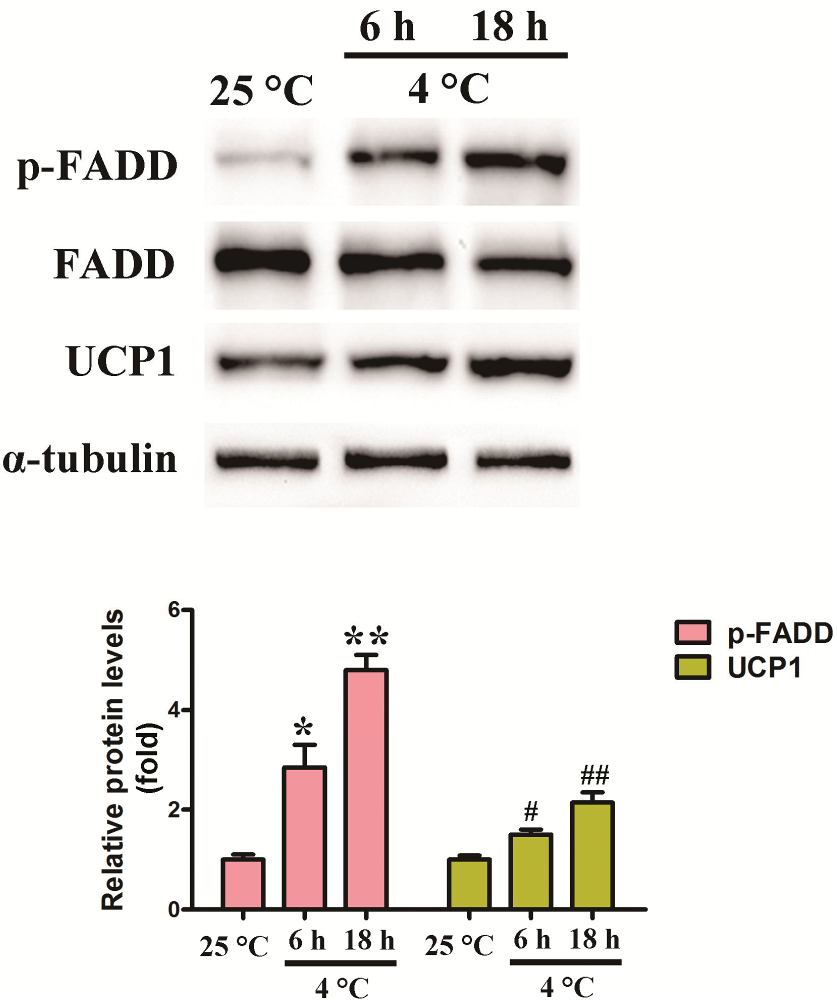

Appendix Fig S4. Phosphorylated FADD and UCP1 expression during cold exposure.

HFD-fed mice maintained at room temperature were placed at 4 °C for 18 h. Protein levels of p-FADD or UCP1 at the indicated time points were shown. Data shown are from one experiment, representative of a total of four independent experiments. Results are means ± SEM. *P = 0.0081, **P = 0.0015, $^{\#}P$ = 0.0189, $^{\#\#}P$ = 0.0094 (one-way ANOVA).

Appendix Fig S5

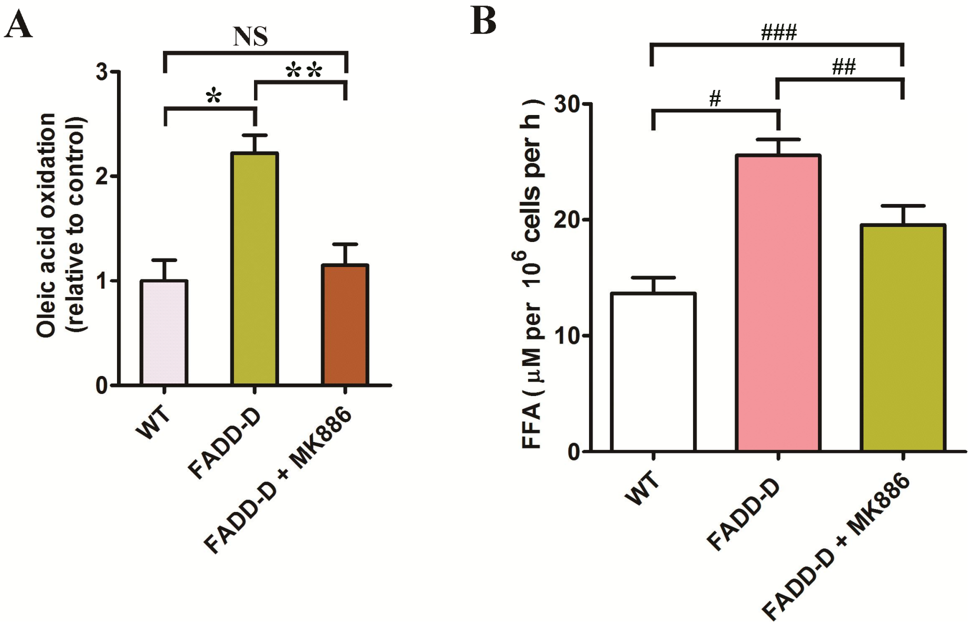

Appendix Fig S5. MK886 inhibits fatty acid oxidation and lipolysis in white adipocytes isolated from FADD-D mice.

A-B Fatty acid oxidation **(A)** and lipolysis **(B)** in white adipocytes isolated from WT and FADD-D mice treated with or without 5 μM MK886 (n = 5 for each genotype). Data are expressed as mean ± SEM. $*P = 0.0047$, $**P = 0.0055$, $^{\#}P = 0.0061$, $^{\#\#}P = 0.0092$, $^{\#\#\#}P = 0.0101$ (one-way ANOVA). NS, not statistically significant.

Appendix Fig S6

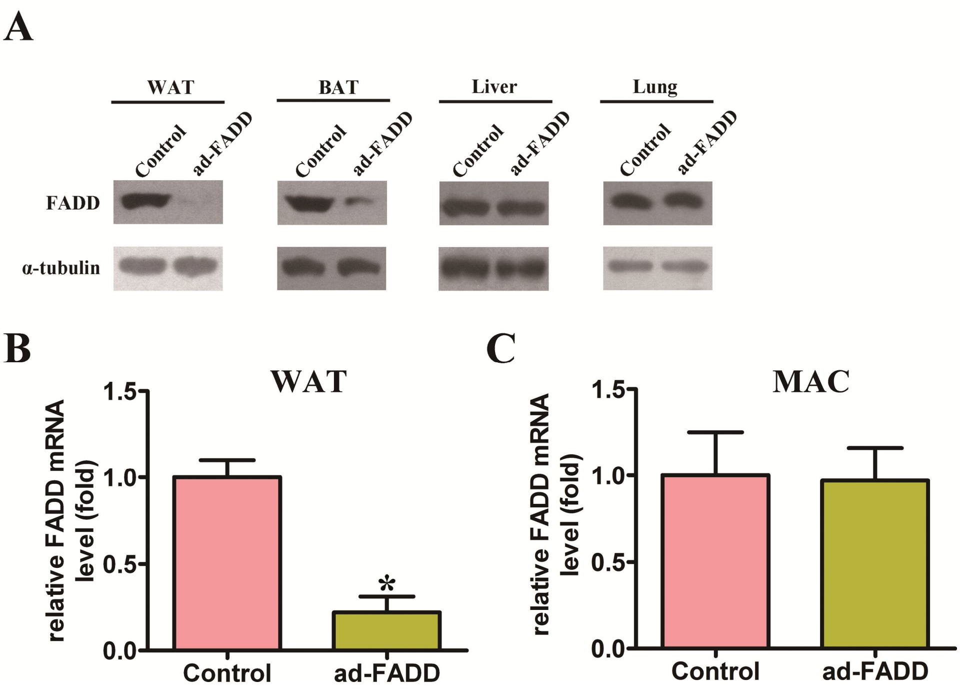

Appendix Fig S6. Adipose-specific disruption of the FADD gene *via* Cre-loxP-mediated recombination.

A Western blot analysis for FADD in epididymal white adipose tissue (WAT), brown adipose tissue (BAT), liver and lung in control and ad-FADD mice is shown. As a loading control, Western blotting for α-tubulin was performed. Data shown are representative of three independent experiments having similar results.

B The result of quantitative real time PCR analysis of WAT for *FADD* in control and ad-FADD mice is shown in the bar graph. Data are expressed as mean ± SEM from four independent experiments. *$P = 0.0004$ (Student's *t*-test).

C Relative mRNA levels of *FADD* in macrophages from control and ad-FADD mice. Data are expressed as mean ± SEM from four independent experiments.

Appendix Fig S7

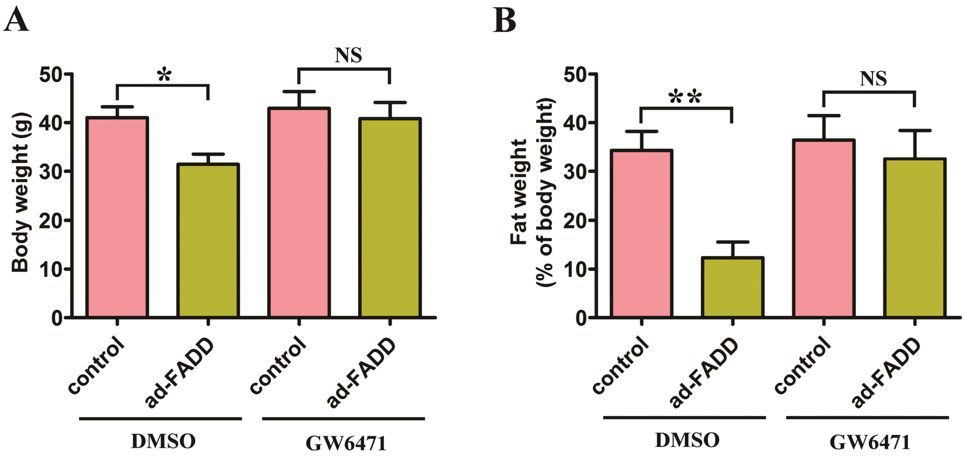

Appendix Fig S7. Effects of PPAR-α antagonist GW6471 on body weight and fat weight of mice fed a HFD.

A-B **(A)** Body weight and **(B)** fat weight of control and ad-FADD mice with treatment of DMSO or GW6471 maintained on a HFD for 10 weeks (n = 8 for each genotype). Results are means ± SEM; **P* = 0.0072, ***P* = 0.0009 (one-way ANOVA). NS, not statistically significant.

Appendix Fig S8

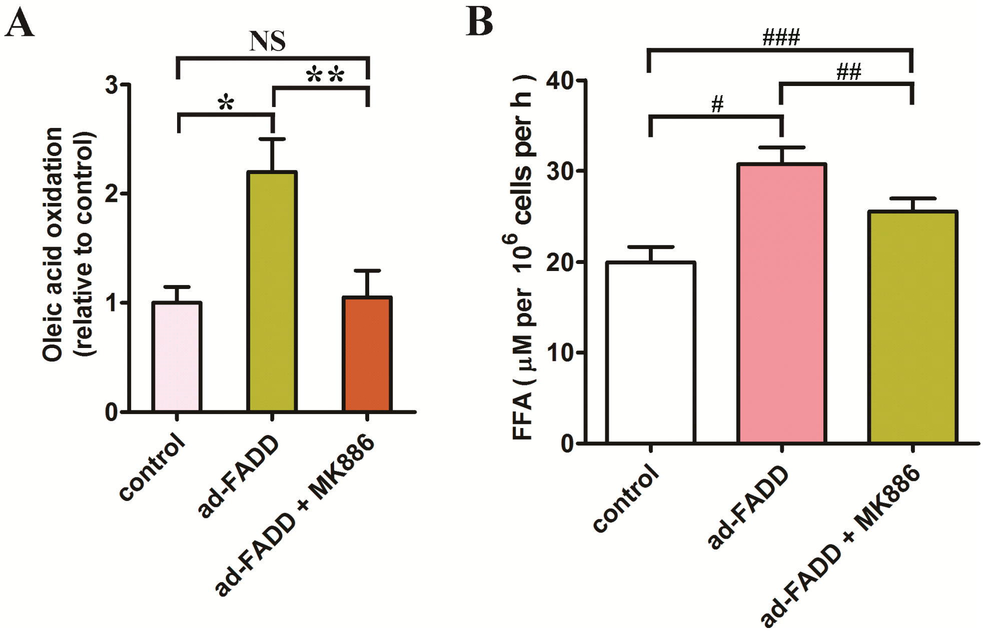

Appendix Fig S8. MK886 inhibits fatty acid oxidation and lipolysis in white adipocytes isolated from ad-FADD mice.

A-B Fatty acid oxidation **(A)** and lipolysis **(B)** in white adipocytes isolated from control and ad-FADD mice treated with or without 5 μM MK886 (n = 6 for each genotype). Results are means ± SEM. *P = 0.0075, **P = 0.0093, $^{\#}P$ = 0.0114, $^{\#\#}P$ = 0.0159, $^{\#\#\#}P$ = 0.0183 (one-way ANOVA). NS, not statistically significant.

Appendix Fig S9

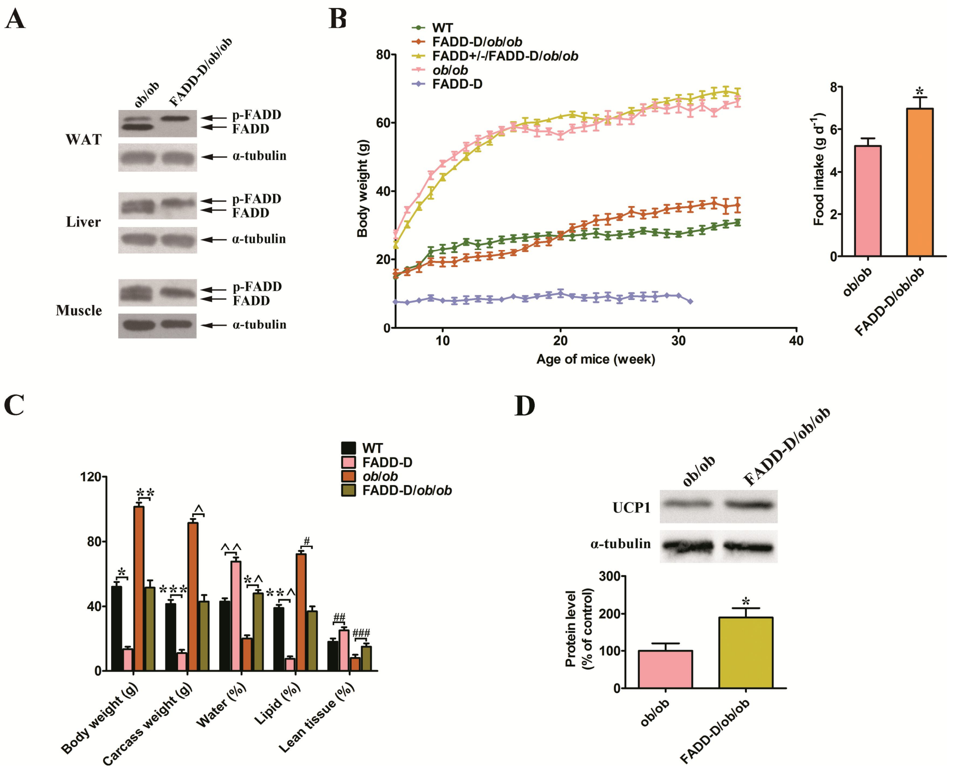

Appendix Fig S9. FADD-D mutation reverses the obesity phenotype of *ob*/*ob* mice.

A Immunoblot of FADD protein in three metabolic tissues of *ob*/*ob* and FADD-D/*ob*/*ob* mice. Data shown are representative of three independent experiments having similar results.

B Left, body weights of male mice on a SD (n = 8 for each genotype). Right, food intake in 10-week-old male mice fed a SD (n = 6 for each genotype). Data are expressed as mean ± SEM. **P* = 0.0218 (Student's *t*-test).

C Carcass analysis of 25-week-old male mice fed a HFD (n = 6 for each genotype). Data are expressed as mean ± SEM. **P* = 0.0013, ***P* = 0.0033, ****P* = 0.0025, ^*P* = 0.0029, ^^*P* = 0.0064, *^*P* = 0.0023, **^*P* = 0.0008, $^{\#}P$ = 0.0027, $^{\#\#}P$ = 0.0216, $^{\#\#\#}P$ = 0.0133 (one-way ANOVA).

D Immunoblotting of UCP1 and Tubulin (control) (top) with relative quantification

(bottom), using total cell lysates of BAT from 20-week-old male *ob*/*ob* and FADD-D/*ob*/*ob* mice fed a SD. Data shown are from one experiment, representative of a total of three independent experiments. Results are means ± SEM. *P = 0.0107 (Student's *t*-test).

Appendix Fig S10

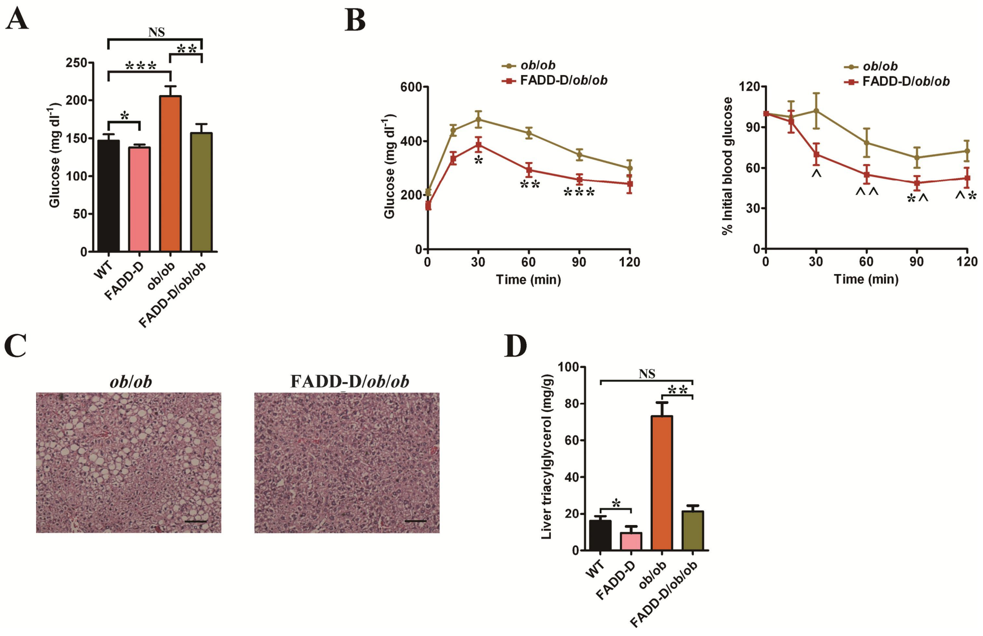

Appendix Fig S10. FADD-D mutation reverses hyperglycemia and reduces hepatic steatosis of *ob*/*ob* mice.

A Plasma glucose levels were measured in 15-week-old male mice fed a SD (n = 6 for each genotype). Data are expressed as means ± SEM. **P* = 0.0359, ***P* = 0.0092, ****P* = 0.0075 (one-way ANOVA). NS, not statistically significant.

B GTT (left) and ITT (right) in 15-week-old male *ob*/*ob* and FADD-D/*ob*/*ob* mice fed a SD (n = 5 for each genotype). Data are expressed as means ± SEM. **P* = 0.0242, ***P* = 0.0057, ****P* = 0.0133, ^*P* = 0.0072, ^^*P* = 0.0094, *^*P* = 0.0185, ^**P* = 0.0275 (Student's *t*-test).

C H&E-stained liver sections from representative *ob*/*ob* and FADD-D/*ob*/*ob* mice. Images are 200 × magnifications; scale bars denote 100 μm. Shown are typical results from four different fields and three different experiments.

D Liver triacylglycerol content of 15-week-old WT, FADD-D, *ob*/*ob*, and FADD-D/*ob*/*ob* mice fed a SD (n = 6 for each genotype). Data are expressed as means ± SEM. **P* = 0.0292,

**P = 0.0009 (one-way ANOVA). NS, not statistically significant.

Appendix Fig S11

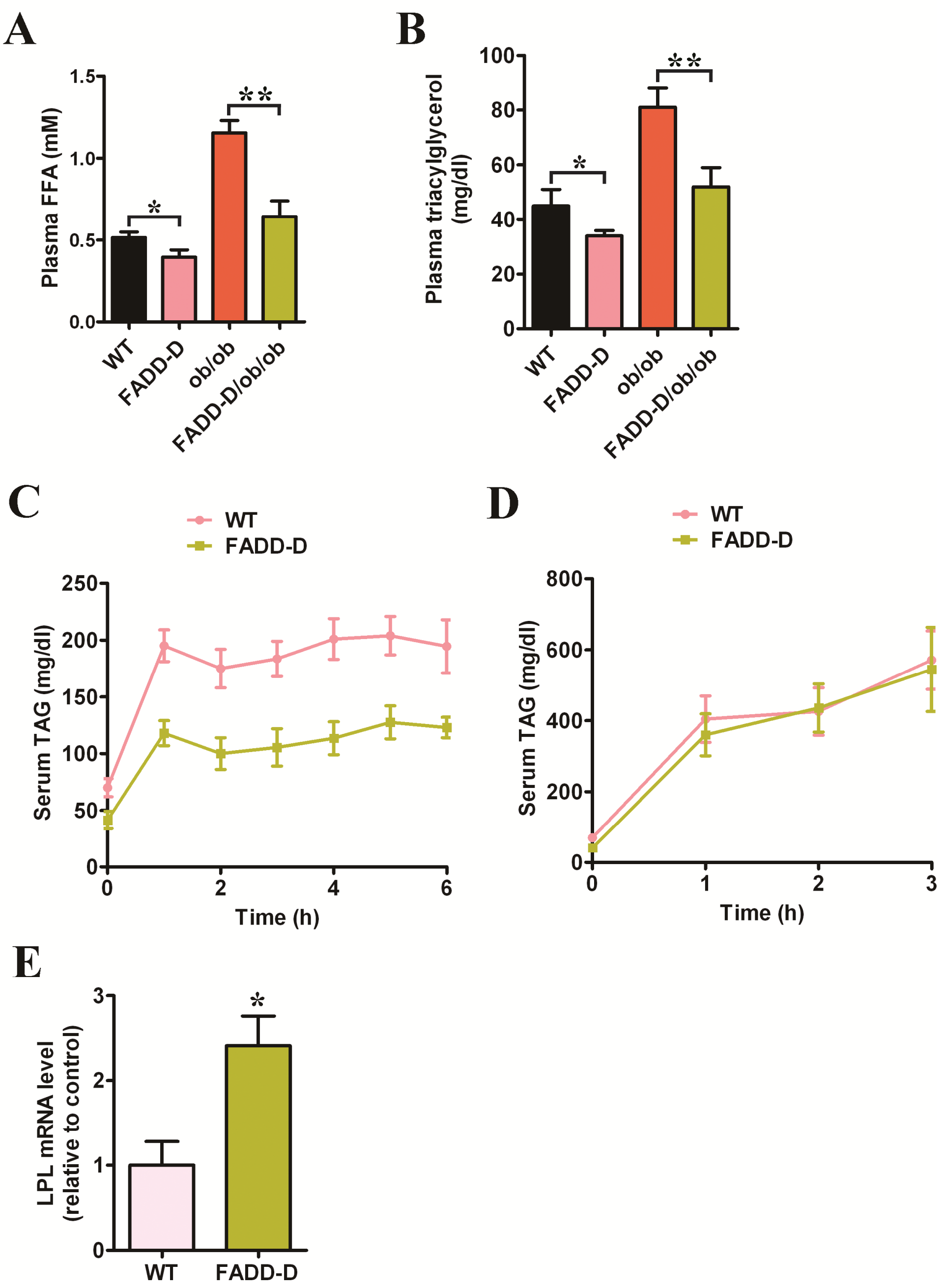

Appendix Fig S11. Serum parameters and TAG clearance.

A Serum free fatty acids (FFA) concentrations in WT, FADD-D, *ob*/*ob* and FADD-D/*ob*/*ob* double mutant mice fed a SD (n = 6 for each genotype). Data are expressed as means ± SEM. **P* = 0.0233, ***P* = 0.0082 (one-way ANOVA).

B Serum triacylglycerol concentrations in WT, FADD-D, *ob*/*ob* and FADD-D/*ob*/*ob* double mutant mice fed a SD (n = 5 for each genotype). Data are expressed as means ± SEM. **P* = 0.0242, ***P* = 0.0111 (one-way ANOVA).

C Serum TAG clearance. Serum TAG levels of overnight fasted mice were followed for 6 h following oral gavage with 400 μl peanut oil (n = 3 for each genotype), as described previously (Columbo et al., J. Biol. Chem. (278) 3992-3999, 2003).

D Intestinal TAG absorbance and secretion. Serum TAG levels were measured in mice given a tail vein injection of WR1339 (to inhibit lipoprotein lipase) prior to gavage with 400 μl peanut oil (n = 5 for each genotype).

E Lipoprotein lipase mRNA levels in the liver of WT and FADD-D mice, normalized to GAPDH, as determined by quantitative real time PCR. Data are expressed as mean ± SEM from four independent experiments. **P* = 0.0117 (Student's *t*-test).

Appendix Fig S12

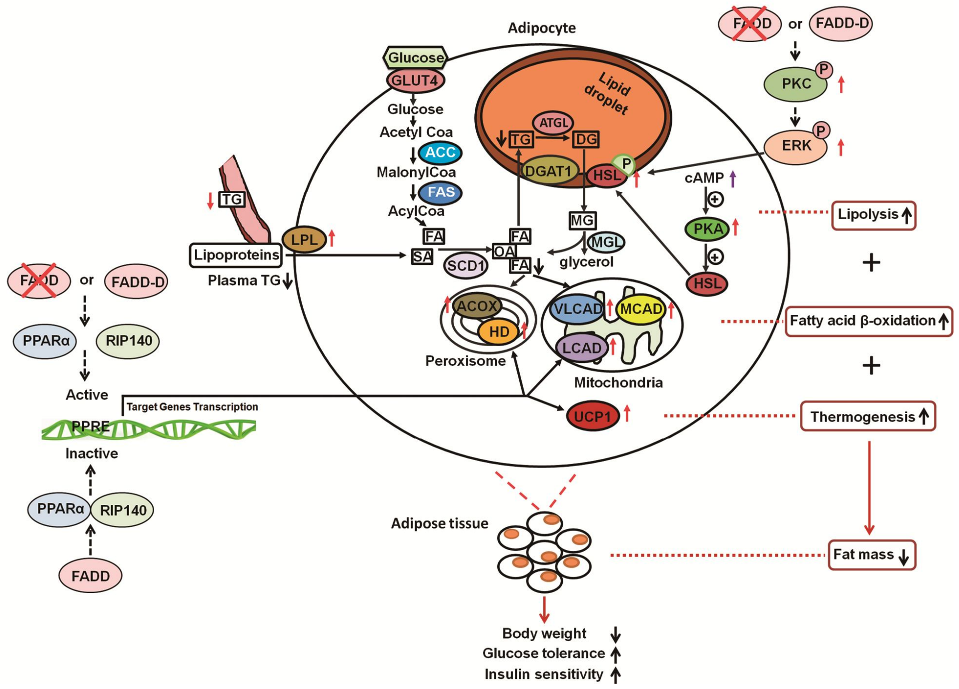

Appendix Fig S12. Summary of the metabolic effects of FADD.

FADD-D mutation or FADD deficiency abolished RIP140-mediated transcriptional repression, leading to the activation of PPAR-α and the subsequently enhancement of fatty acid β-oxidation and thermogenesis, which finally prevents obesity induced by high-fat feeding or leptin deficiency. FADD-D mutation increases cAMP levels and activates HSL in adipose tissue, resulting in increased lipolysis. FADD deficiency or FADD-D mutation might also increase HSL activity through activating PKC and ERK. FADD-D mutation or FADD deficiency activates PPAR-α by enhancing its binding to PPRE located in the promoter region of genes involved in fatty acid oxidation. The action of FADD on fatty acid β-oxidation is thus exerted by direct activation of PPAR-α target genes. Red arrows indicate the direction (up or down) of regulation. Abbreviations: ATGL (patatin-like phospholipase domain containing 2), DG (diglycerides), DGAT1 (diacylglycerol O-acyltransferase homolog 1), MG (monoglycerides), MGL (monoglyceride lipase), FA (fatty acid), FAS (fatty acid synthase),

ACC (acetyl-coenzyme A carboxylase alpha), GLUT4 (glucose transporter-4), HD (enoyl-CoA hydratase/3-hydroxyacyl-CoA dehydrogenase), HSL (hormone-sensitive lipase), PKA (protein kinase A), LPL (lipoprotein lipase), ACOX (acyl-CoA oxidase 1), VLCAD (very long chain acyl-CoA dehydrogenase), LCAD (long chain acyl-CoA dehydrogenase), MCAD (medium chain acyl-CoA dehydrogenase), OA (oleic acid), SA (stearic acid), PPAR-α (peroxisome proliferator-activator receptor-alpha).
