# Supplementary figures and images for: FADD is a key regulator of lipid metabolism
Source: EMBO Mol Med. 2016 Jun 29;8(8):895–918. doi: 10.15252/emmm.201505924 (PMC4967943; doi:10.15252/emmm.201505924)

Appendix Fig S1B

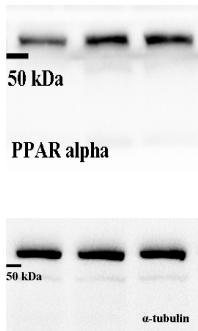

Appendix Fig S1C

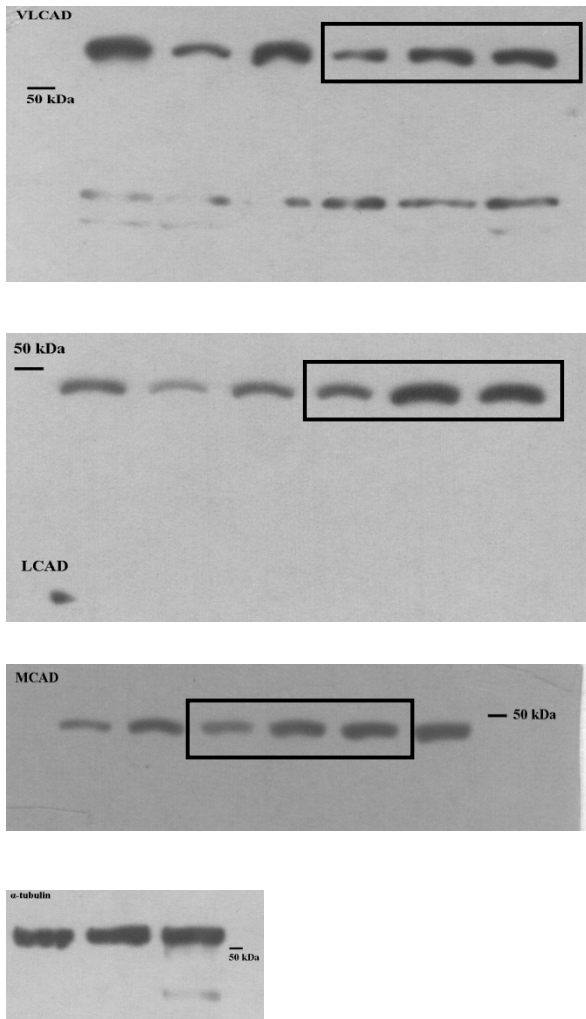

Appendix Fig S1F

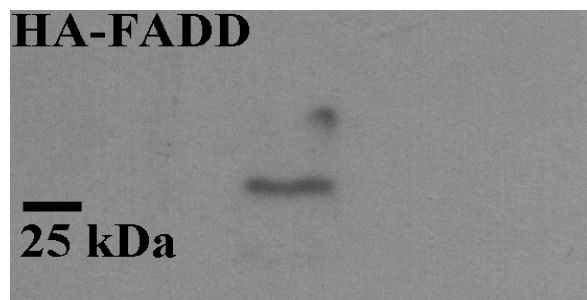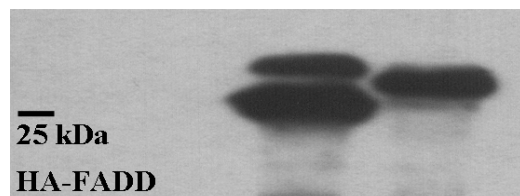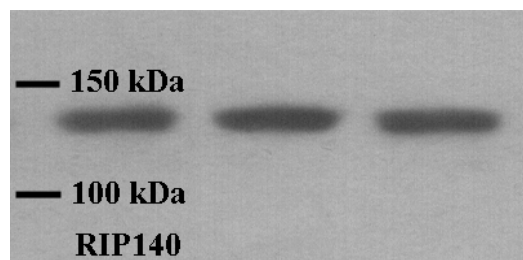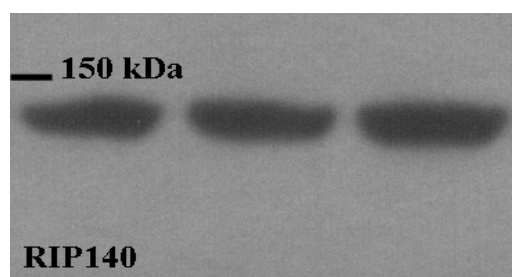

Appendix Fig S1G

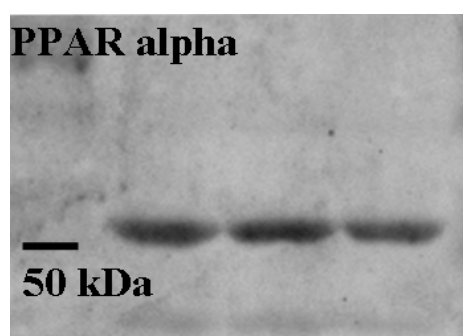

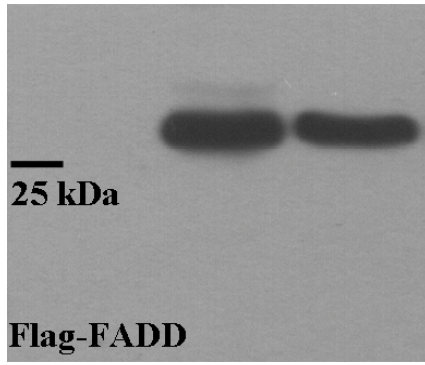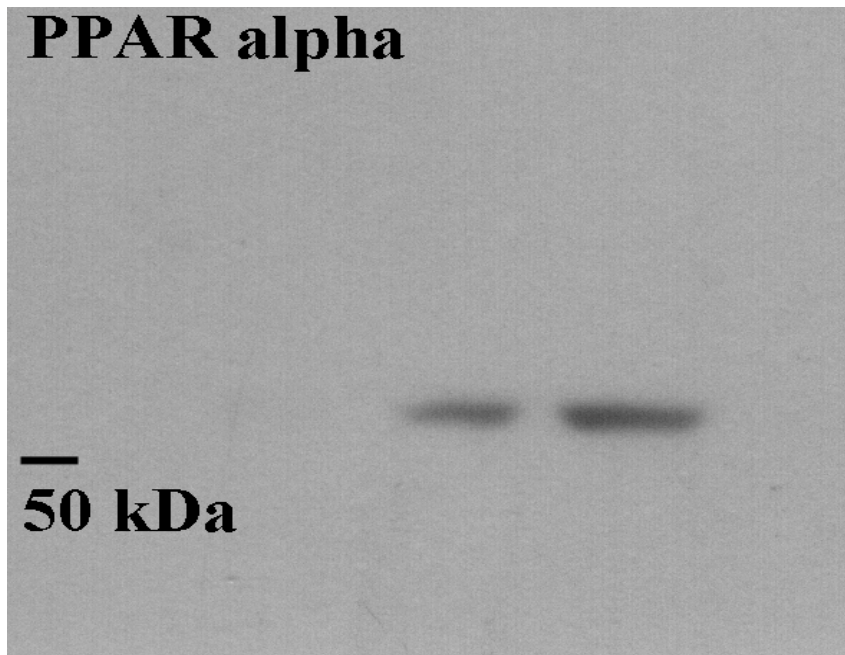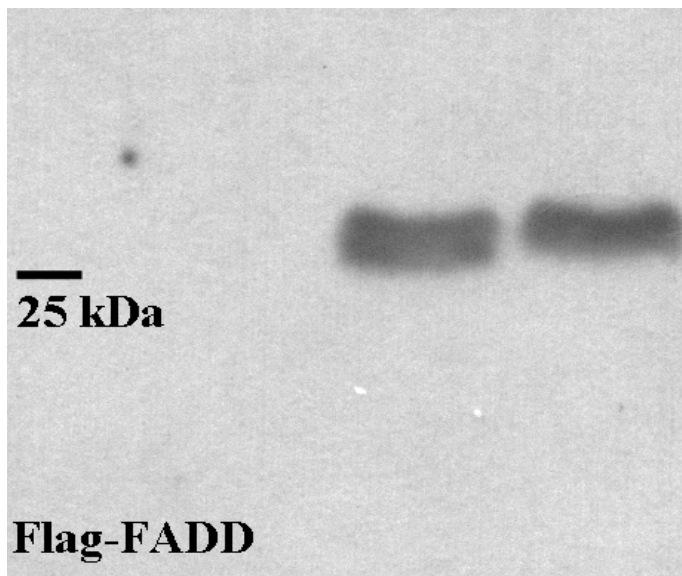

Appendix Fig S1H

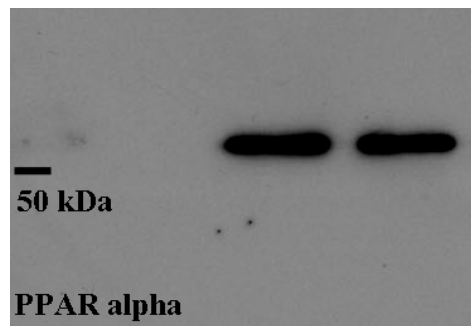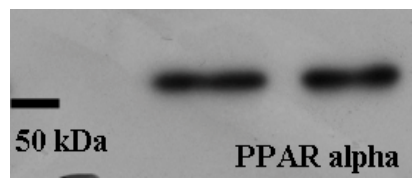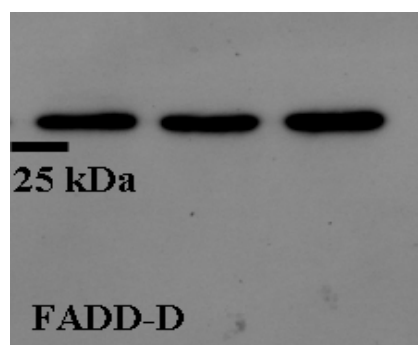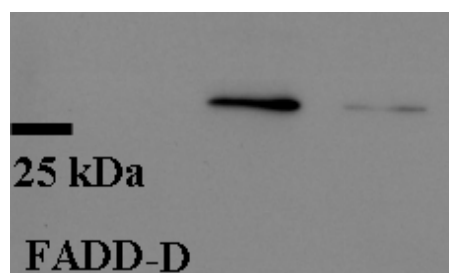

Supplement: Supplementary file 3 — Source Data for Appendix [file EMMM-8-895-s002.zip › Source_data_for_Expanded_View_and_Appendix/SourceDataForAppendixFigS1.pdf]

Appendix Fig S2A

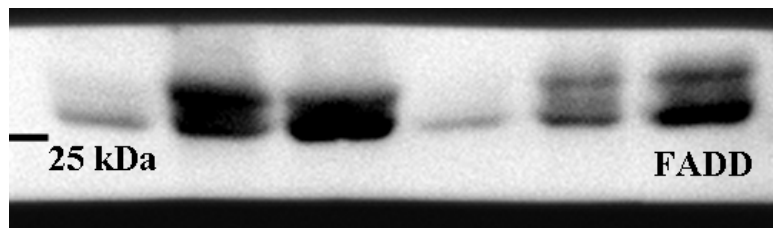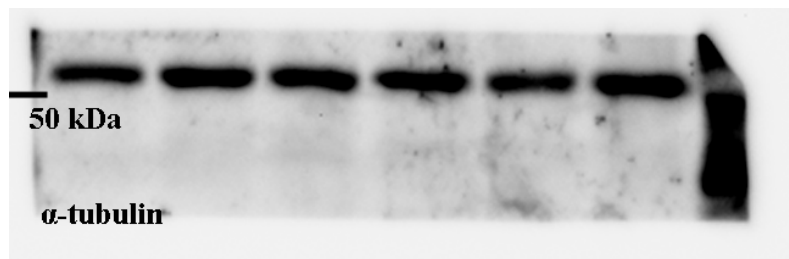

Appendix Fig S2B

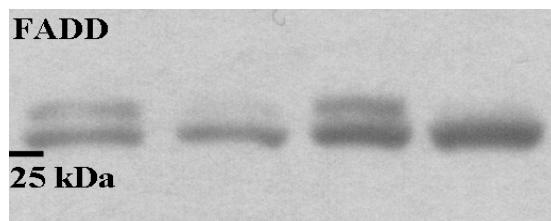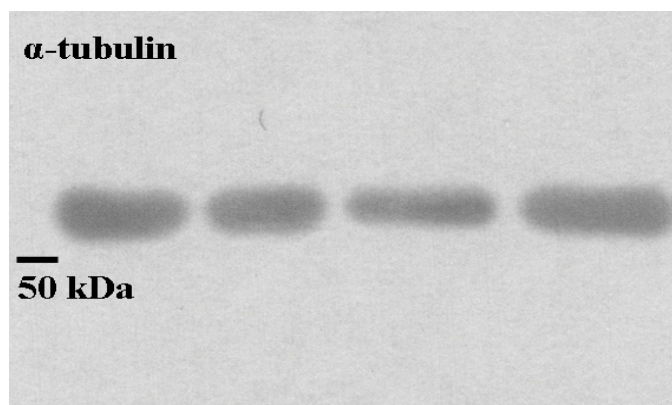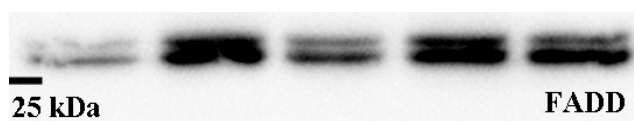

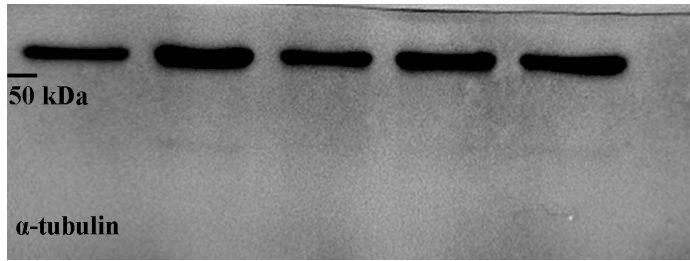

Appendix Fig S2C

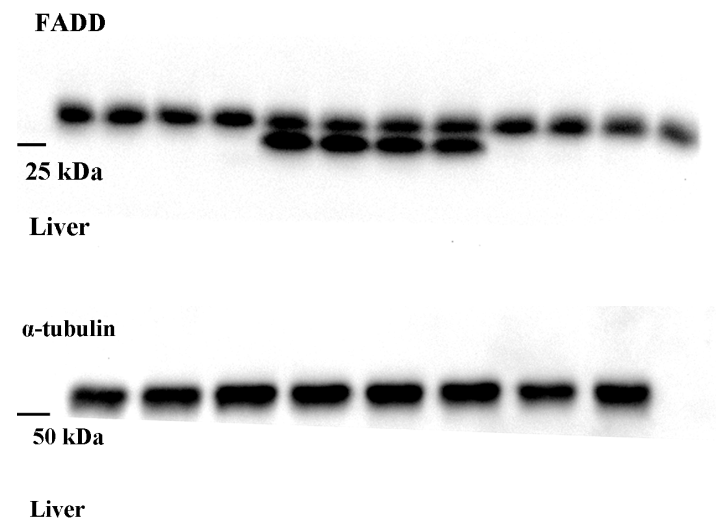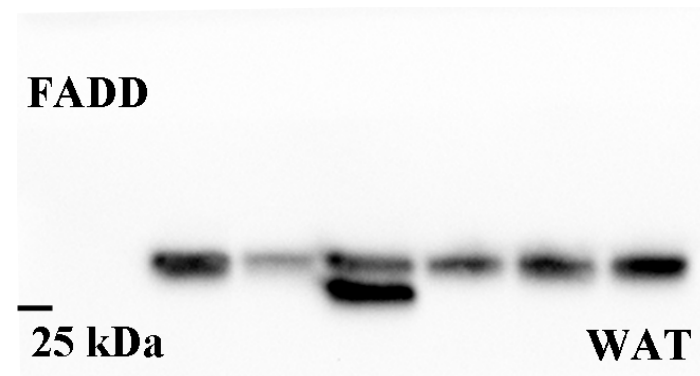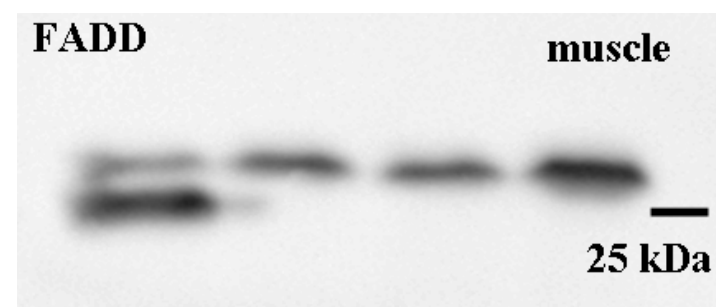

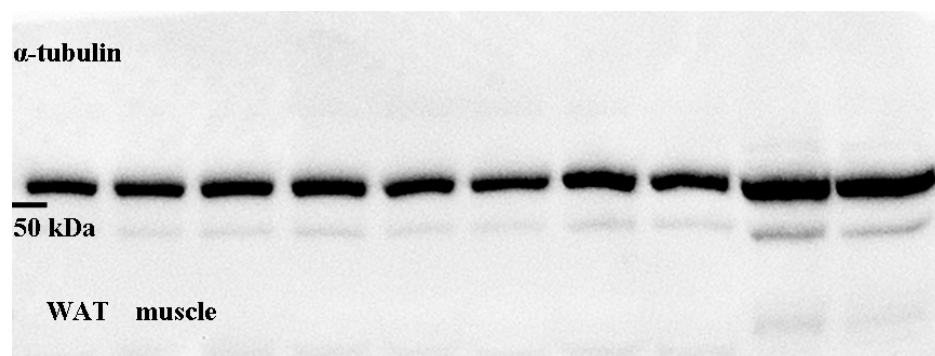

Supplement: Supplementary file 3 — Source Data for Appendix [file EMMM-8-895-s002.zip › Source_data_for_Expanded_View_and_Appendix/SourceDataForAppendixFigS2.pdf]

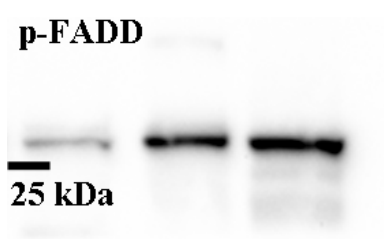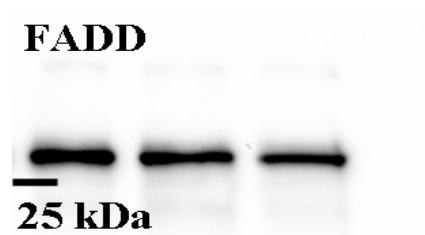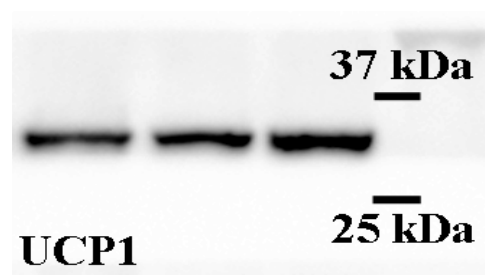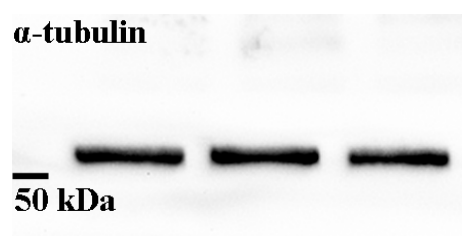

Supplement: Supplementary file 3 — Source Data for Appendix [file EMMM-8-895-s002.zip › Source_data_for_Expanded_View_and_Appendix/SourceDataForAppendixFigS4.pdf]

Appendix Fig S9A

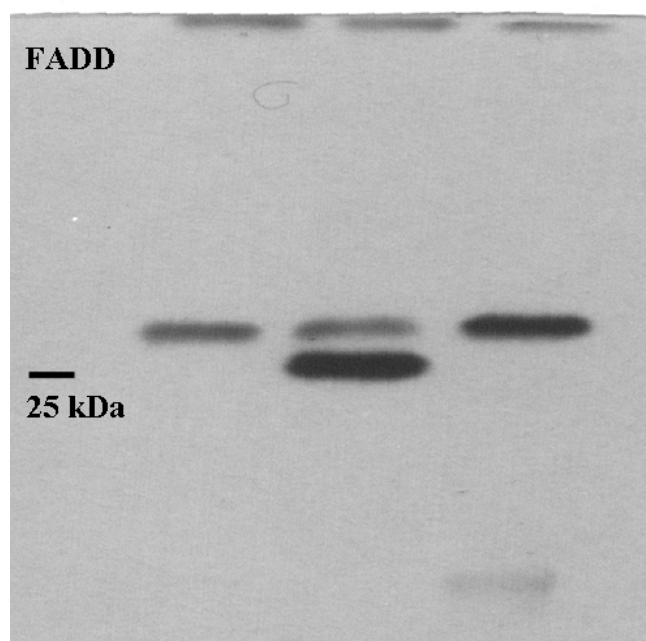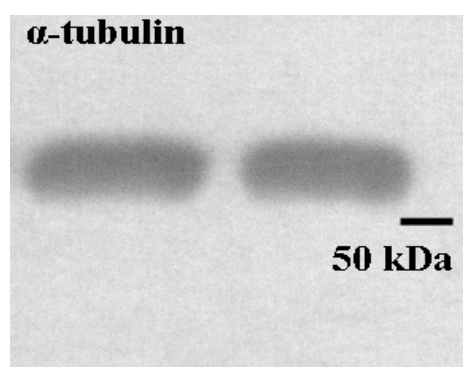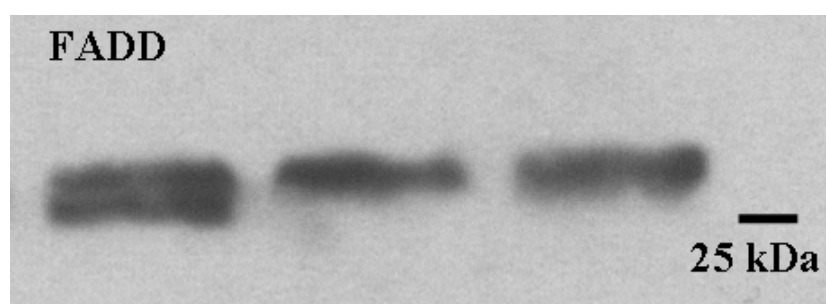

**$\alpha$ -tubulin**

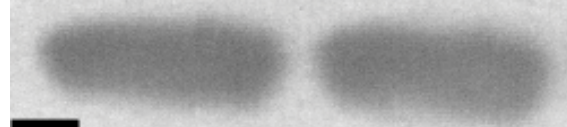

**50 kDa**

**FADD**

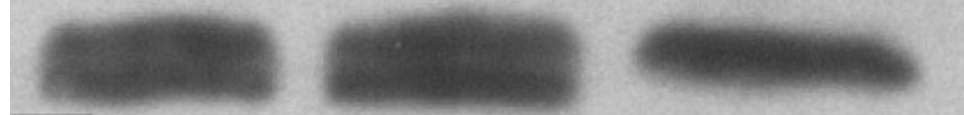

**25 kDa**

**$\alpha$ -tubulin**

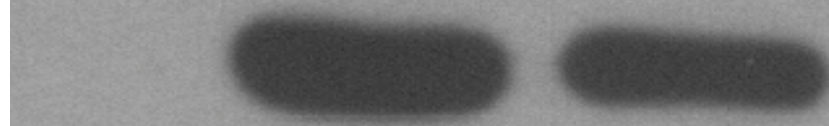

**50 kDa**

Appendix Fig S9D

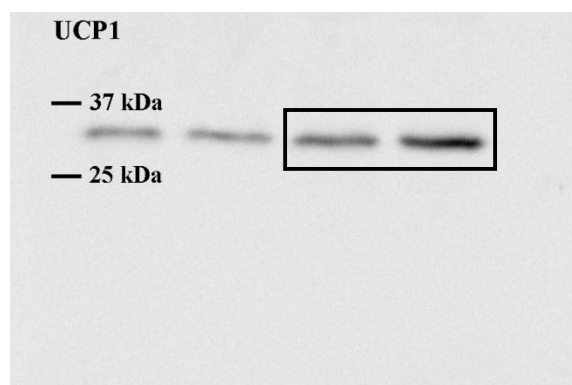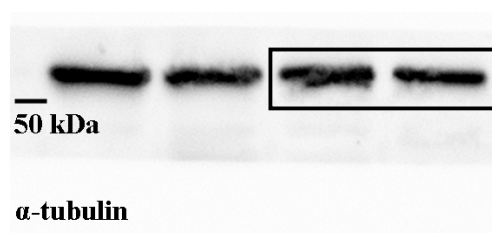

Supplement: Supplementary file 3 — Source Data for Appendix [file EMMM-8-895-s002.zip › Source_data_for_Expanded_View_and_Appendix/SourceDataForAppendixFigS9.pdf]

Figure 1A

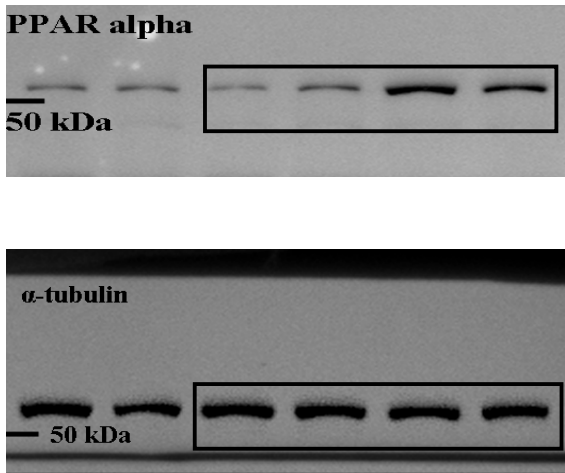

Figure 1F

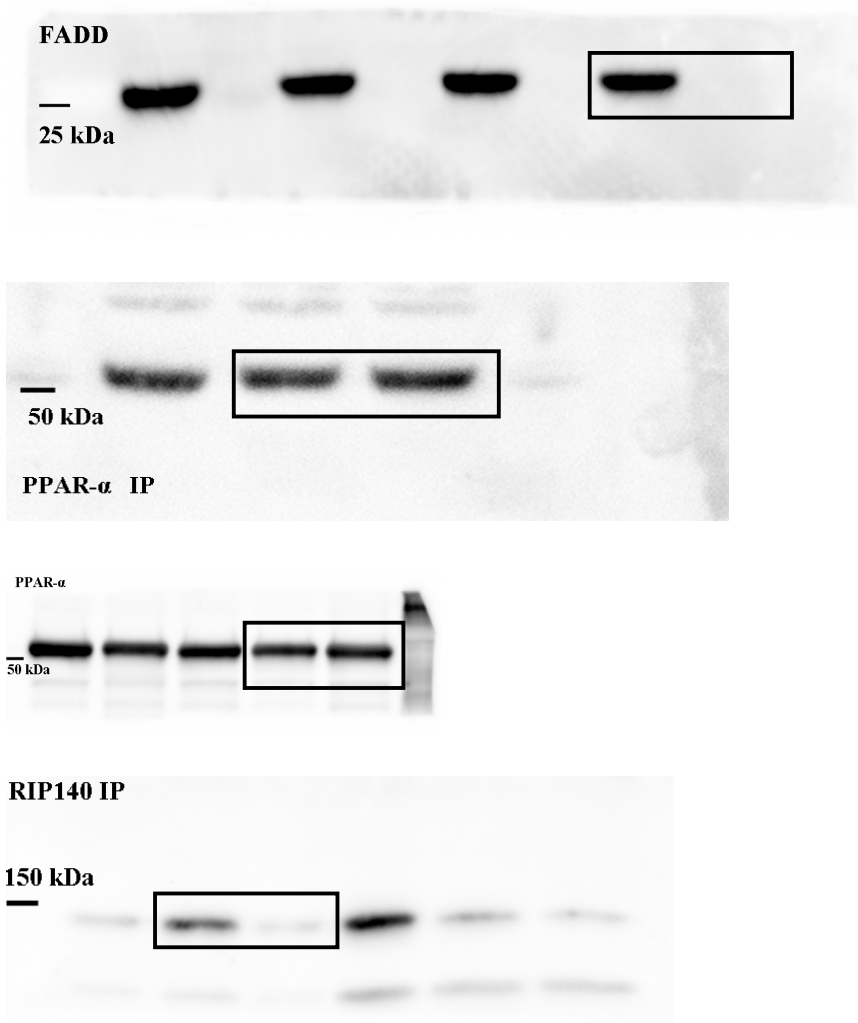

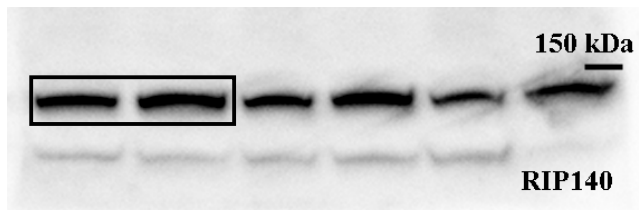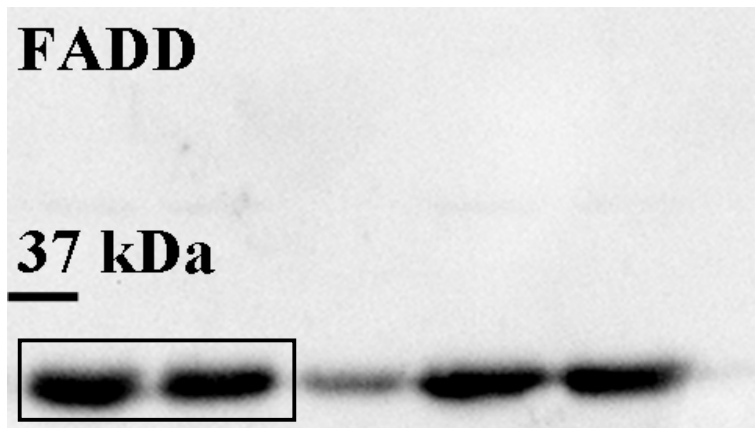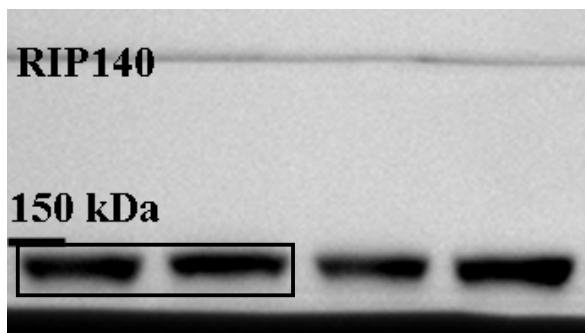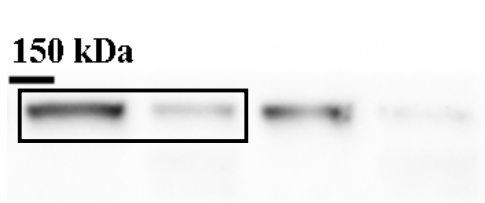

RIP140 IP

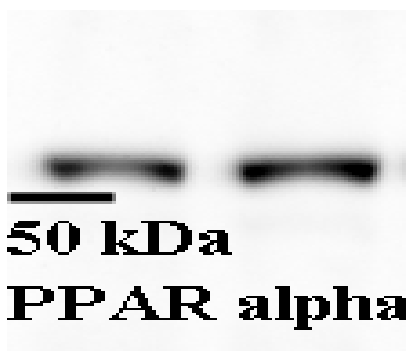

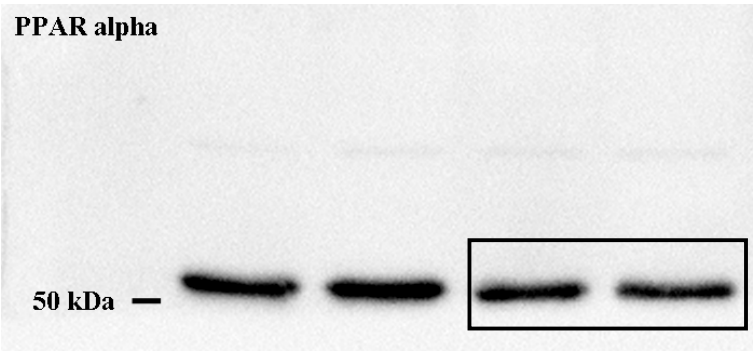

Figure 1G

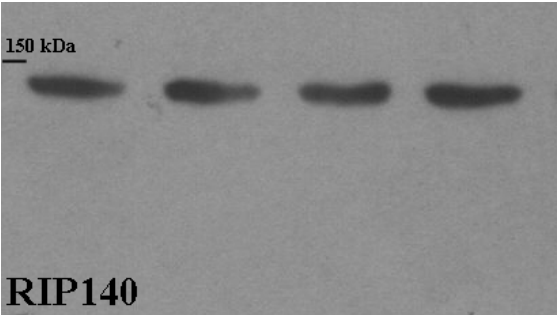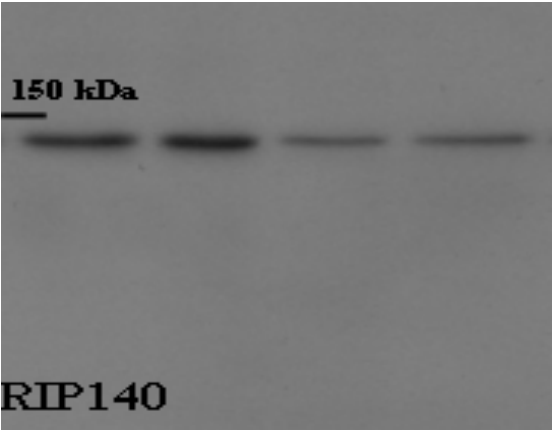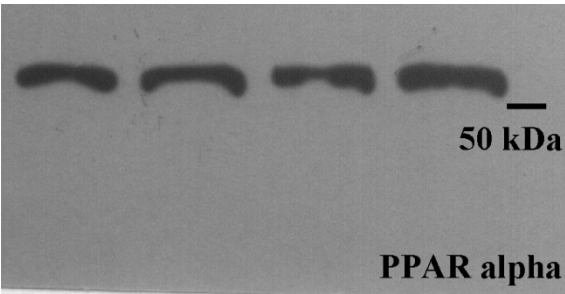

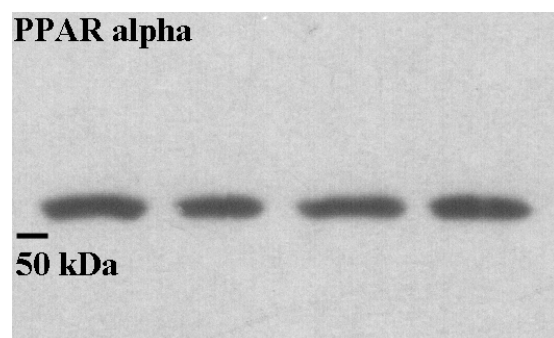

Figure 1H

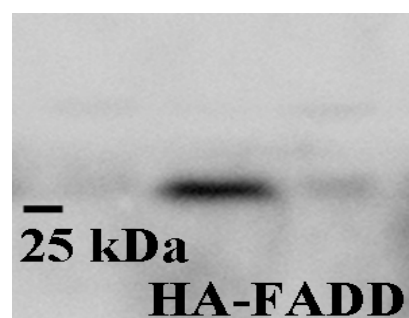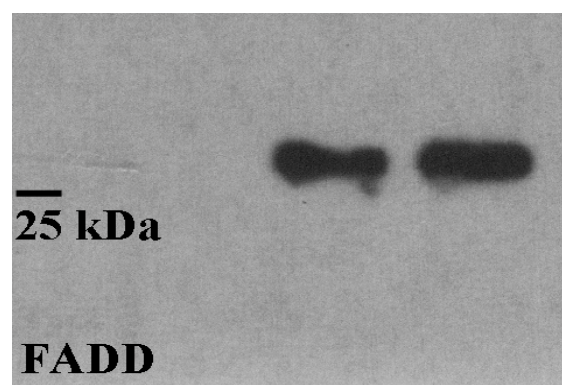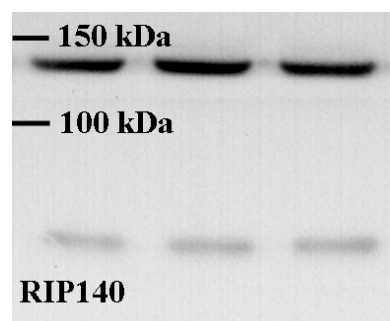

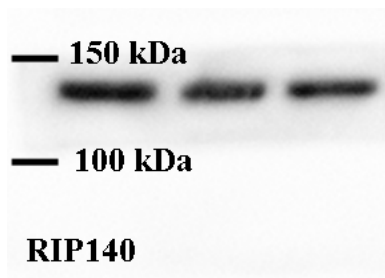

Supplement: Supplementary file 4 — Source Data for Figure 1 [file EMMM-8-895-s003.pdf]

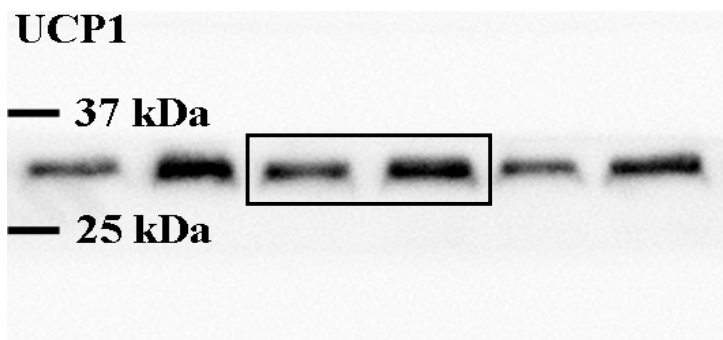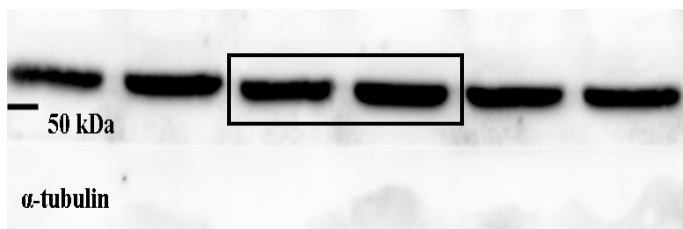

Supplement: Supplementary file 5 — Source Data for Figure 4 [file EMMM-8-895-s004.pdf]

Figure 5D

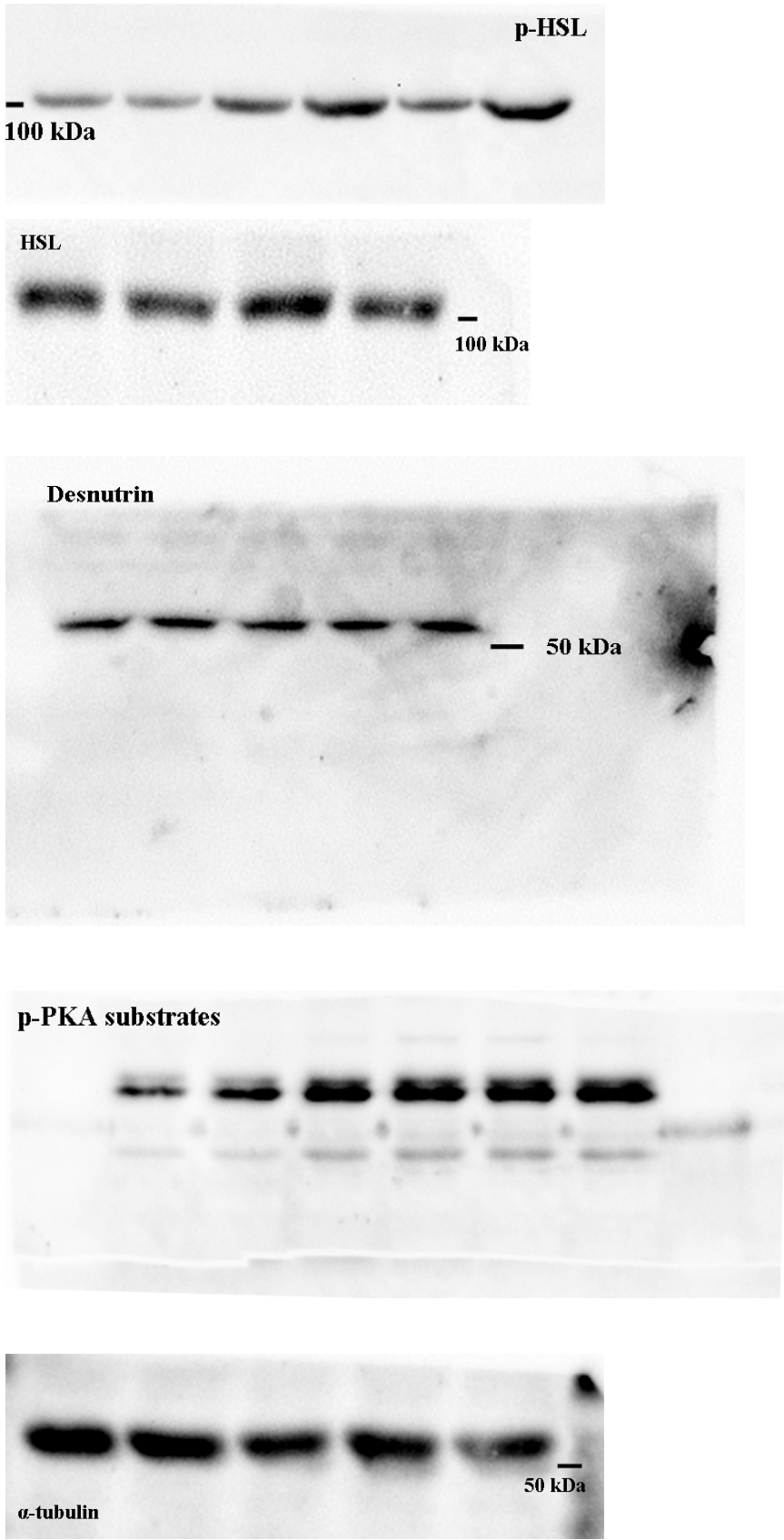

Figure 5G

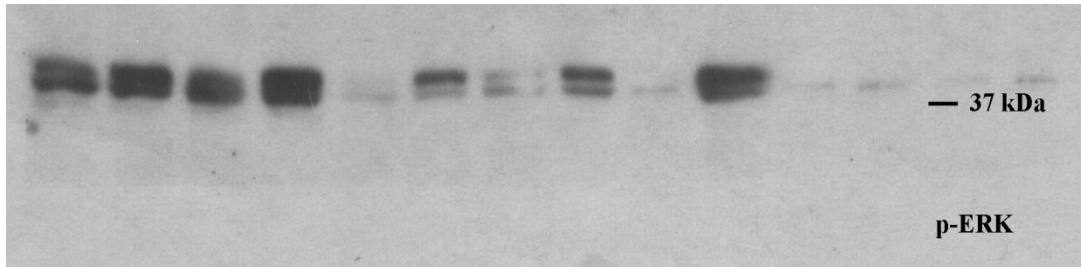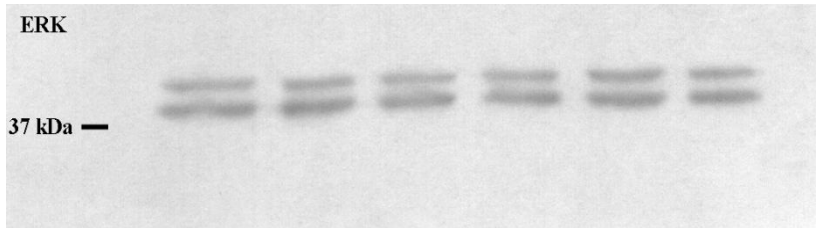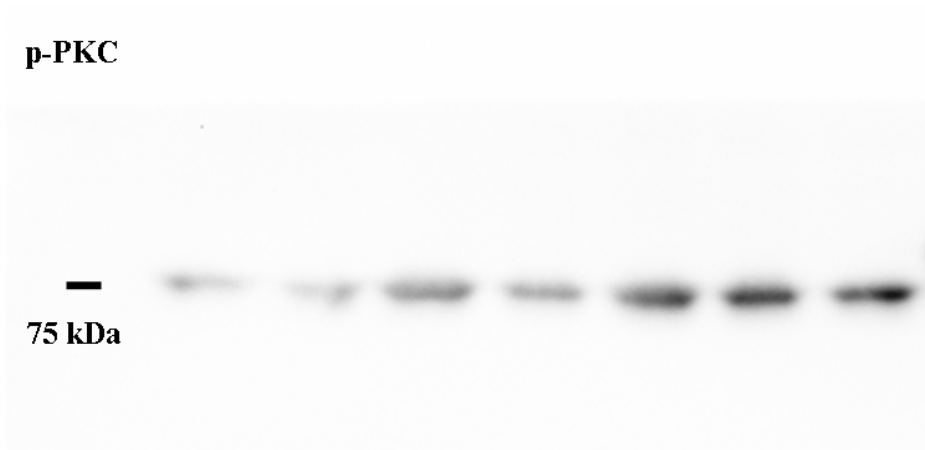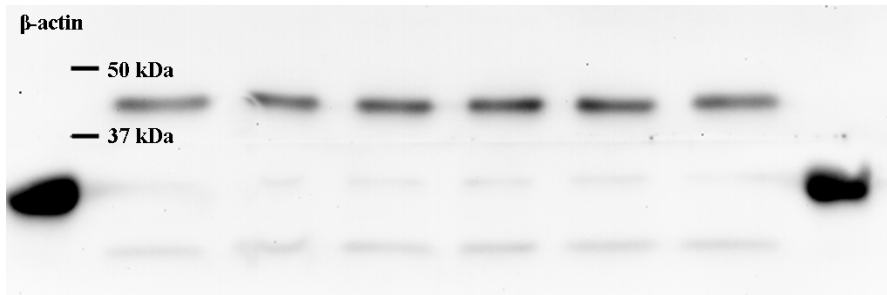

Supplement: Supplementary file 6 — Source Data for Figure 5 [file EMMM-8-895-s005.pdf]

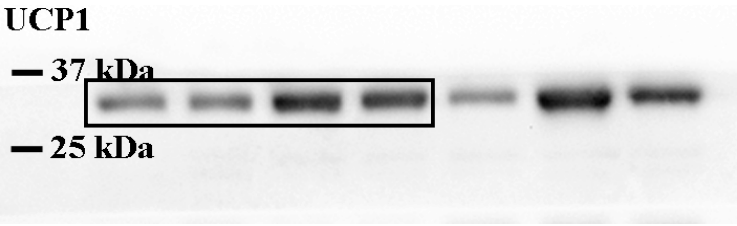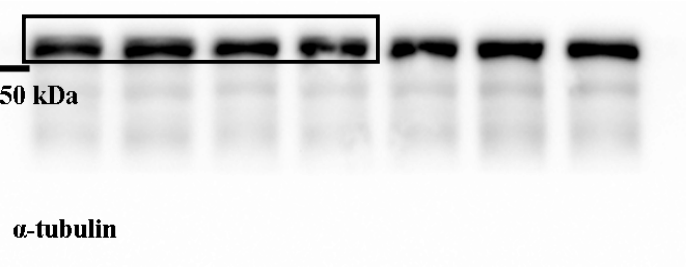

Supplement: Supplementary file 7 — Source Data for Figure 7 [file EMMM-8-895-s006.pdf]

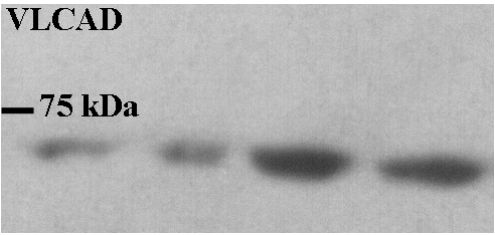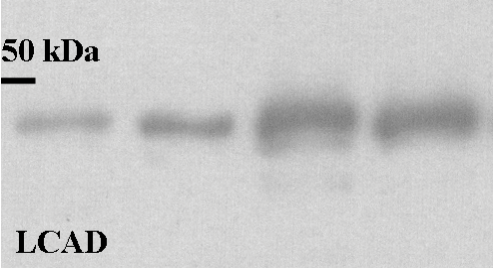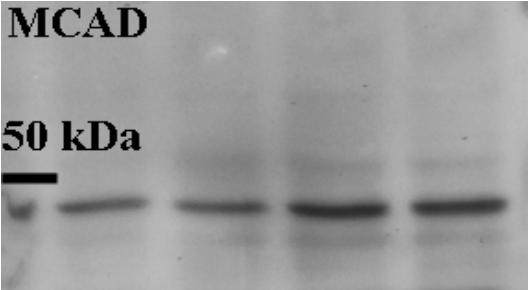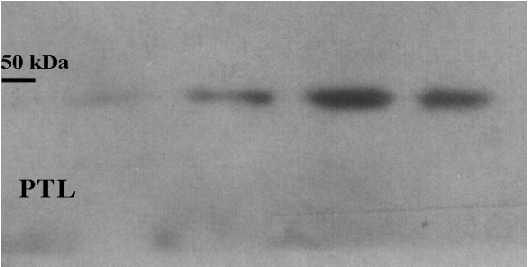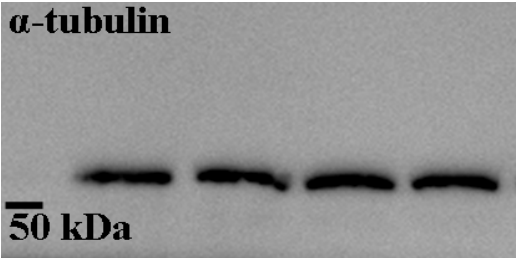

Supplement: Supplementary file 8 — Source Data for Figure 10 [file EMMM-8-895-s007.pdf]
