# Supplementary material for: FADD is a key regulator of lipid metabolism
Source: EMBO Mol Med. 2016 Jun 29;8(8):895–918. doi: 10.15252/emmm.201505924 (PMC4967943; doi:10.15252/emmm.201505924)
Supplement: Supplementary file 2 — Review Process File [file EMMM-8-895-s008.pdf]

EMBO Molecular Medicine

FADD is a key regulator of lipid metabolism

Hongqin Zhuang, Xueshi Wang, Daolong Zha, Ziyi Gan, Fangfang Cai, Pan Du, Yunwen Yang, Bingya Yang, Xiangyu Zhang, Chun Yao, Yuqiang Zhou, Chizhou Jiang, Shengwen Guan, Xuerui Zhang, Jing Zhang, Wenhui Jiang, Qingang Hu and Zi-Chun Hua

Corresponding author: Zi-Chun Hua, Nanjing University

Review timeline:

Submission date:	10 October 2015
Accepted:	30 May 2016

Editor: Céline Carret

Transaction Report:

No Peer Review Process File is available with this article, as the authors have chosen not to make the review process public in this case.

© EMBO
